# Supplementary material for: The P2 nucleic acid binding protein of Sugarcane bacilliform virus is a viral pathogenic factor
Source: PeerJ. 2024 Feb 20;12:e16982. doi: 10.7717/peerj.16982 (PMC10885806; doi:10.7717/peerj.16982)
Supplement: Supplemental Information 1 [file peerj-12-16982-s001.docx]

**SUPPLEMENTARY MATERIALS**

**TABLE S1. Primer sequences used in this study.**

| Primer name | Sequences* (5’-3’) | Purpose |
| --- | --- | --- |
| SCBV/F | GTCGTGGTATCAGAGCGAGGTATGAATTTTATCACAGTTATG | To amplify the complete genome of SCBV |
| SCBV/SmaI/R | GAGATGCCATGCCGACCCGGGCGACGAACCCCCCAAACCGG | To amplify the complete genome of SCBV |
| SCBV/0.06A/StuI/F | TCATTTCATTTGGAGAGGCCTGCAATGACGATCAGTGGCGAAG | To amplify 0.06 copies of SCBV genome |
| SCBV/0.06A/R | CTCGCTCTGATACCACGACGAACCCCCCAAACCGG | To amplify 0.06 copies of SCBV genome |
| SCBV/P2/ClaI/F | CCATCGATATGAGTATCAGTGAACCTGC | PVX-P2, for over-expression of P2 via pGR106 (PVX) vector |
| SCBV/P2/SalI/R | ACGCGTCGACTTATTGGATTTTCTTGAGAAGATCAAAGG | PVX-P2, for over-expression of P2 via pGR106 (PVX) vector |
| SCBV/P2/EcoRI/BamHI/F | CGGAATTCGGATCCATGAGTATCAGTGAACCTGC | pCHF3-P2, for transgenic expression and transient agroinfiltration |
| SCBV/P2/SacI/F | CGAGCTCATGAGTATCAGTGAACCTGC | pCHF3-P2-eGFP, for Subcellular localization of SCBV P2 |
| SCBV/P2-eGFP /BamHI/R | CGGGATCCTTGGATTTTCTTGAGAAGATCAAAGG | pCHF3-P2-eGFP, for Subcellular localization of SCBV P2 |
| X-eGFP/BamHI/F | CGGGATCCATGGTGAGCAAGGGCGAGGAG | pCHF3-P2-eGFP, for Subcellular localization of SCBV P2 |
| X-eGFP/SalI/R | ACGCGTCGACTTACTTGTACAGCTCGTCCATGC | pCHF3-P2-eGFP, for Subcellular localization of SCBV P2 |
| *Nb*β-actin/F | TGGACTCTGGTGATGGTGTC | Relative RT-qPCR analysis of *Nb*β-actin |
| *Nb*β-actin/R | GACCTCAGGACAACGGAAACG |  |
| *Nb*MET1 /F | TGAATAGTTTGCCACTGCCAGGAC | Relative RT-qPCR analysis of *Nb*MET1 |
| *Nb*MET1 /R | AGCGAAATGTTTGTTTTTGGTTGA |  |
| *Nb*DRM2/F | AAGGCAAAGGCGATTTTTGAGGAAG | Relative RT-qPCR analysis of *Nb*DRM2 |
| *Nb*DRM2/R | CTTTGTCCGTGGATCCCAAGATG |  |
| *Nb*CMT3/F | CTTACGGTCTTCCACAGTTTCG | Relative RT-qPCR analysis of *Nb*CMT3 |
| *Nb*CMT3/R | CCATTTCATCTCTTTGCTCATT |  |
| *Nb*ROS1/F | GAAGGAACTGAATCAAGCAACT | Relative RT-qPCR analysis of *Nb*ROS1 |
| *Nb*ROS1/R | GGCATCAGACATAAGTCCAAAT |  |
| *Nb*ROS2/F | ATTCAAACGAAGAAAAAACAGC | Relative RT-qPCR analysis of *Nb*ROS2 |
| *Nb*ROS2/R | CAGAACTCGGAGAAGAAGGCAA |  |
| *Nb*DCL3/F | CTACAAGCAGAGAAGGATCATGGAA | Relative RT-qPCR analysis of *Nb*DCL3 |
| *Nb*DCL3/R | GAGTCAACAGAGCGTAAATCCAAGT |  |
| *Nb*AGO1/F | CTGGCGTGGCTTCTATCAAAGTATT | Relative RT-qPCR analysis of *Nb*AGO1 |
| *Nb*AGO1/R | CACCTTTACACCTCTCAGTGCCTTC |  |
| *Nb*AGO4/F | GGAACTATGACTTCTACCTGTGTGCC | Relative RT-qPCR analysis of *Nb*AGO4 |
| *Nb*AGO4/R | AACTTCATCCATTGTCCAACTTGTGT |  |
| *Nb*HDA6/F | AGATTGCCCTGTTTTTGATGGAC | Relative RT-qPCR analysis of *Nb*HDA6 |
| *Nb*HDA6/R | ACACGCTTGTGGACCTTGAG |  |

*Restriction sites are underlined.
