# Supplementary figures and images for: The P2 nucleic acid binding protein of Sugarcane bacilliform virus is a viral pathogenic factor
Source: PeerJ. 2024 Feb 20;12:e16982. doi: 10.7717/peerj.16982 (PMC10885806; doi:10.7717/peerj.16982)

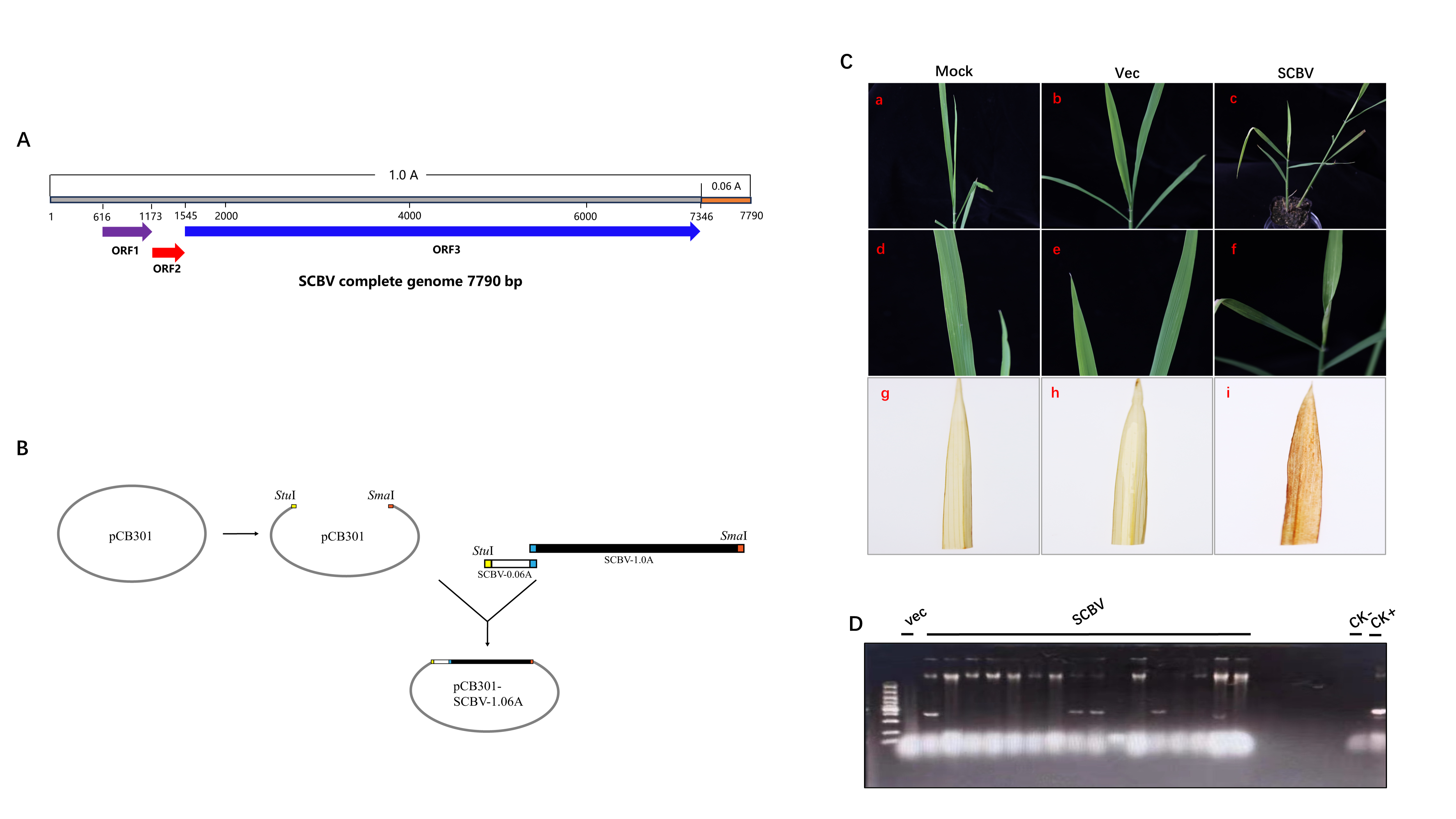

Supplement: Supplemental Information 2 — (A) The complete genome struct of SCBV. (B) The construction strategy of SCBV infectious clone. (C) Symptoms elicited on Oryza sativa plants at 21 dpi infected with SCBV infectious clone and pCB301 empty vector (a-f). Necrotic lesions on Oryza sativa leaves induced by SCBV were photographed directly at 21 dpi and photographed after 3,3′-diaminobenzidine (DAB) staining (g-i). (D) SCBV detection on SCBV inoculated Oryza sativa plants at 21 dpi by PCR using RT/RNase H region specific primer pairs. [file peerj-12-16982-s002.png]

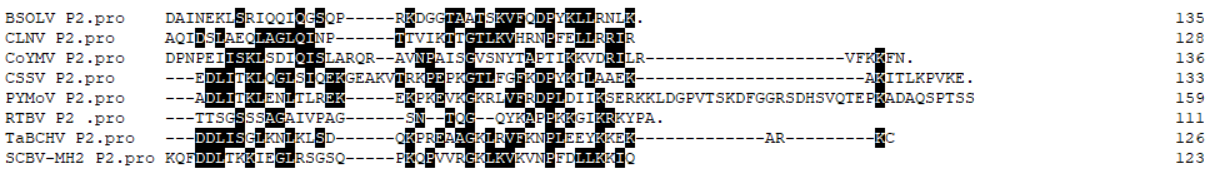

Supplement: Supplemental Information 3 [file peerj-12-16982-s003.zip › badnavirus p2 align-2.PNG]

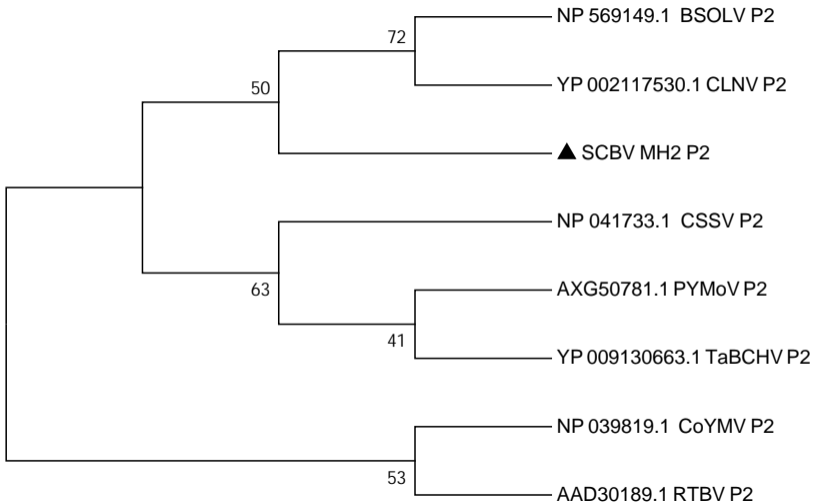

Supplement: Supplemental Information 3 [file peerj-12-16982-s003.zip › badnavirus p2 phylogenetic tree-Bootstrap.PDF]

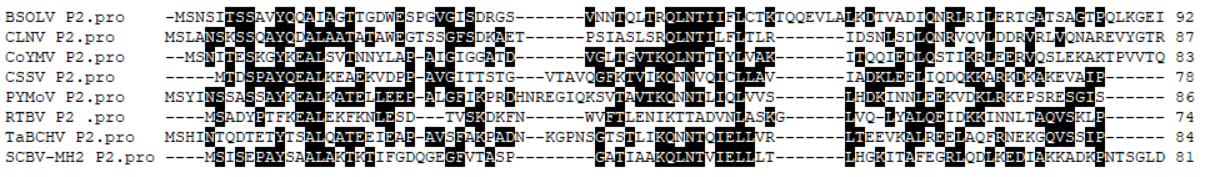

Supplement: Supplemental Information 3 [file peerj-12-16982-s003.zip › badnavirus p2 align-1.PNG]

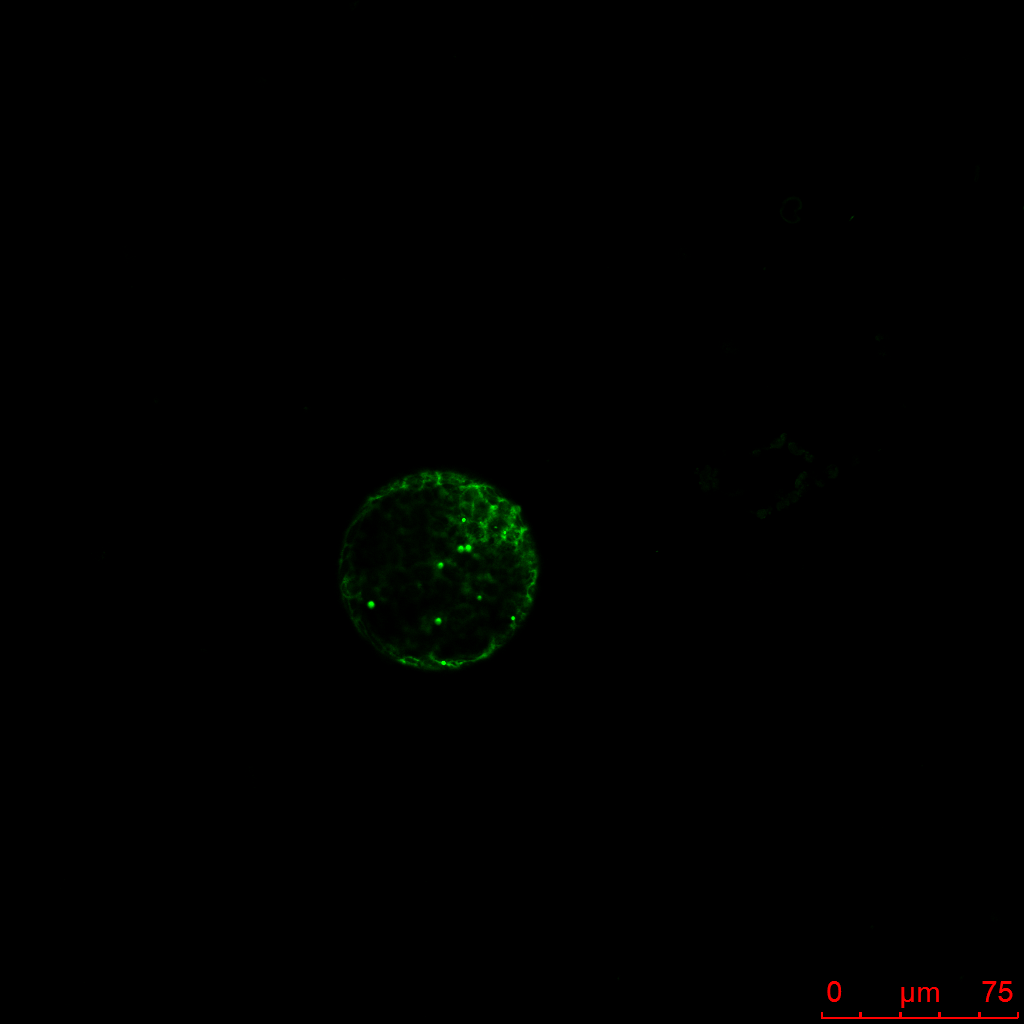

Supplement: Supplemental Information 4 [file peerj-12-16982-s004.zip › Fig 2-Subcellular localization/Protoplast GFP P2-eGFP.tif]

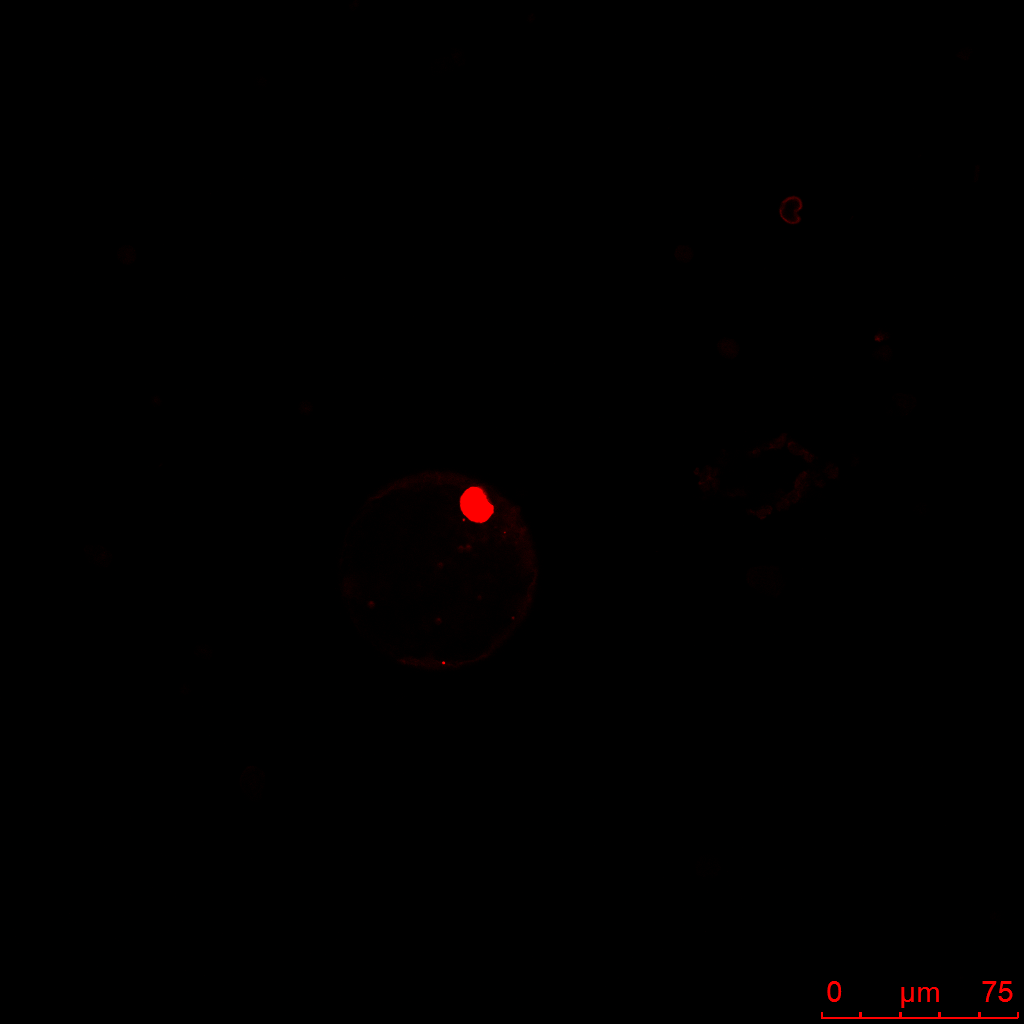

Supplement: Supplemental Information 4 [file peerj-12-16982-s004.zip › Fig 2-Subcellular localization/Protoplast RFP P2-eGFP.tif]

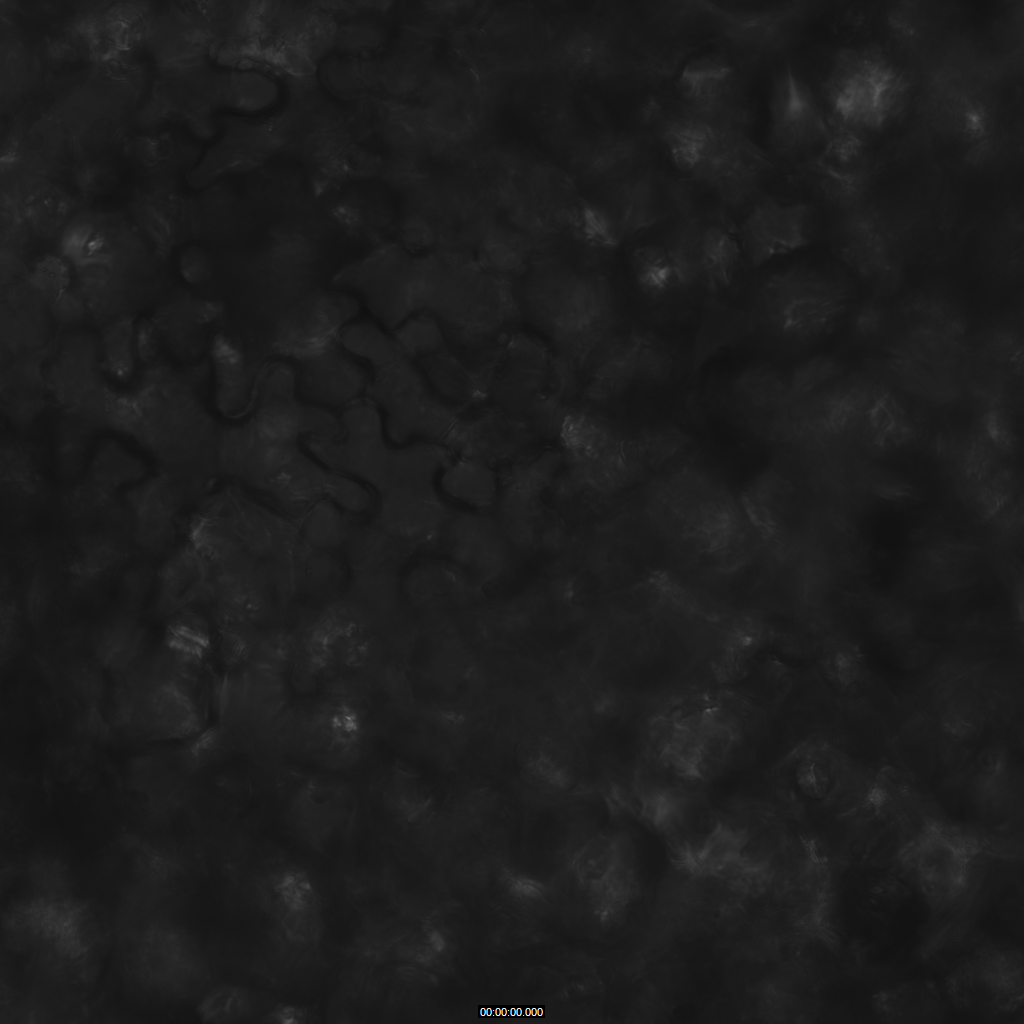

Supplement: Supplemental Information 4 [file peerj-12-16982-s004.zip › Fig 2-Subcellular localization/bright P2-eGFP.tif]

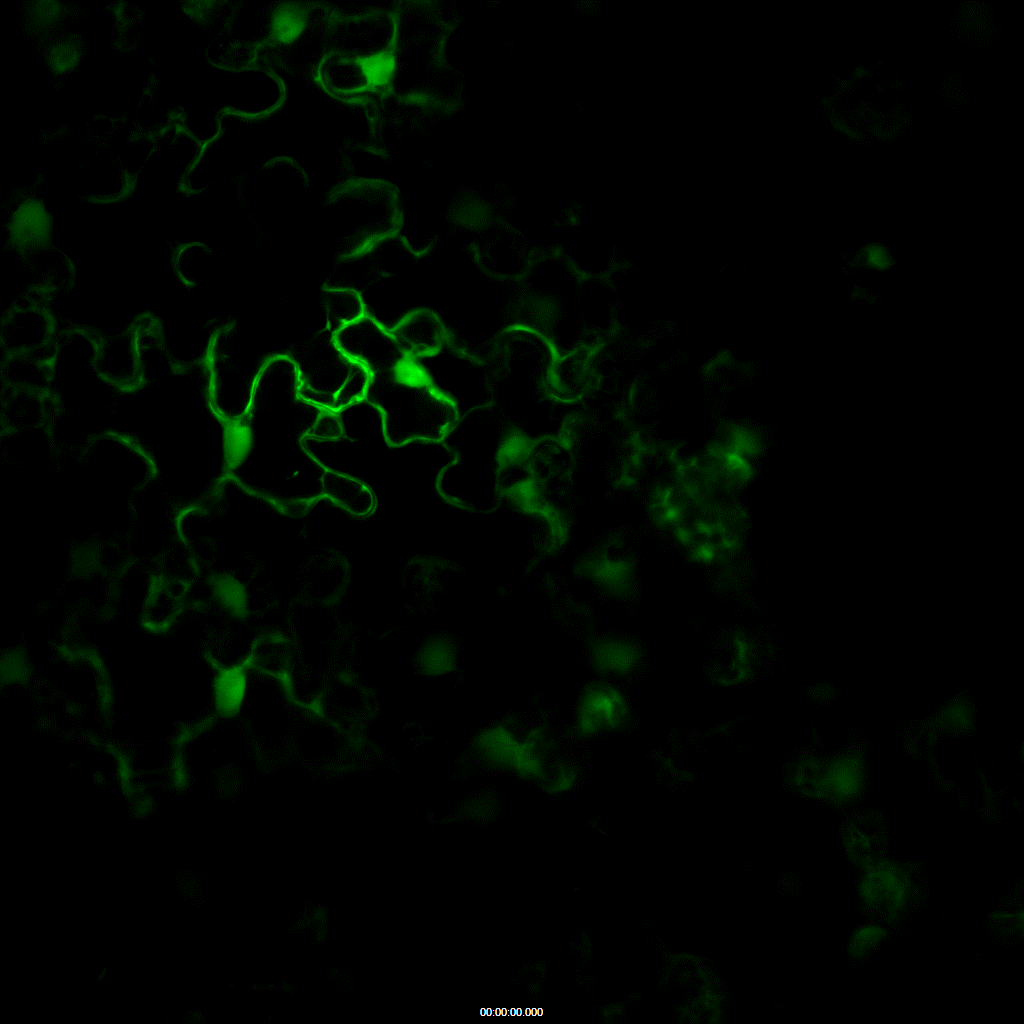

Supplement: Supplemental Information 4 [file peerj-12-16982-s004.zip › Fig 2-Subcellular localization/GFP P2-eGFP.png]

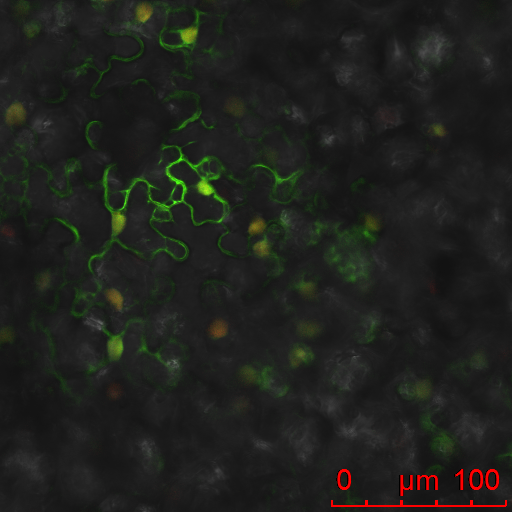

Supplement: Supplemental Information 4 [file peerj-12-16982-s004.zip › Fig 2-Subcellular localization/Merge P2-egfp.tif]

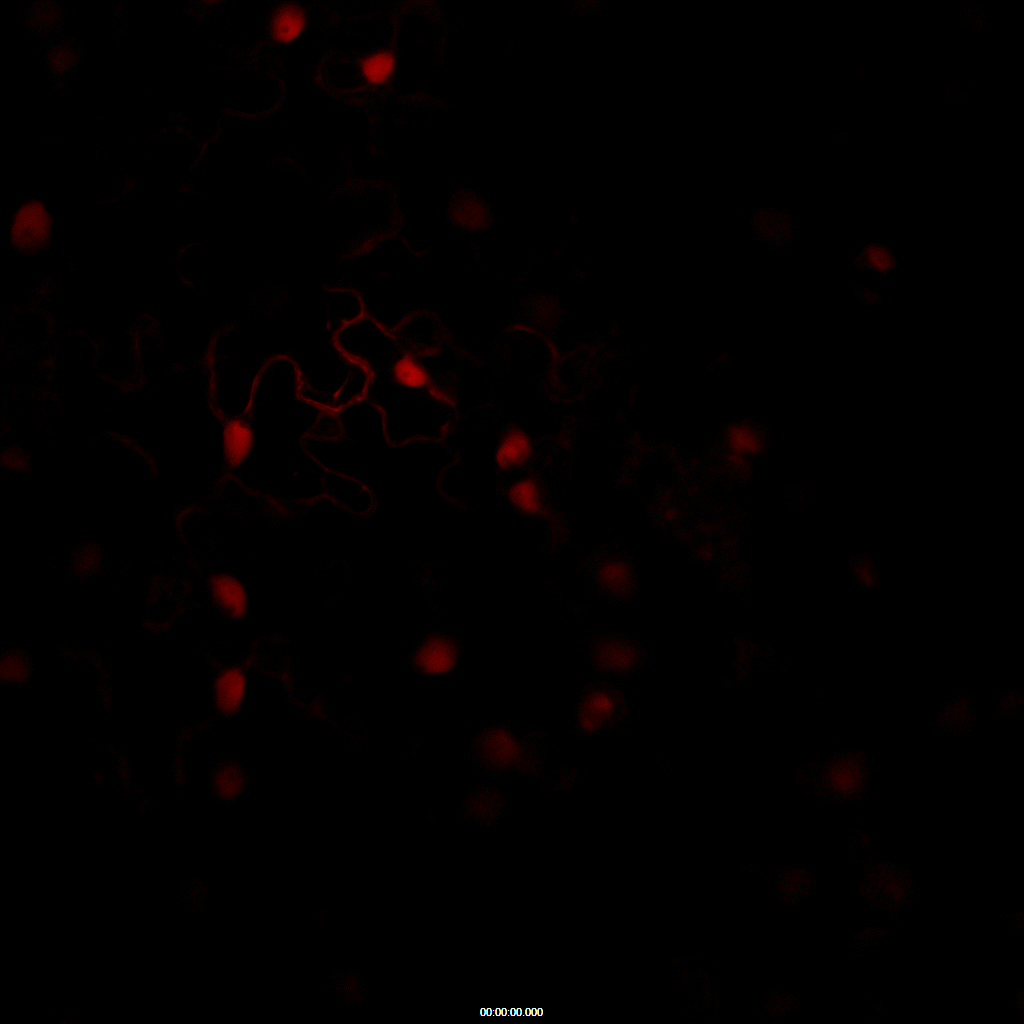

Supplement: Supplemental Information 4 [file peerj-12-16982-s004.zip › Fig 2-Subcellular localization/RFP P2-egfp.png]

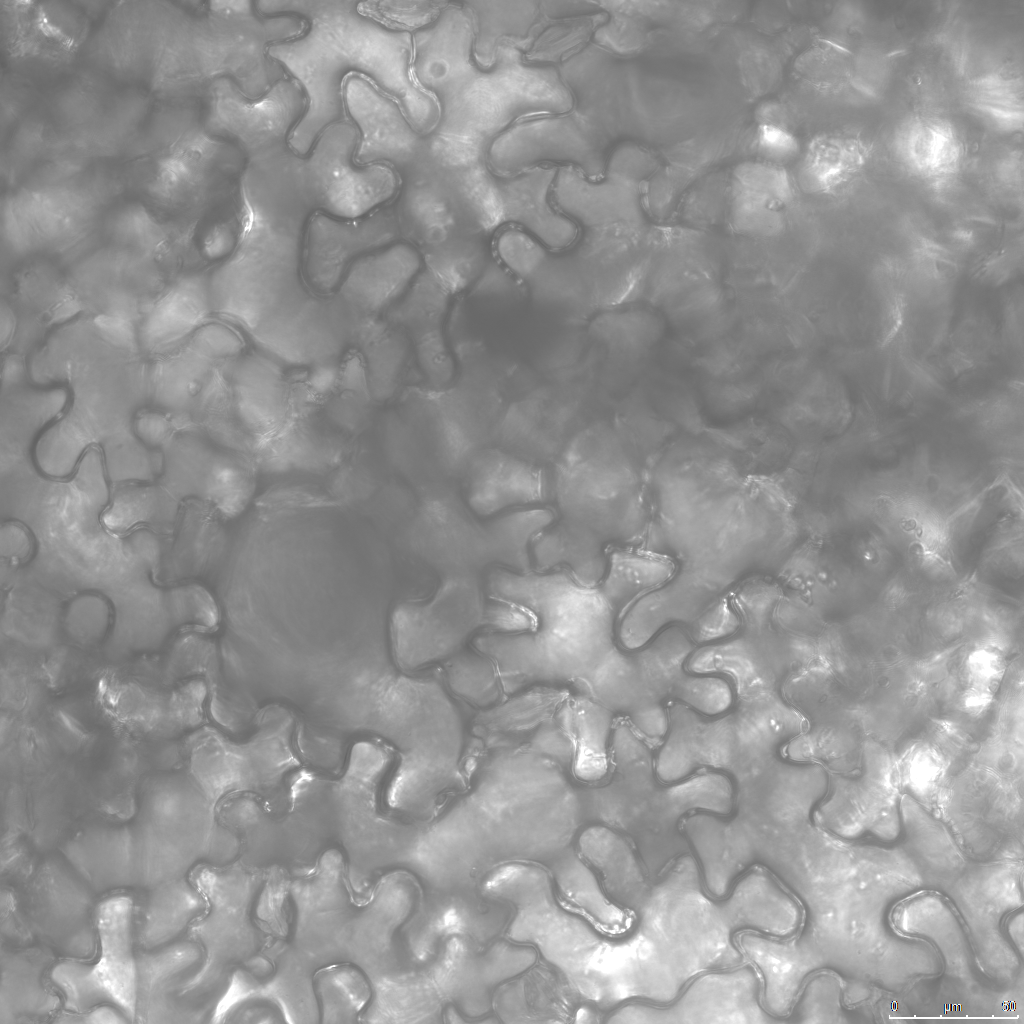

Supplement: Supplemental Information 4 [file peerj-12-16982-s004.zip › Fig 2-Subcellular localization/Bright 35s-eGFP.tif]

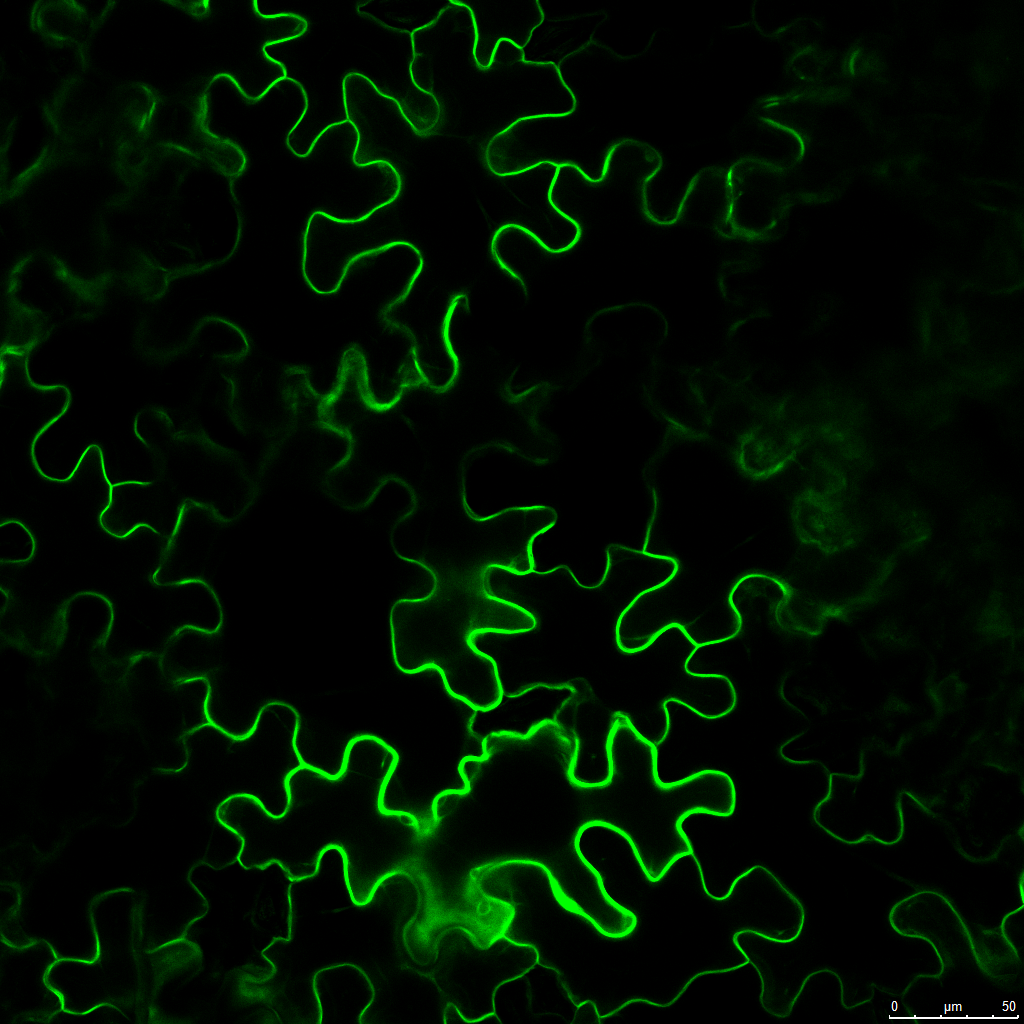

Supplement: Supplemental Information 4 [file peerj-12-16982-s004.zip › Fig 2-Subcellular localization/GFP 35s-eGFP.tif]

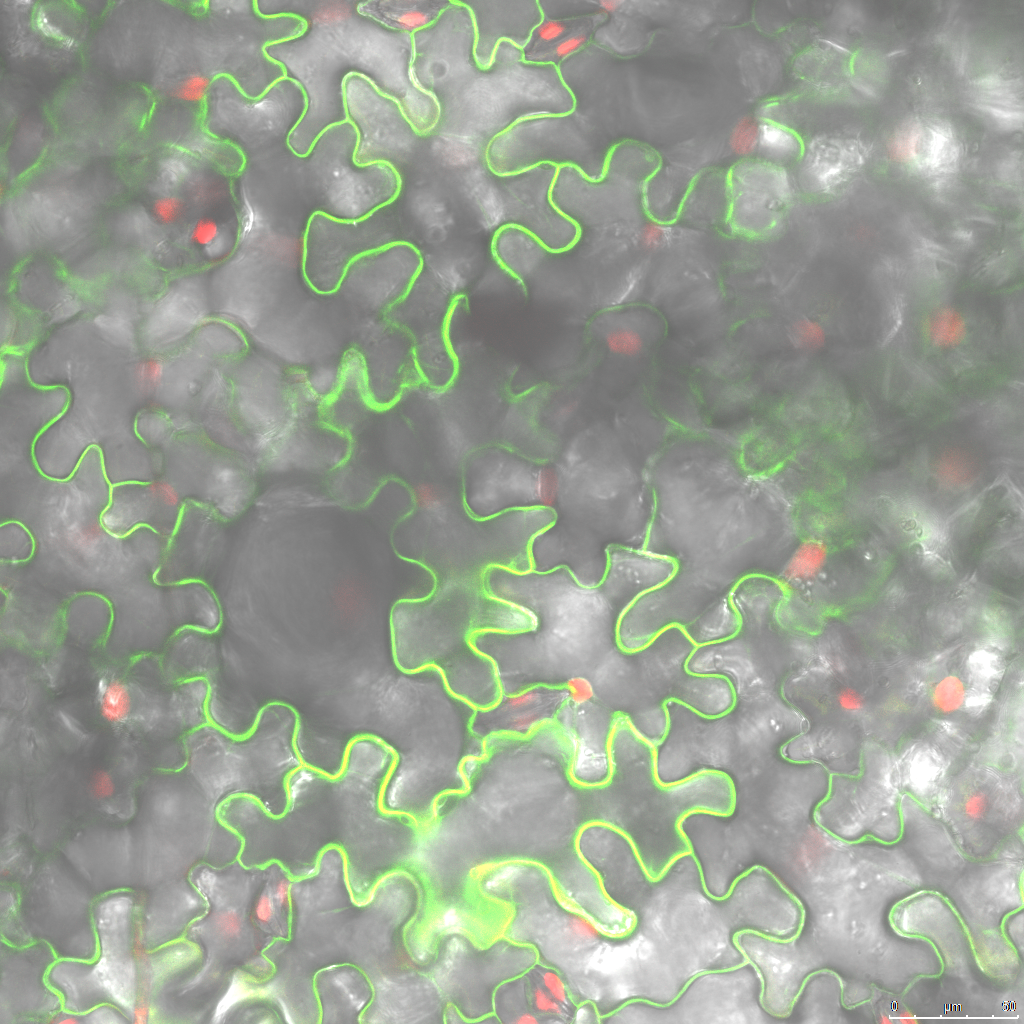

Supplement: Supplemental Information 4 [file peerj-12-16982-s004.zip › Fig 2-Subcellular localization/Merge 35s-eGFP.tif]

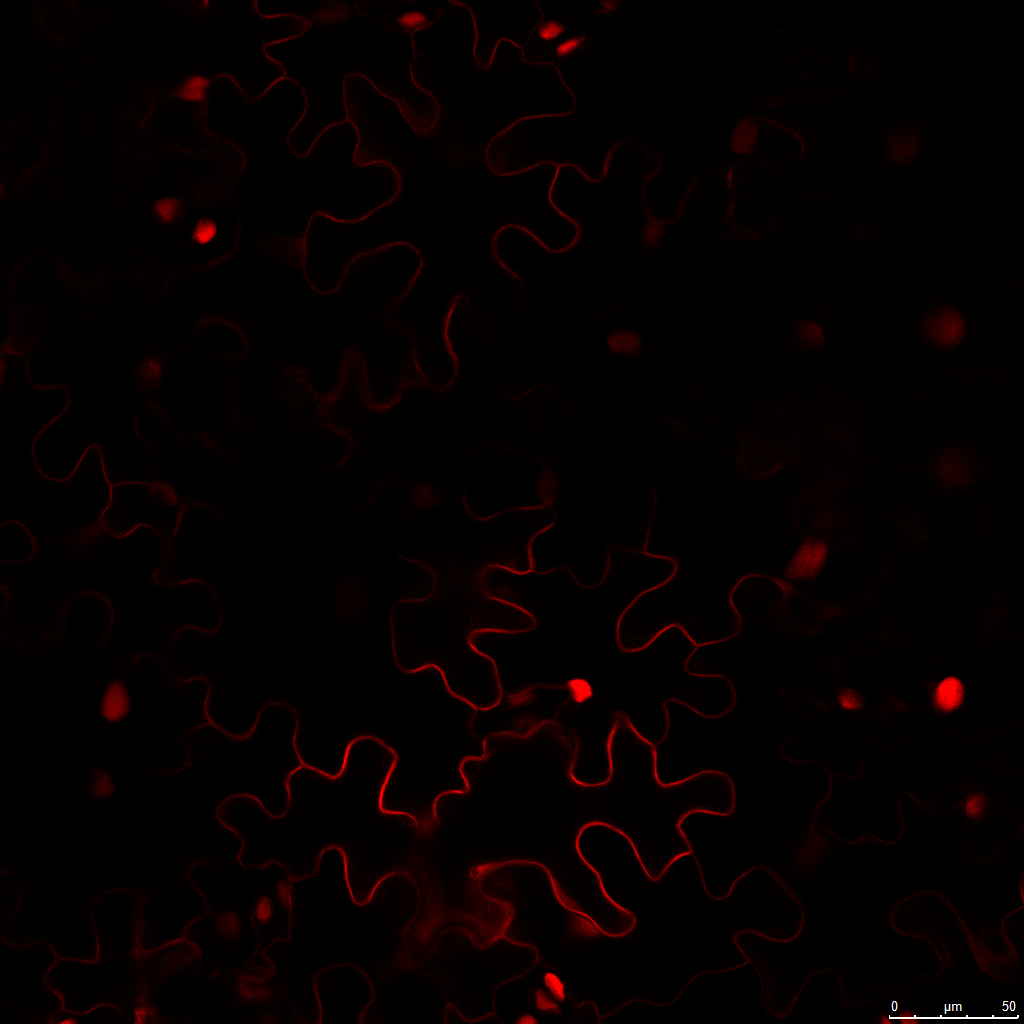

Supplement: Supplemental Information 4 [file peerj-12-16982-s004.zip › Fig 2-Subcellular localization/RFP 35s-eGFP.tif]

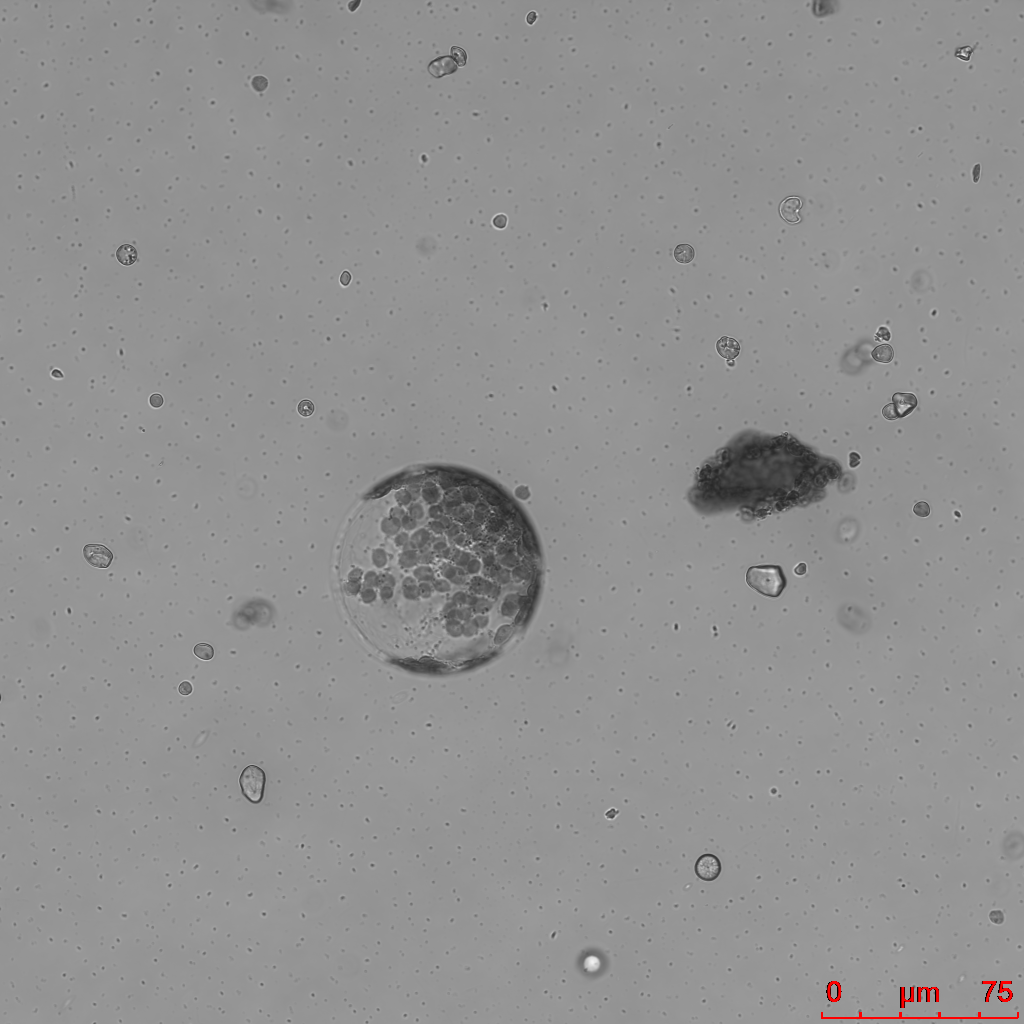

Supplement: Supplemental Information 4 [file peerj-12-16982-s004.zip › Fig 2-Subcellular localization/Protoplast bright P2-eGFP.tif]

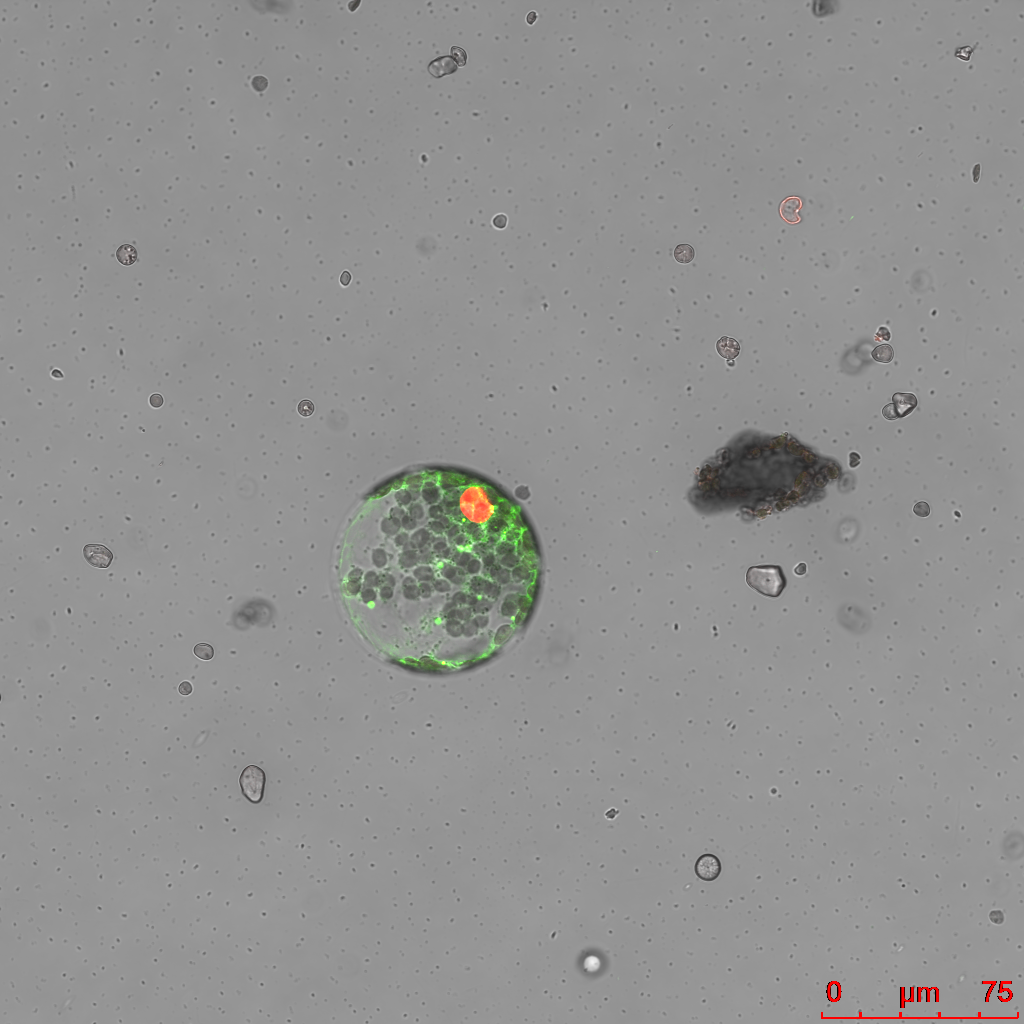

Supplement: Supplemental Information 4 [file peerj-12-16982-s004.zip › Fig 2-Subcellular localization/Protoplast Merge P2-eGFP.tif]

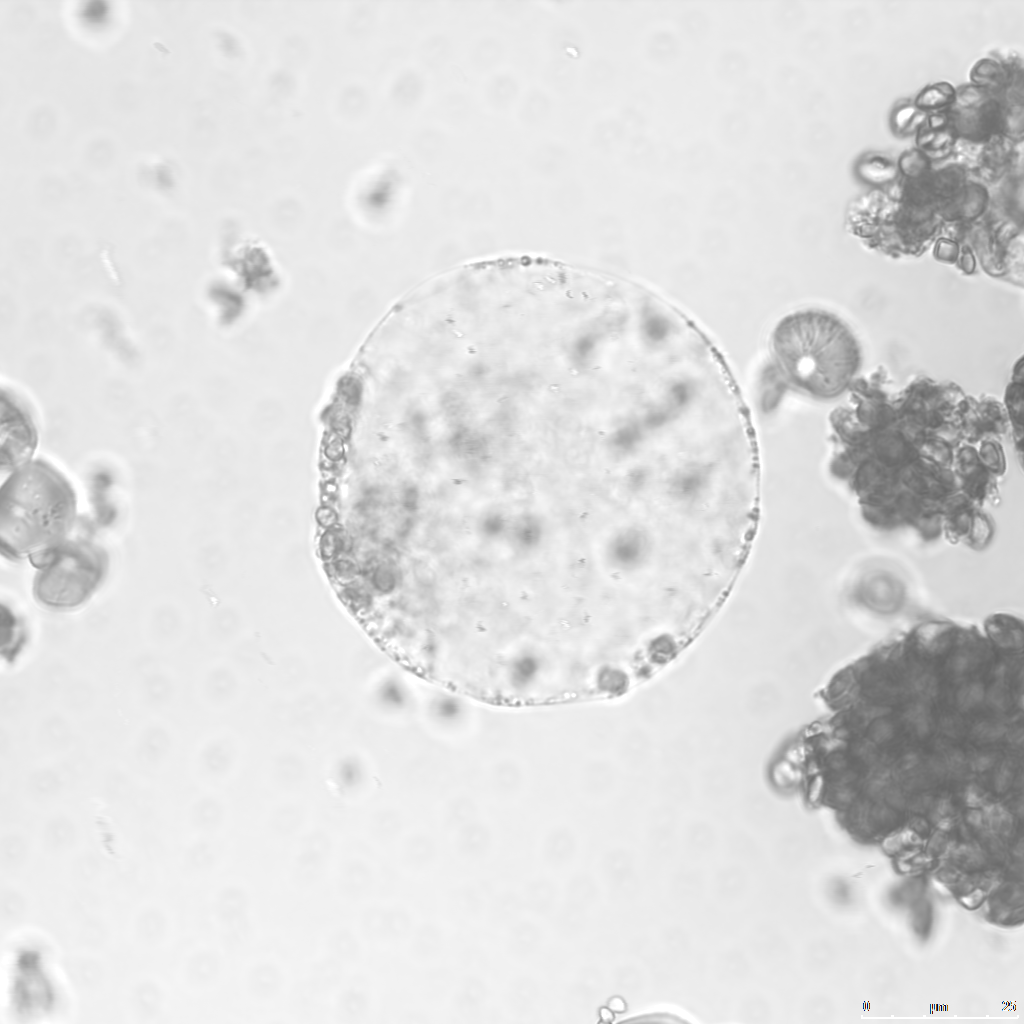

Supplement: Supplemental Information 4 [file peerj-12-16982-s004.zip › Fig 2-Subcellular localization/Protoplast bright 35s-eGFP.tif]

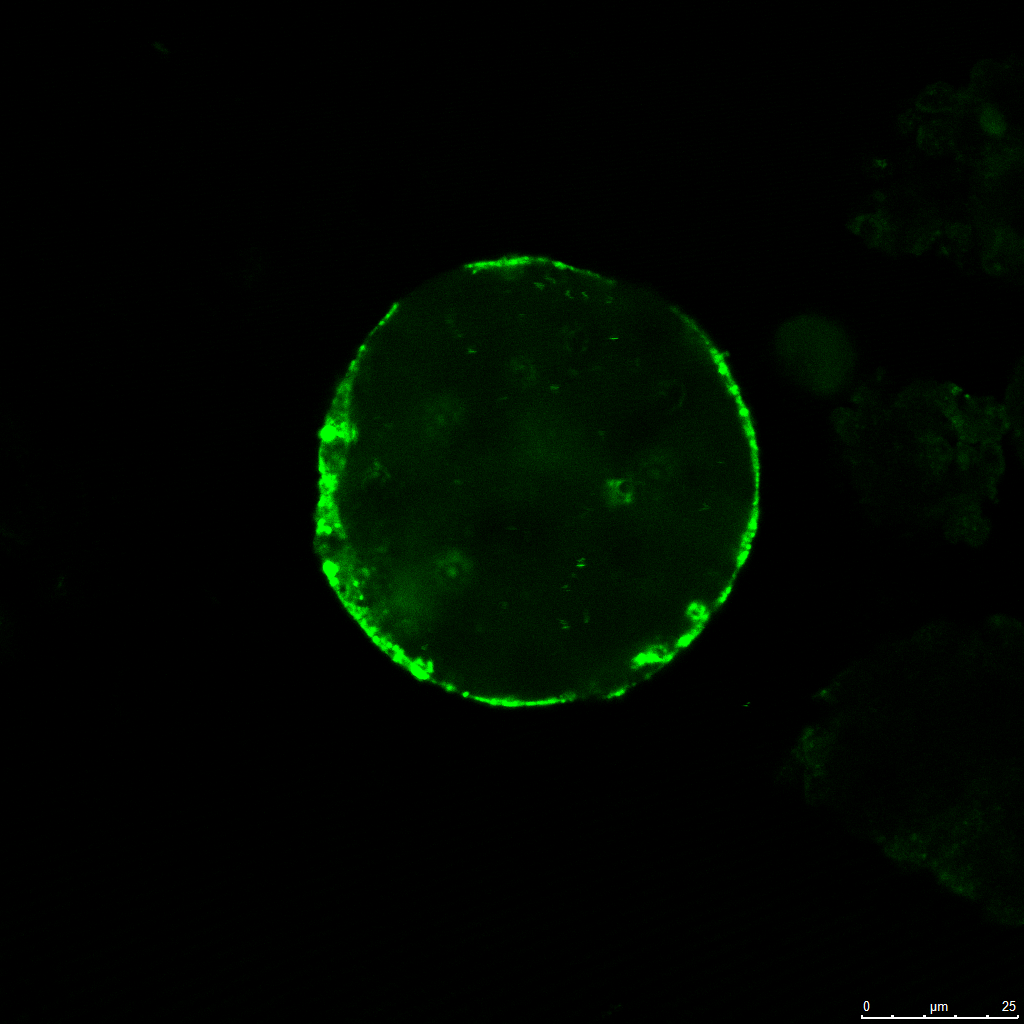

Supplement: Supplemental Information 4 [file peerj-12-16982-s004.zip › Fig 2-Subcellular localization/Protoplast GFP 35s-eGFP.tif]

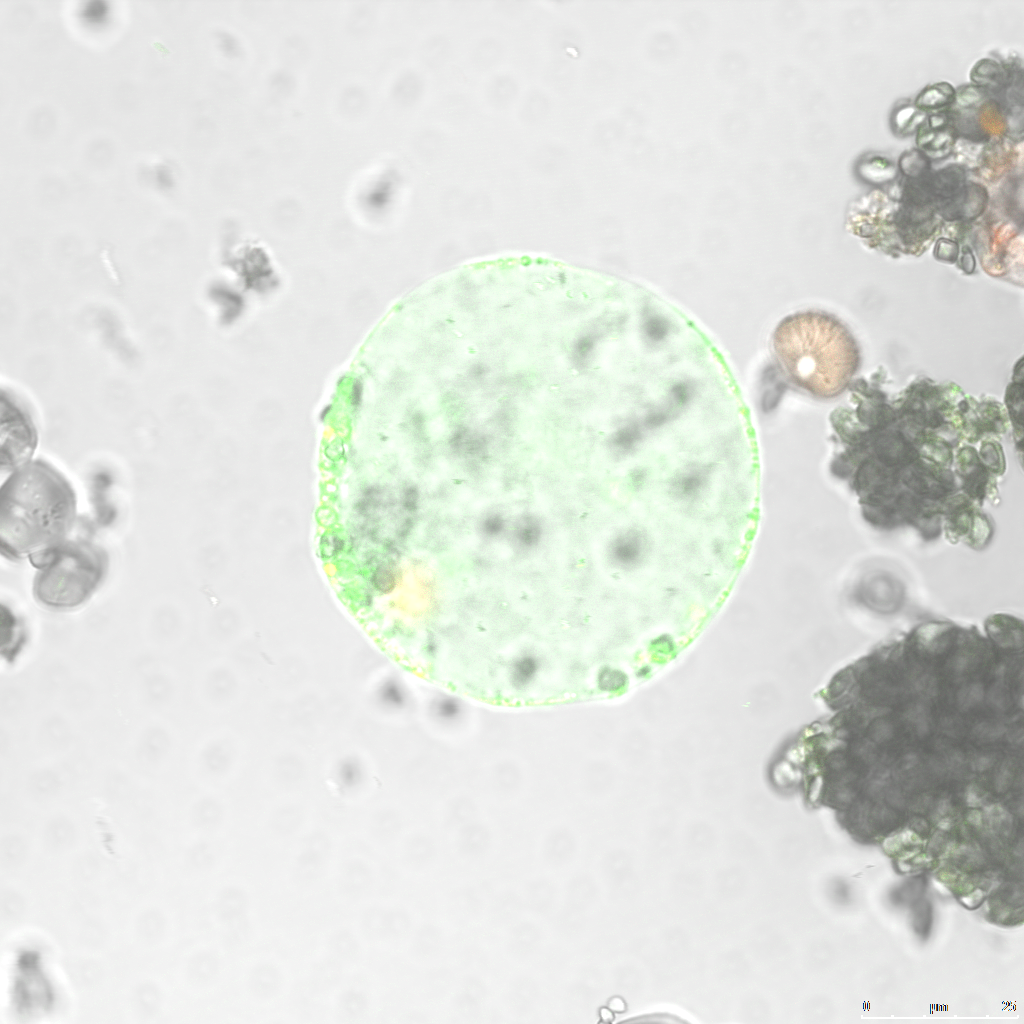

Supplement: Supplemental Information 4 [file peerj-12-16982-s004.zip › Fig 2-Subcellular localization/Protoplast merge 35s-eGFP.tif]

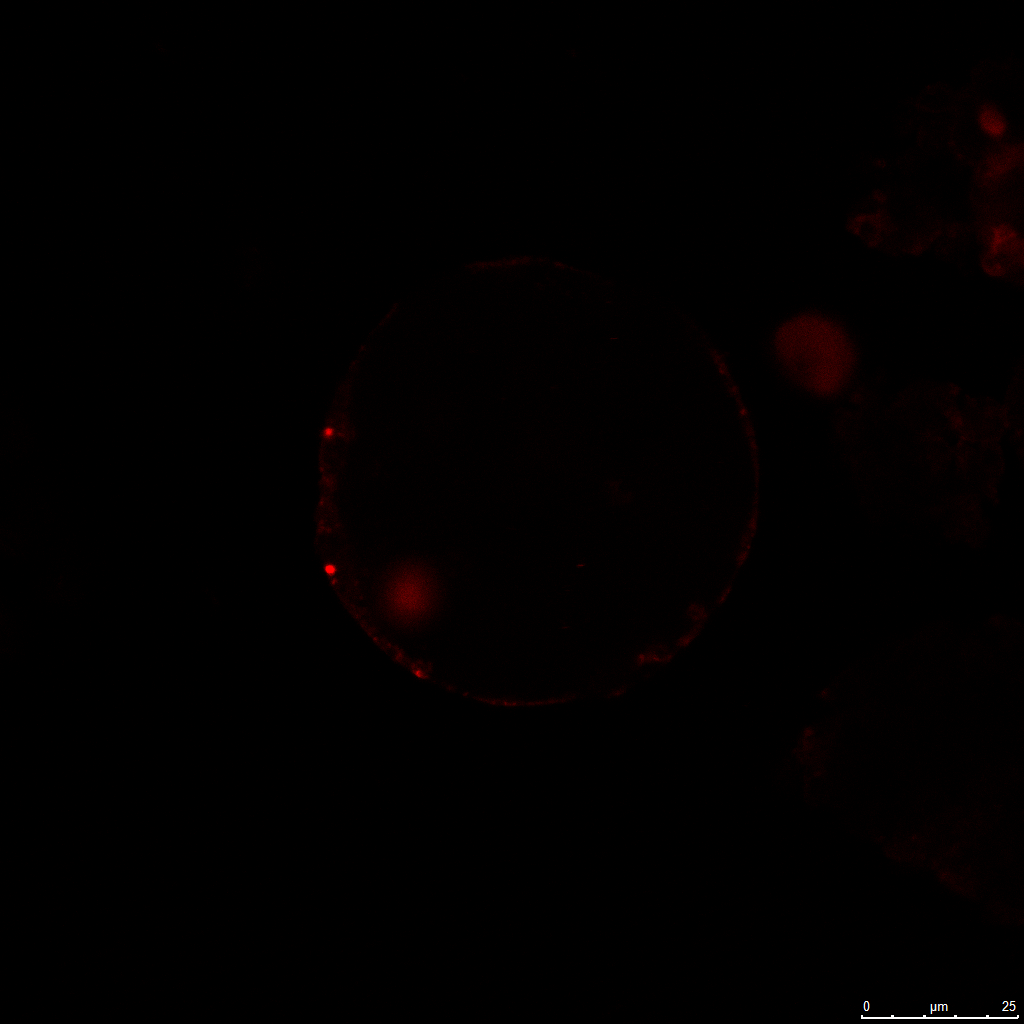

Supplement: Supplemental Information 4 [file peerj-12-16982-s004.zip › Fig 2-Subcellular localization/Protoplast RFP 35s-eGFP.tif]

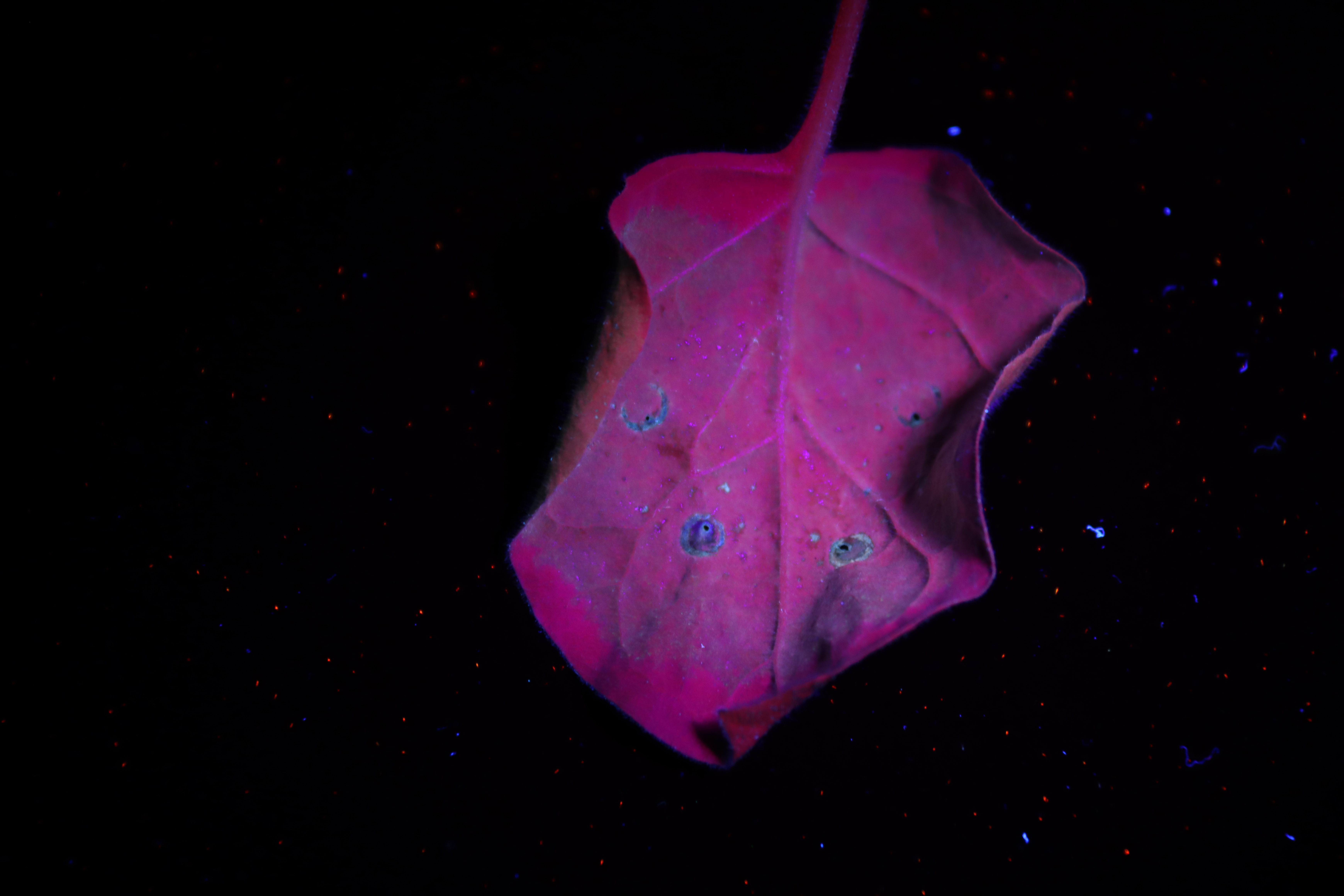

Supplement: Supplemental Information 6 [file peerj-12-16982-s006.zip › Fig 3-SCBV P2 IR-PTGS suppressor activity analysis/Nb P19 trial 2.JPG]

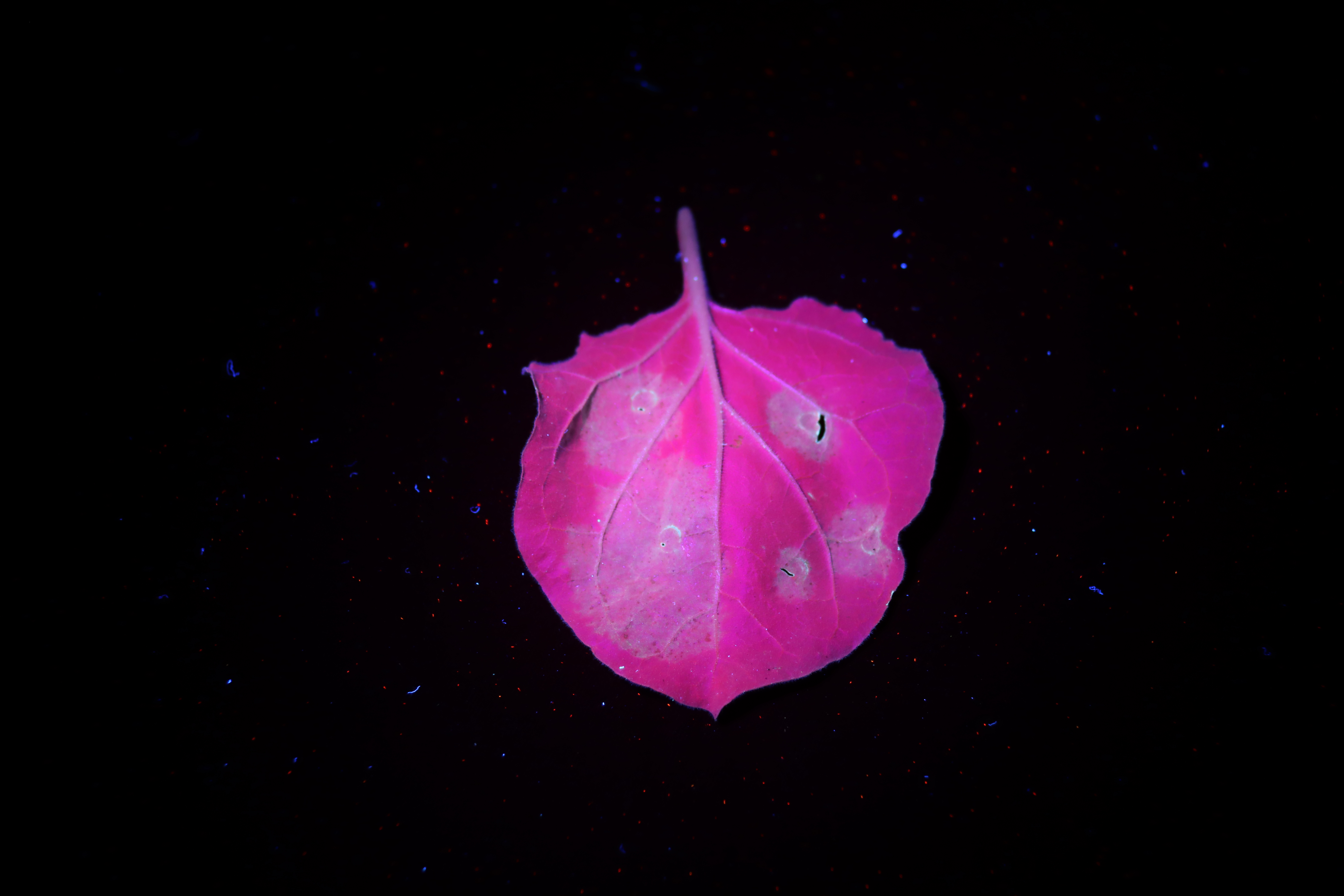

Supplement: Supplemental Information 6 [file peerj-12-16982-s006.zip › Fig 3-SCBV P2 IR-PTGS suppressor activity analysis/Nb P19 trial 3.JPG]

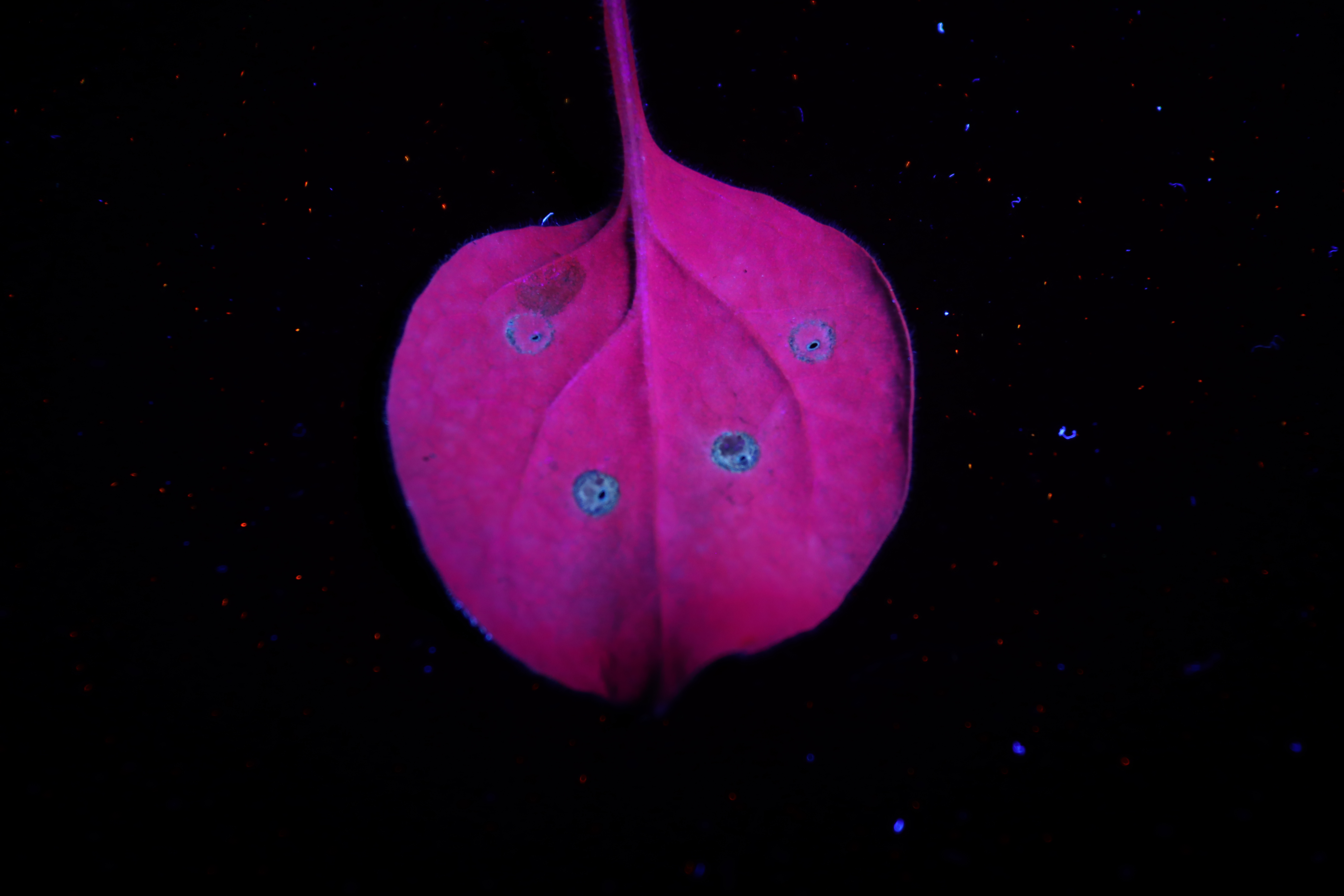

Supplement: Supplemental Information 6 [file peerj-12-16982-s006.zip › Fig 3-SCBV P2 IR-PTGS suppressor activity analysis/Nb pCHF3 trial 1.JPG]

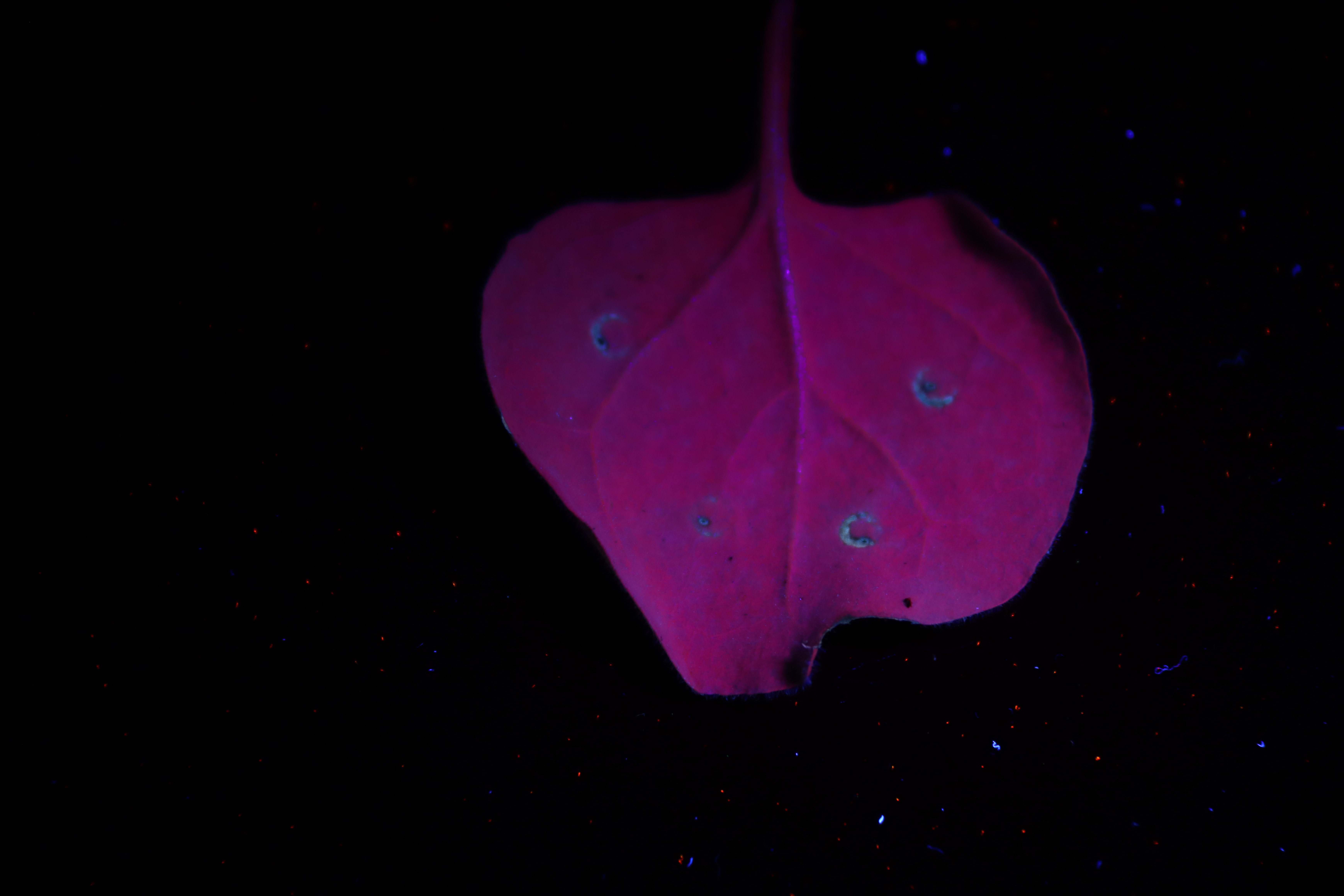

Supplement: Supplemental Information 6 [file peerj-12-16982-s006.zip › Fig 3-SCBV P2 IR-PTGS suppressor activity analysis/Nb pCHF3 trial 2.JPG]

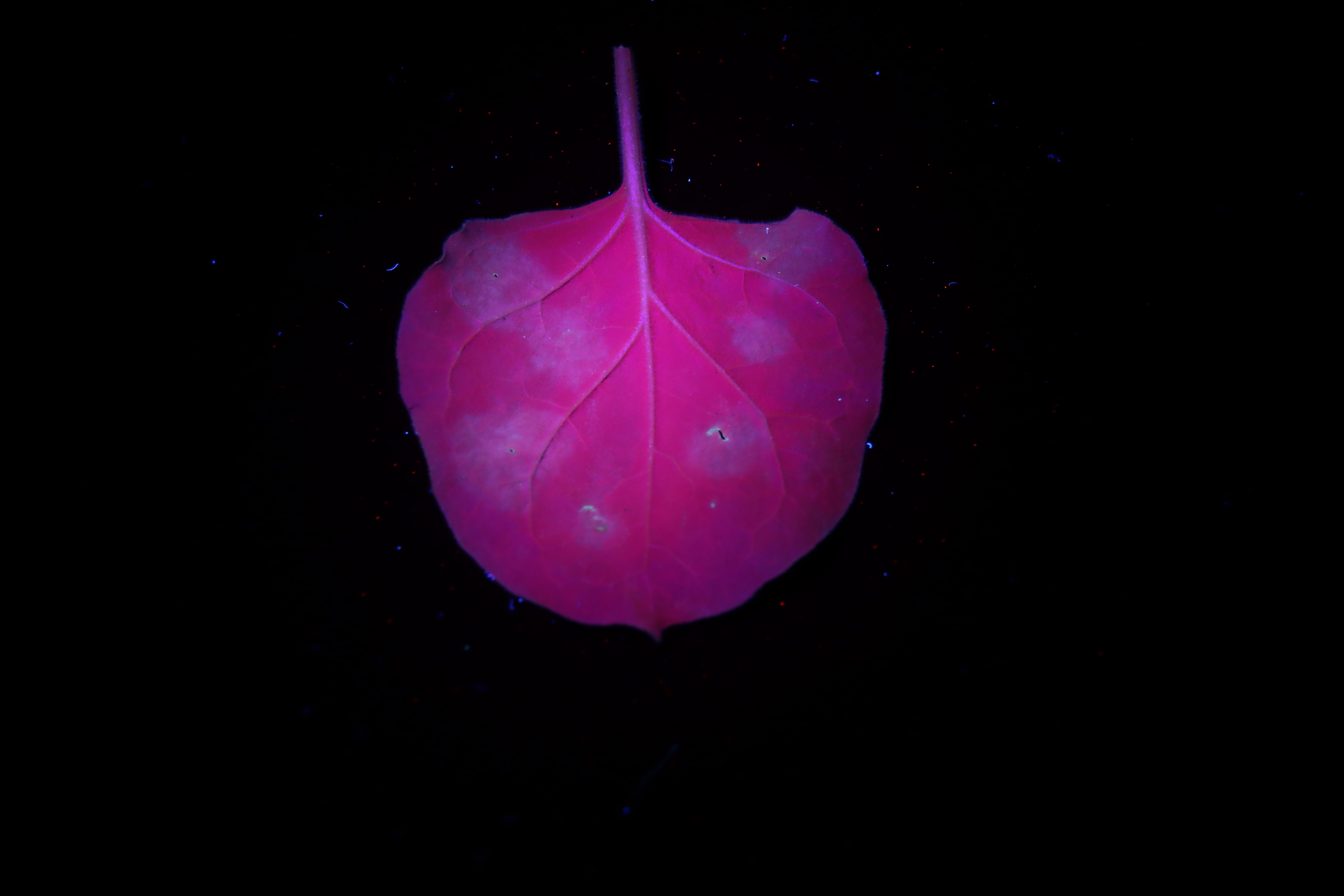

Supplement: Supplemental Information 6 [file peerj-12-16982-s006.zip › Fig 3-SCBV P2 IR-PTGS suppressor activity analysis/Nb pCHF3 trial 3.JPG]

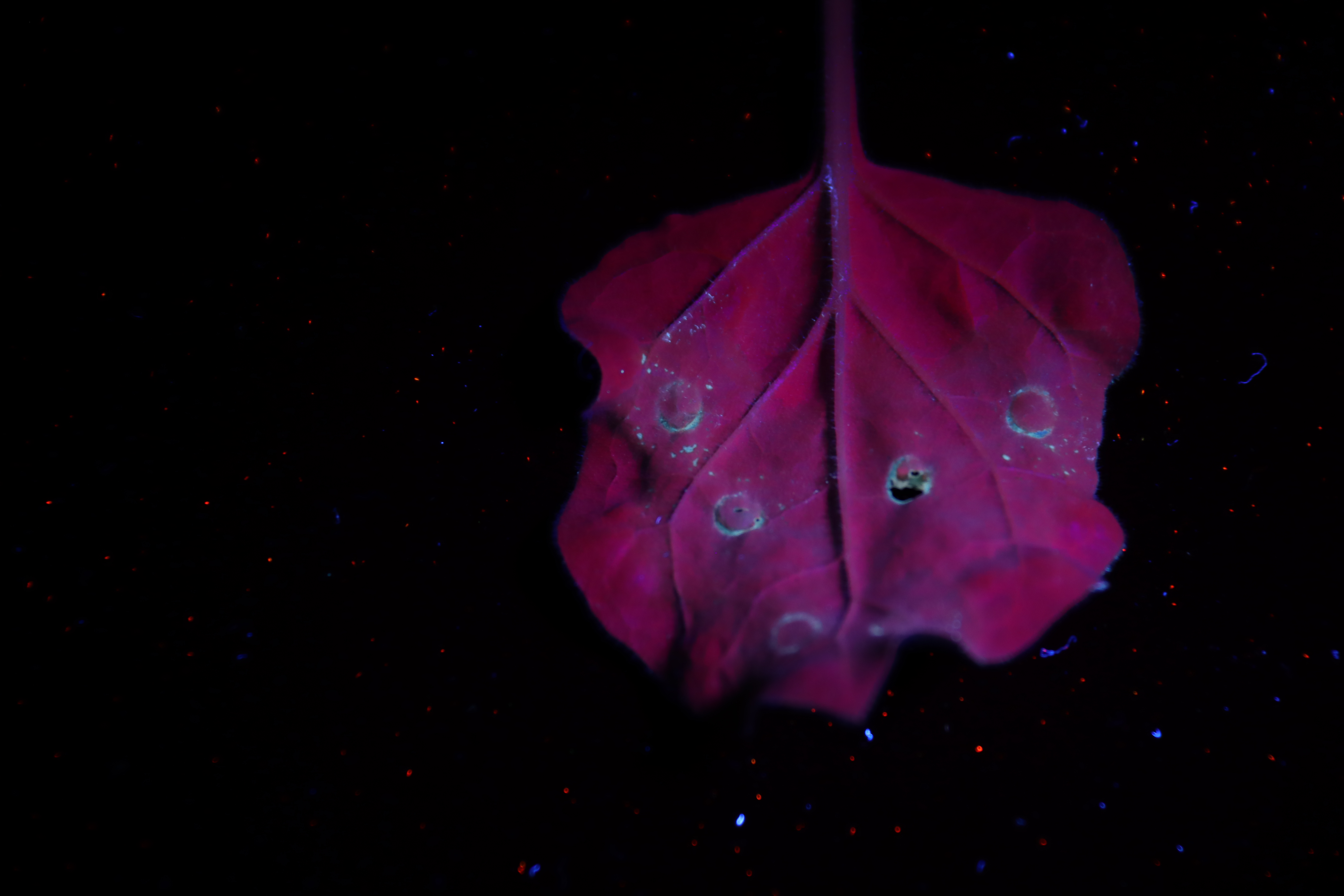

Supplement: Supplemental Information 6 [file peerj-12-16982-s006.zip › Fig 3-SCBV P2 IR-PTGS suppressor activity analysis/Nb SCBV-P2 trial 1.JPG]

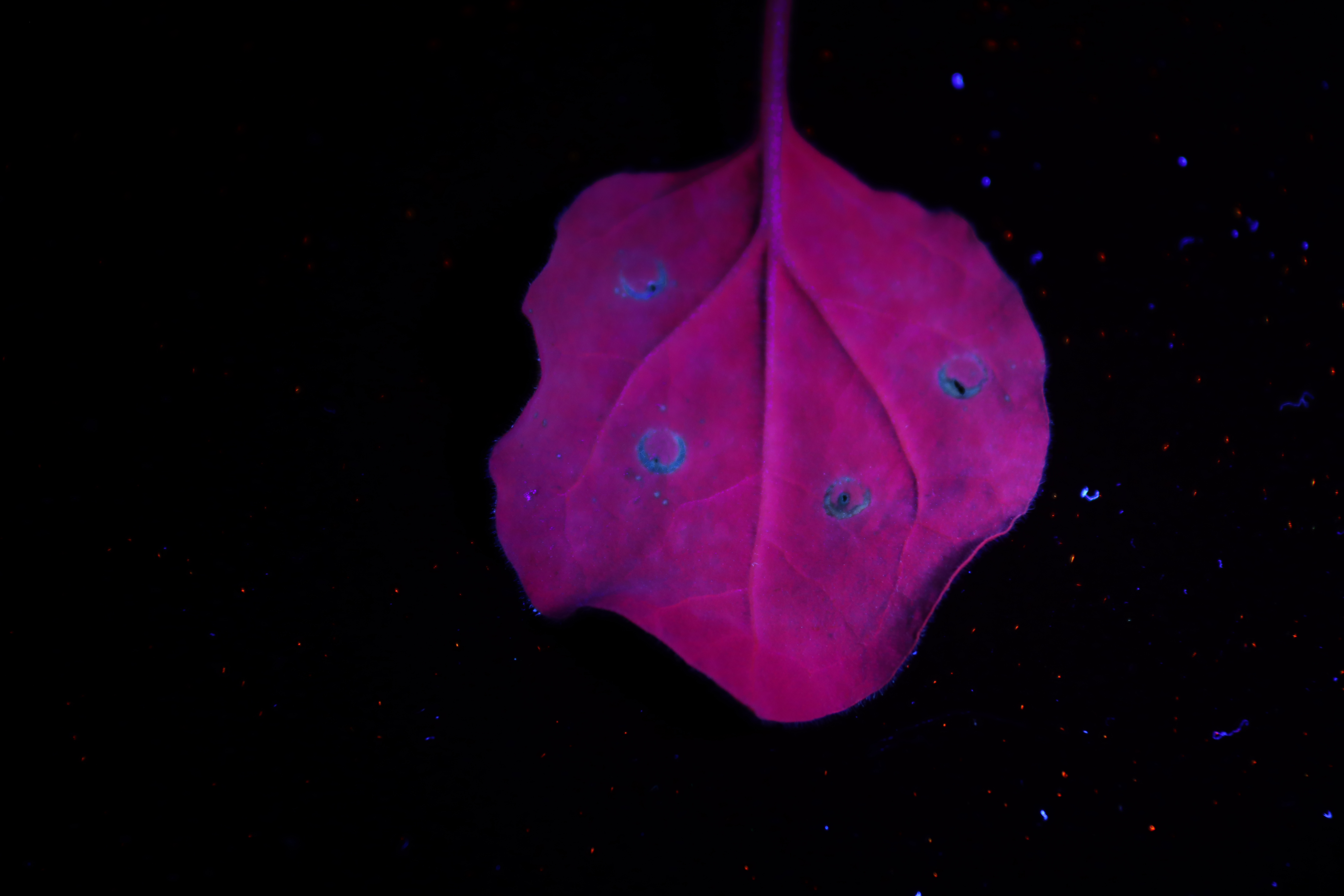

Supplement: Supplemental Information 6 [file peerj-12-16982-s006.zip › Fig 3-SCBV P2 IR-PTGS suppressor activity analysis/Nb SCBV-P2 trial 2.JPG]

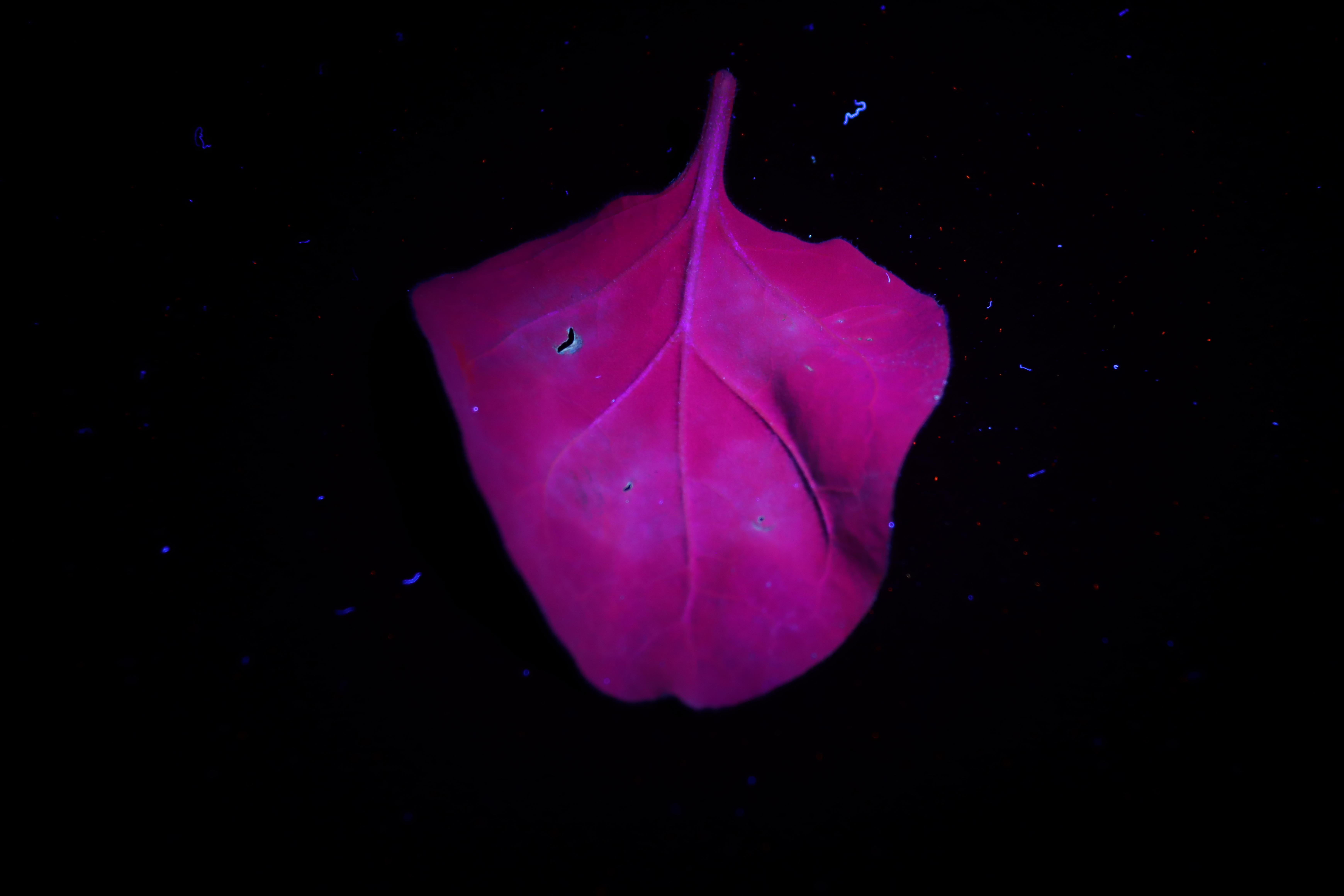

Supplement: Supplemental Information 6 [file peerj-12-16982-s006.zip › Fig 3-SCBV P2 IR-PTGS suppressor activity analysis/Nb SCBV-P2 trial 3.JPG]

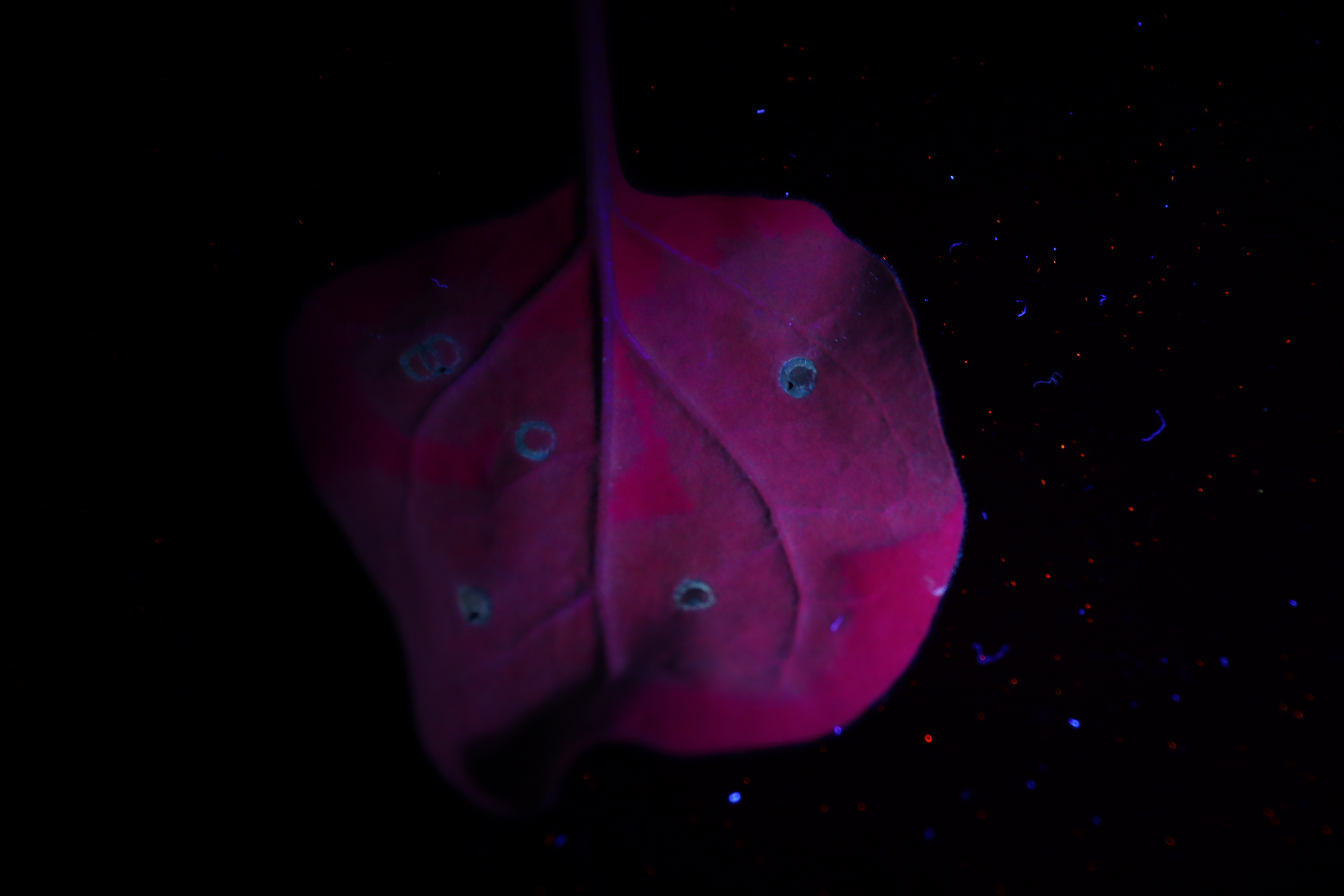

Supplement: Supplemental Information 6 [file peerj-12-16982-s006.zip › Fig 3-SCBV P2 IR-PTGS suppressor activity analysis/Nb P19 trial 1.JPG]

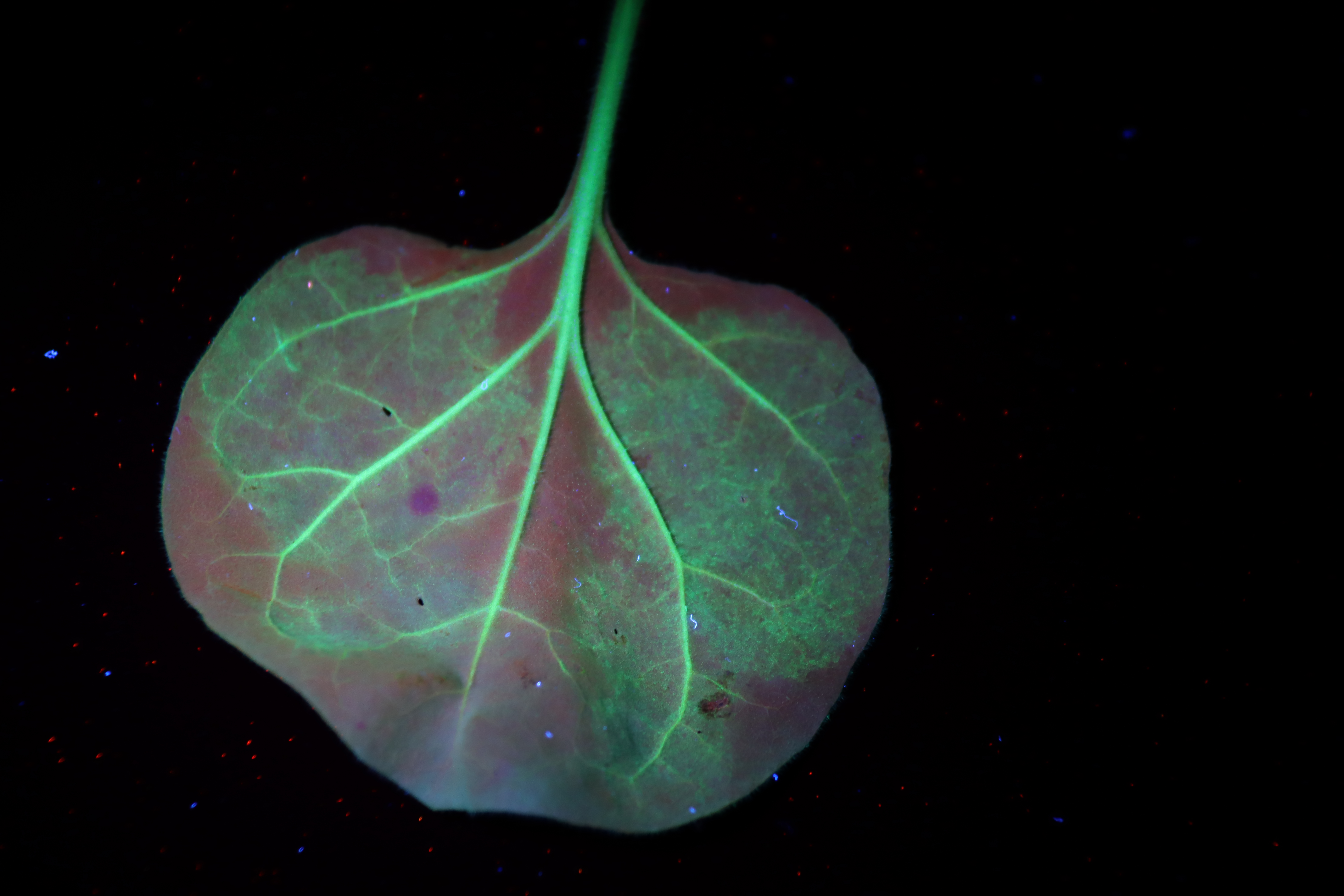

Supplement: Supplemental Information 7 [file peerj-12-16982-s007.zip › Fig 3-SCBV P2 ss-PTGS suppressor activity analysis/16c P19 trial 3.JPG]

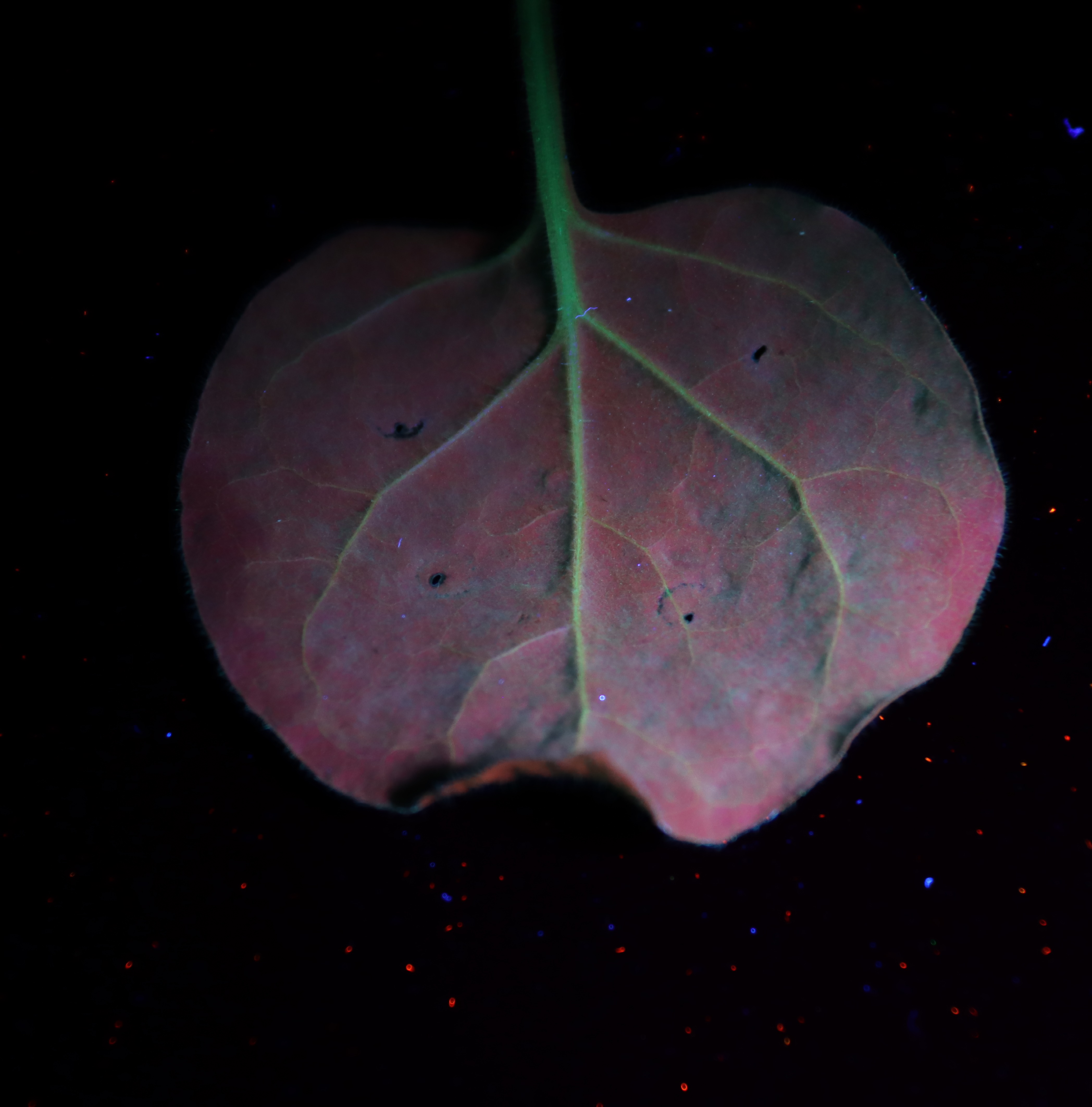

Supplement: Supplemental Information 7 [file peerj-12-16982-s007.zip › Fig 3-SCBV P2 ss-PTGS suppressor activity analysis/16c pCHF3 trial 1.jpg]

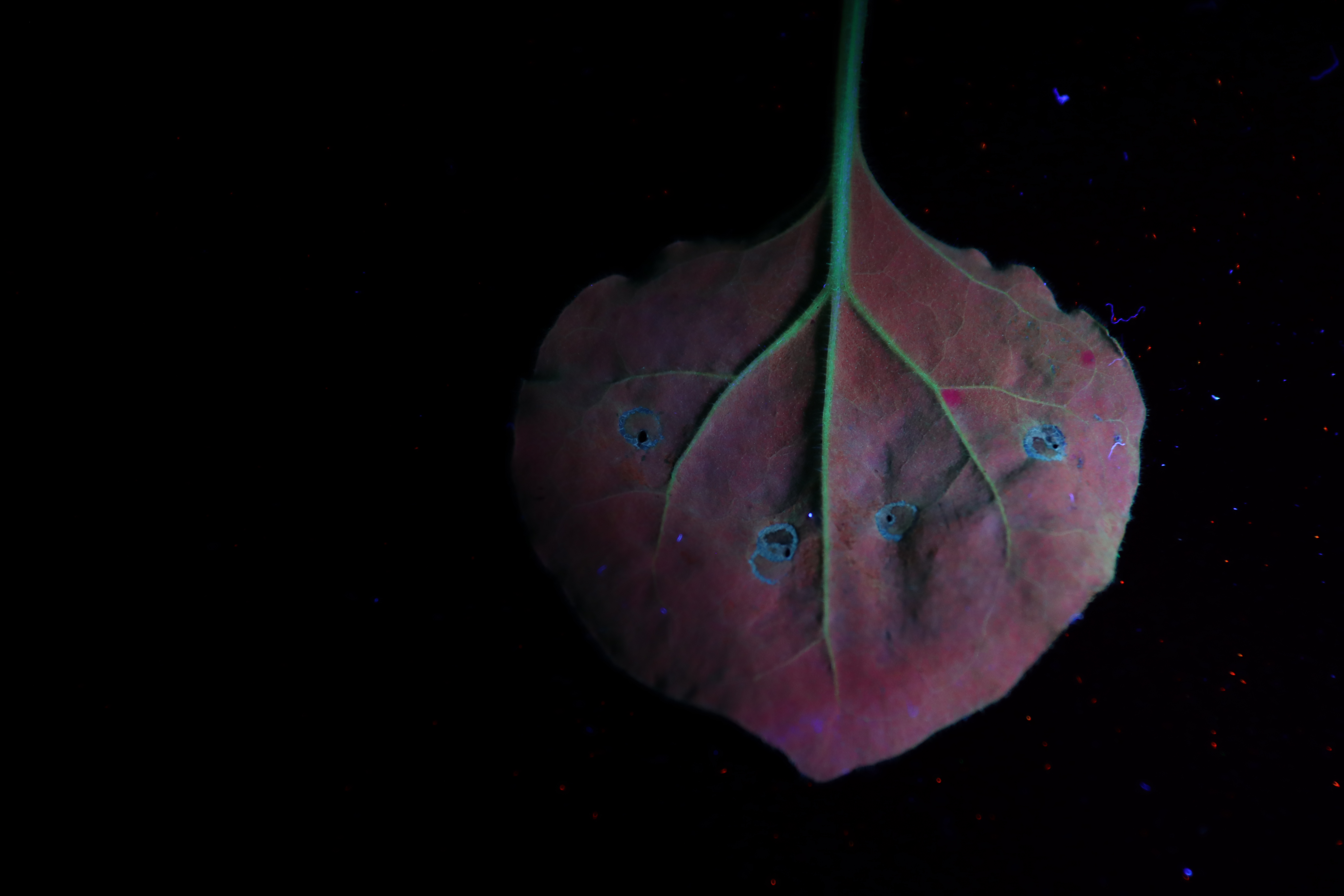

Supplement: Supplemental Information 7 [file peerj-12-16982-s007.zip › Fig 3-SCBV P2 ss-PTGS suppressor activity analysis/16c pCHF3 trial 2.JPG]

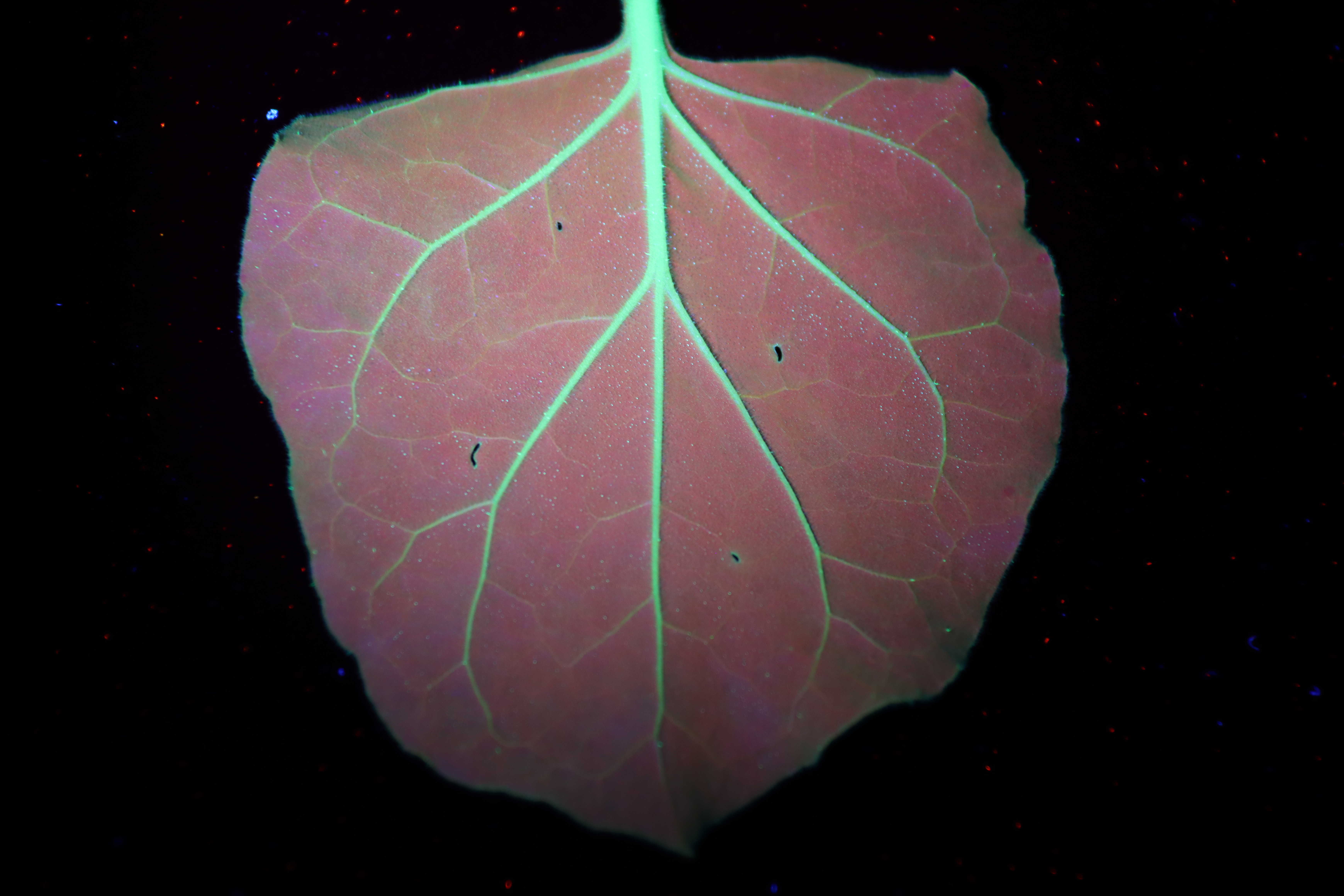

Supplement: Supplemental Information 7 [file peerj-12-16982-s007.zip › Fig 3-SCBV P2 ss-PTGS suppressor activity analysis/16c pCHF3 trial 3.JPG]

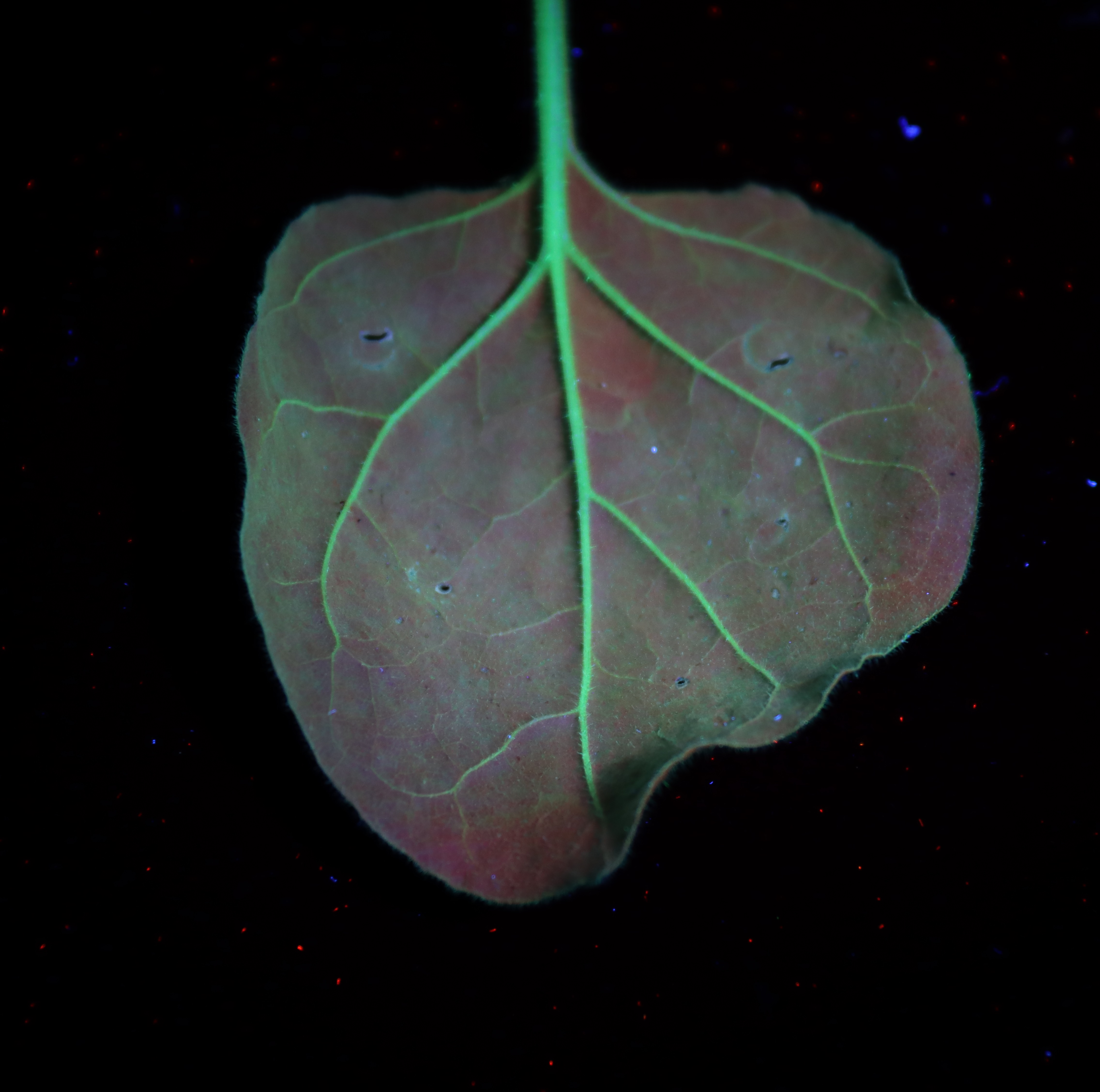

Supplement: Supplemental Information 7 [file peerj-12-16982-s007.zip › Fig 3-SCBV P2 ss-PTGS suppressor activity analysis/16c SCBV-P2 trial 1.jpg]

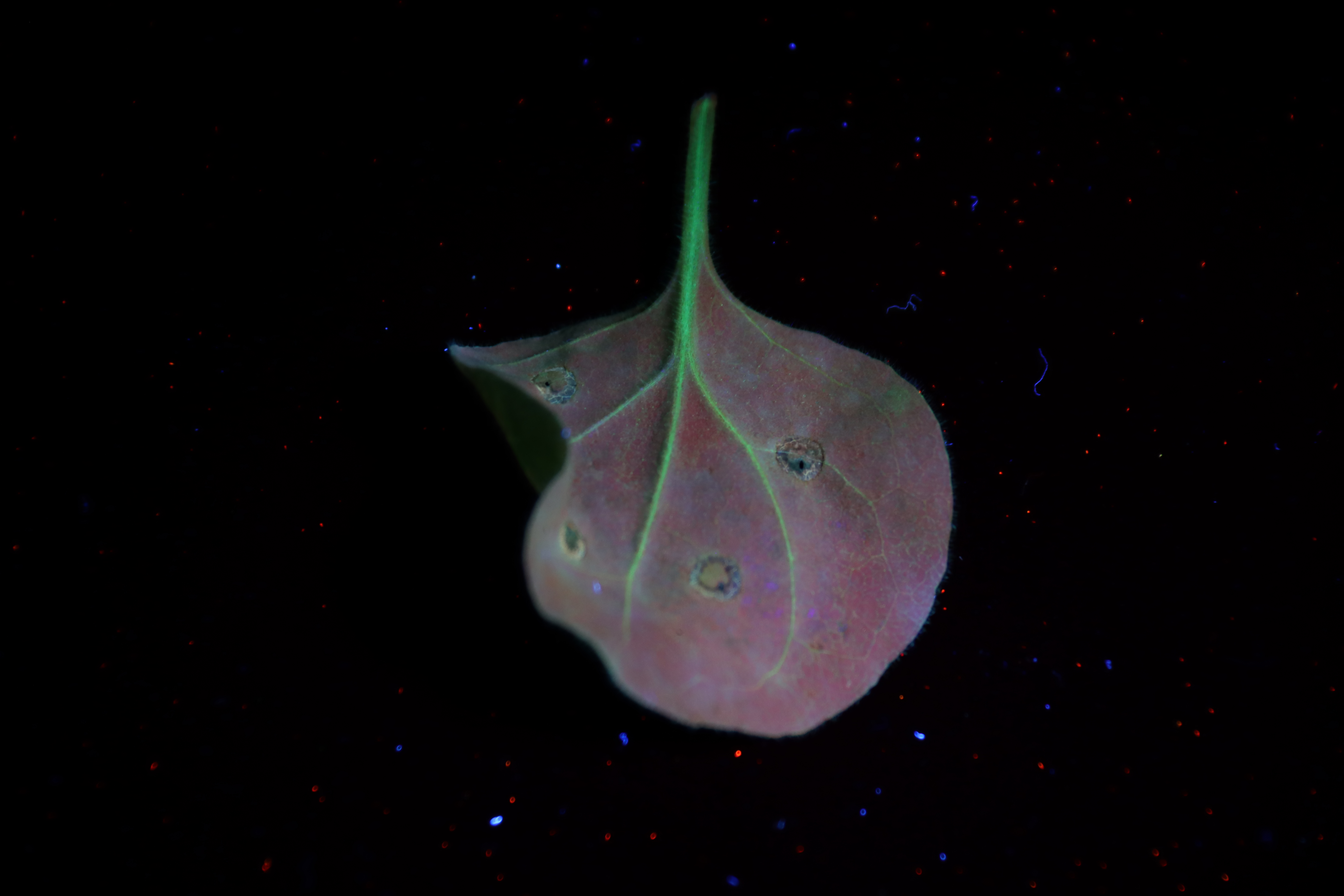

Supplement: Supplemental Information 7 [file peerj-12-16982-s007.zip › Fig 3-SCBV P2 ss-PTGS suppressor activity analysis/16c SCBV-P2 trial 2.JPG]

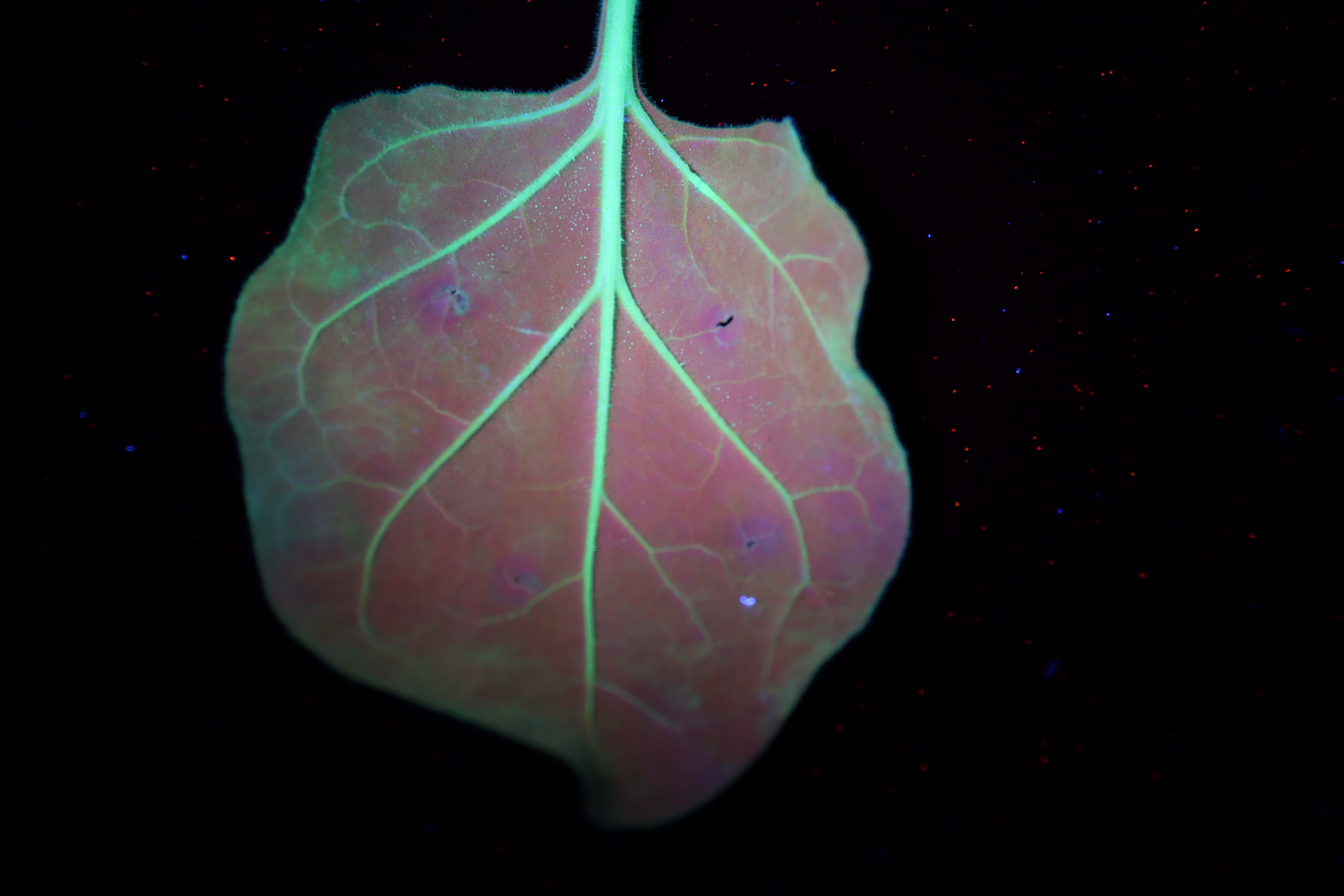

Supplement: Supplemental Information 7 [file peerj-12-16982-s007.zip › Fig 3-SCBV P2 ss-PTGS suppressor activity analysis/16c SCBV-P2 trial 3.JPG]

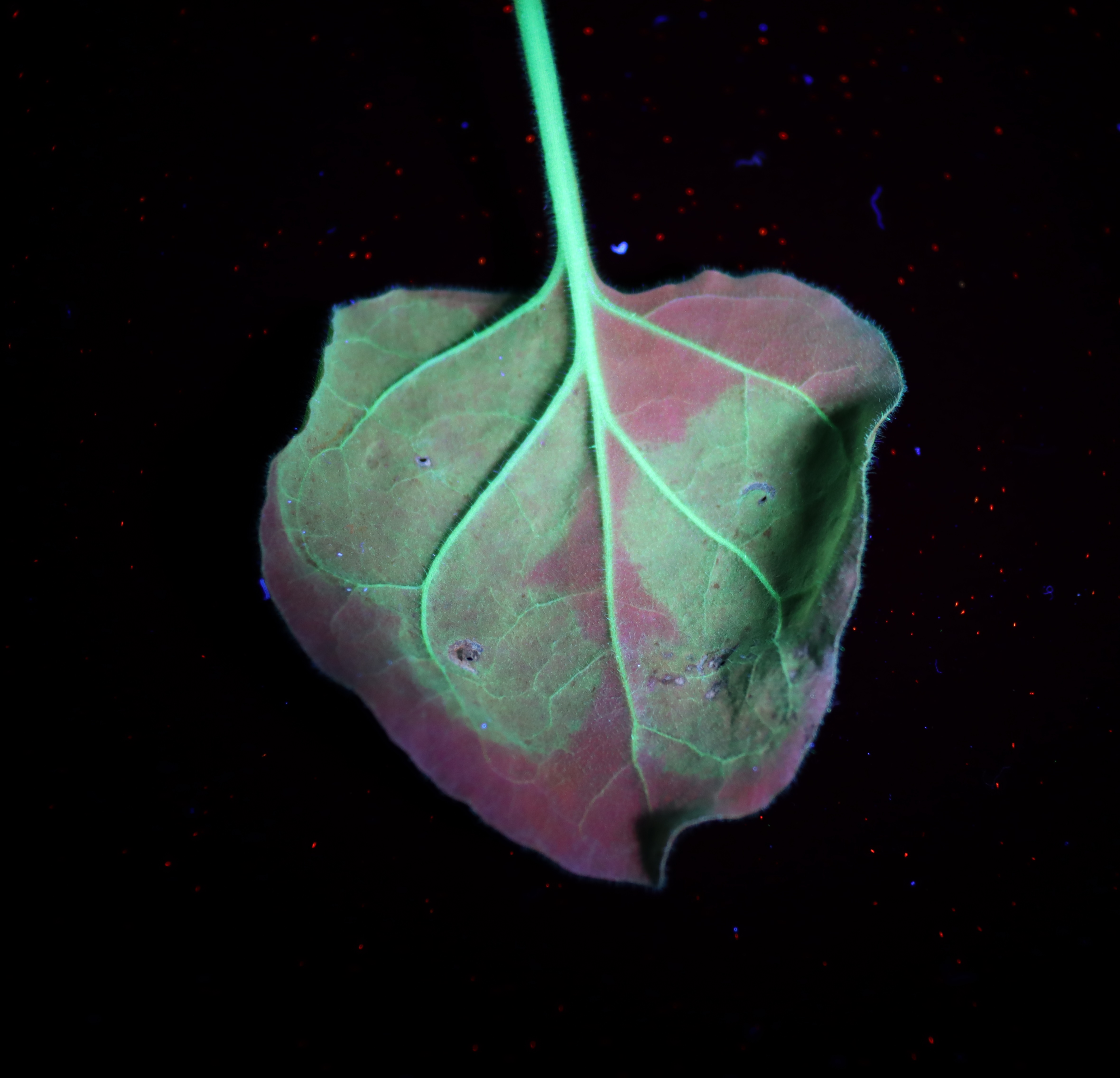

Supplement: Supplemental Information 7 [file peerj-12-16982-s007.zip › Fig 3-SCBV P2 ss-PTGS suppressor activity analysis/16c P19 trial 1.jpg]

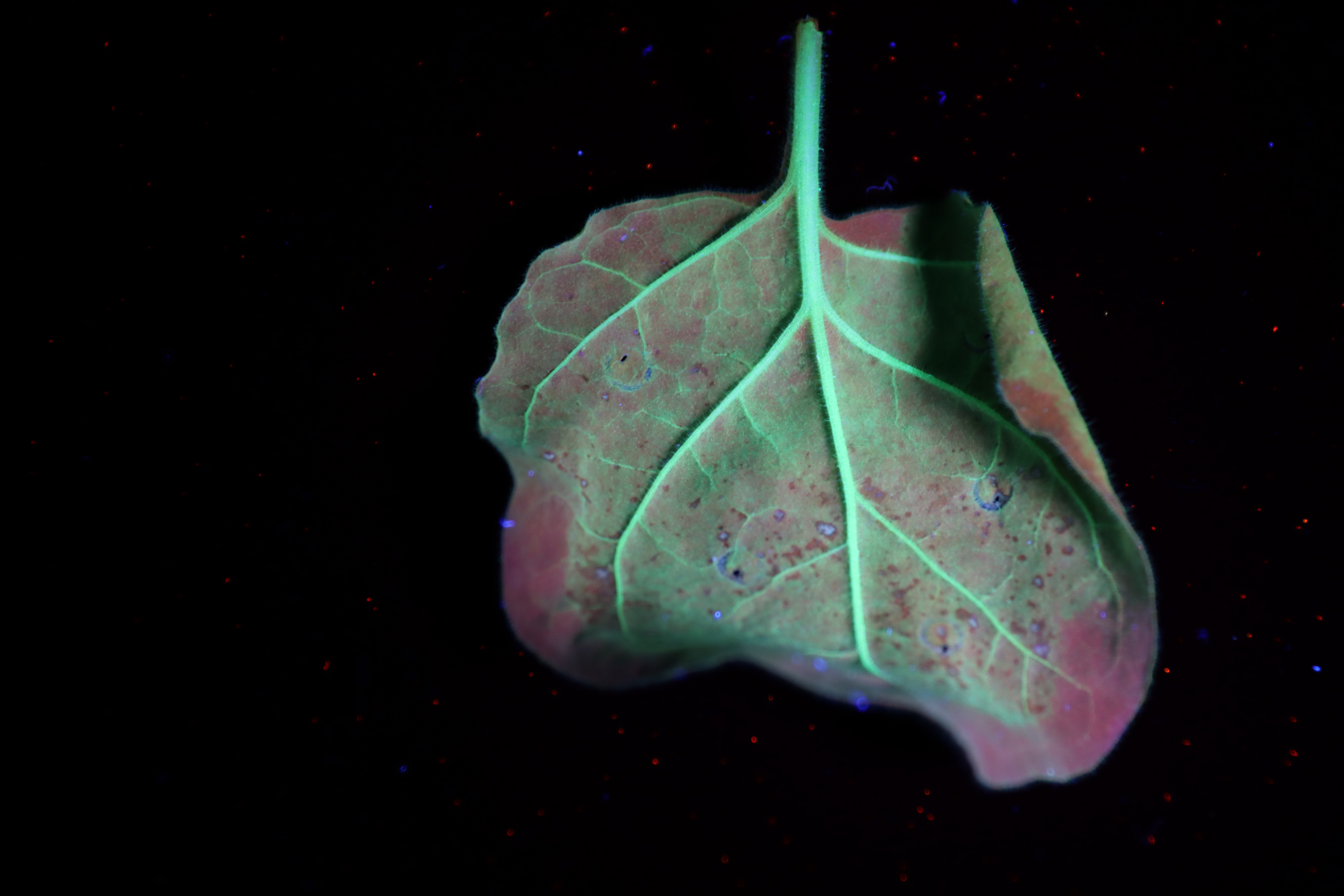

Supplement: Supplemental Information 7 [file peerj-12-16982-s007.zip › Fig 3-SCBV P2 ss-PTGS suppressor activity analysis/16c P19 trial 2.JPG]

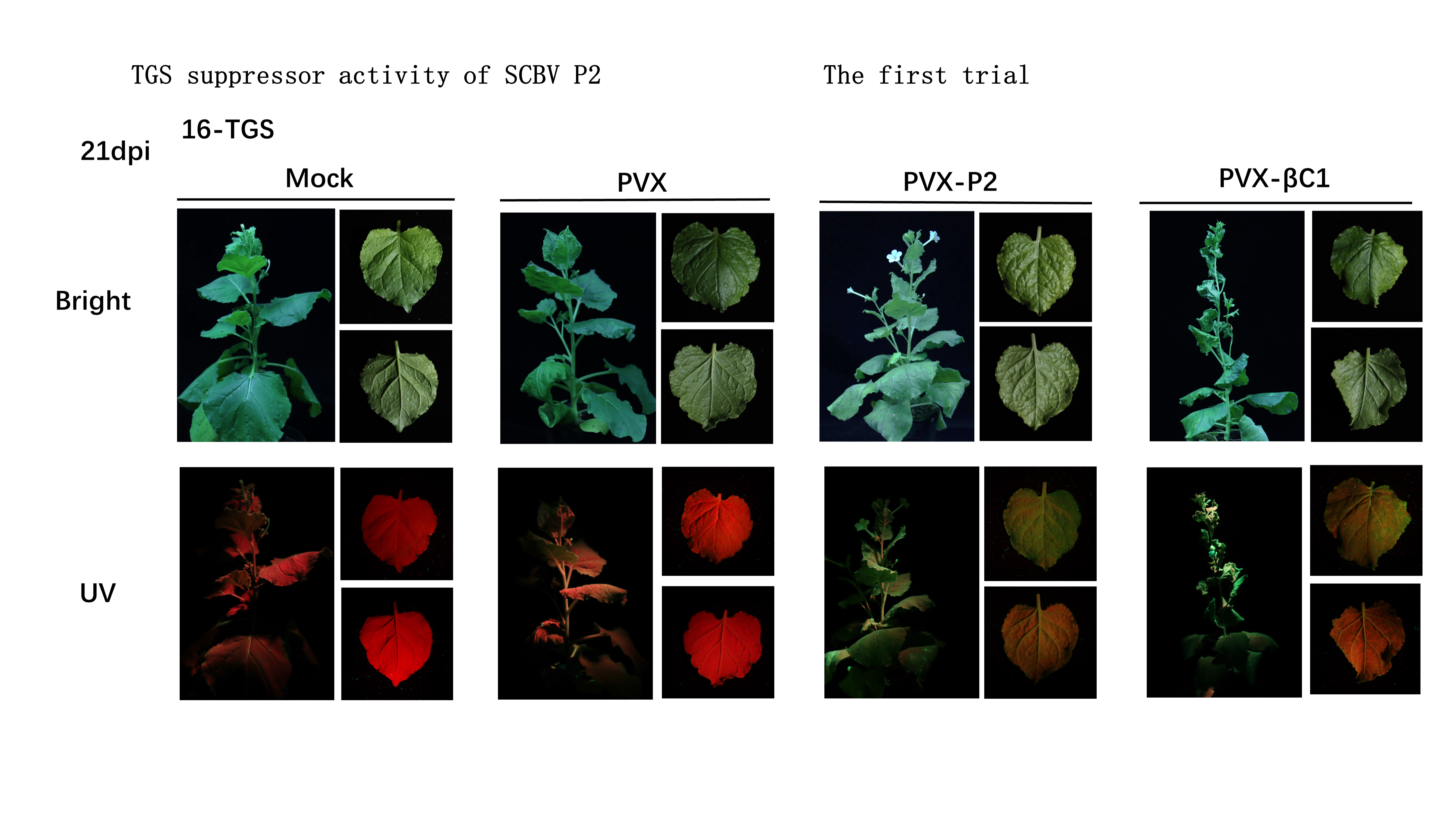

Supplement: Supplemental Information 8 [file peerj-12-16982-s008.zip › Fig 3-P2-TGS suppressor activity analysis/1.png]

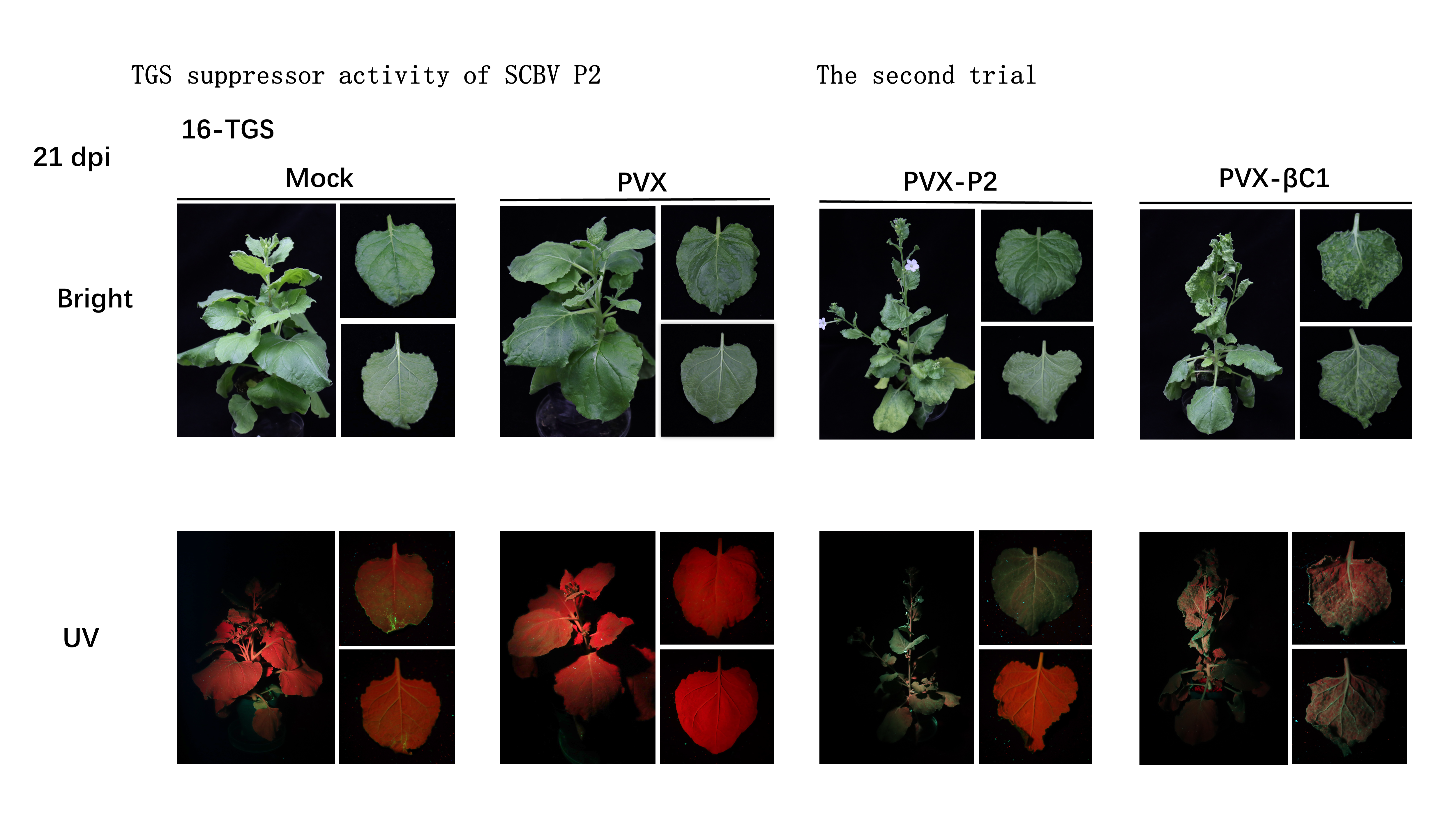

Supplement: Supplemental Information 8 [file peerj-12-16982-s008.zip › Fig 3-P2-TGS suppressor activity analysis/2.png]

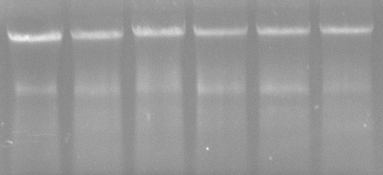

Supplement: Supplemental Information 9 — The uncropped gels of restriction endonuclease digestion assays and raw data of Real-Time Quantitative PCR. [file peerj-12-16982-s009.zip › SCBV-P2 Transgene methylation detection-Sham.png]

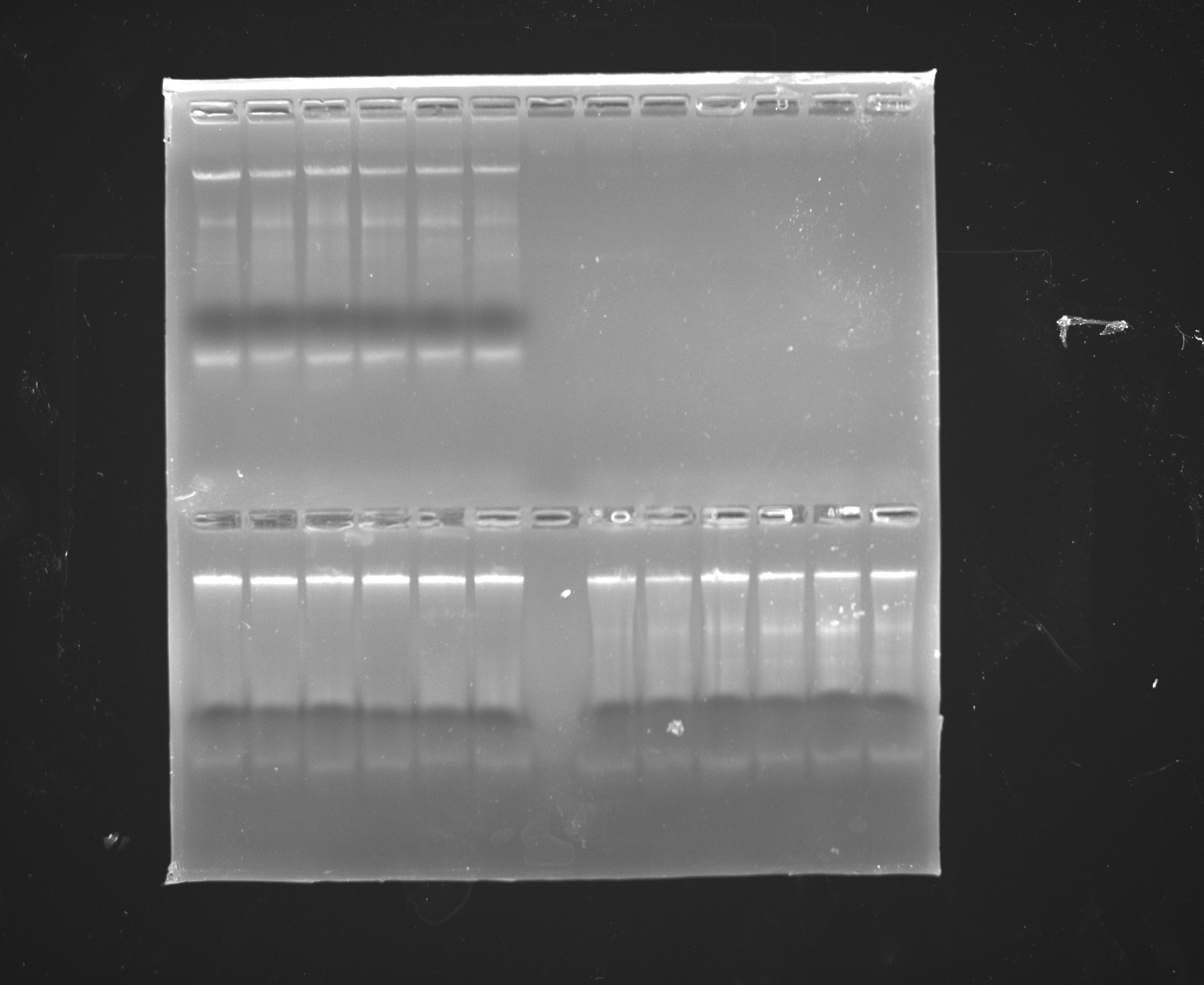

Supplement: Supplemental Information 9 — The uncropped gels of restriction endonuclease digestion assays and raw data of Real-Time Quantitative PCR. [file peerj-12-16982-s009.zip › SCBV-P2 Transgene methylation detection by endonuclease digestion.tif]

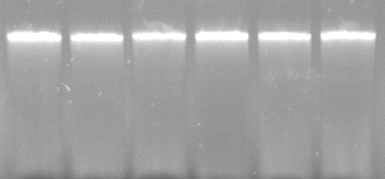

Supplement: Supplemental Information 9 — The uncropped gels of restriction endonuclease digestion assays and raw data of Real-Time Quantitative PCR. [file peerj-12-16982-s009.zip › SCBV-P2 Transgene methylation detection-BamHI.png]

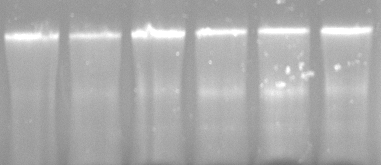

Supplement: Supplemental Information 9 — The uncropped gels of restriction endonuclease digestion assays and raw data of Real-Time Quantitative PCR. [file peerj-12-16982-s009.zip › SCBV-P2 Transgene methylation detection-McrBC.png]

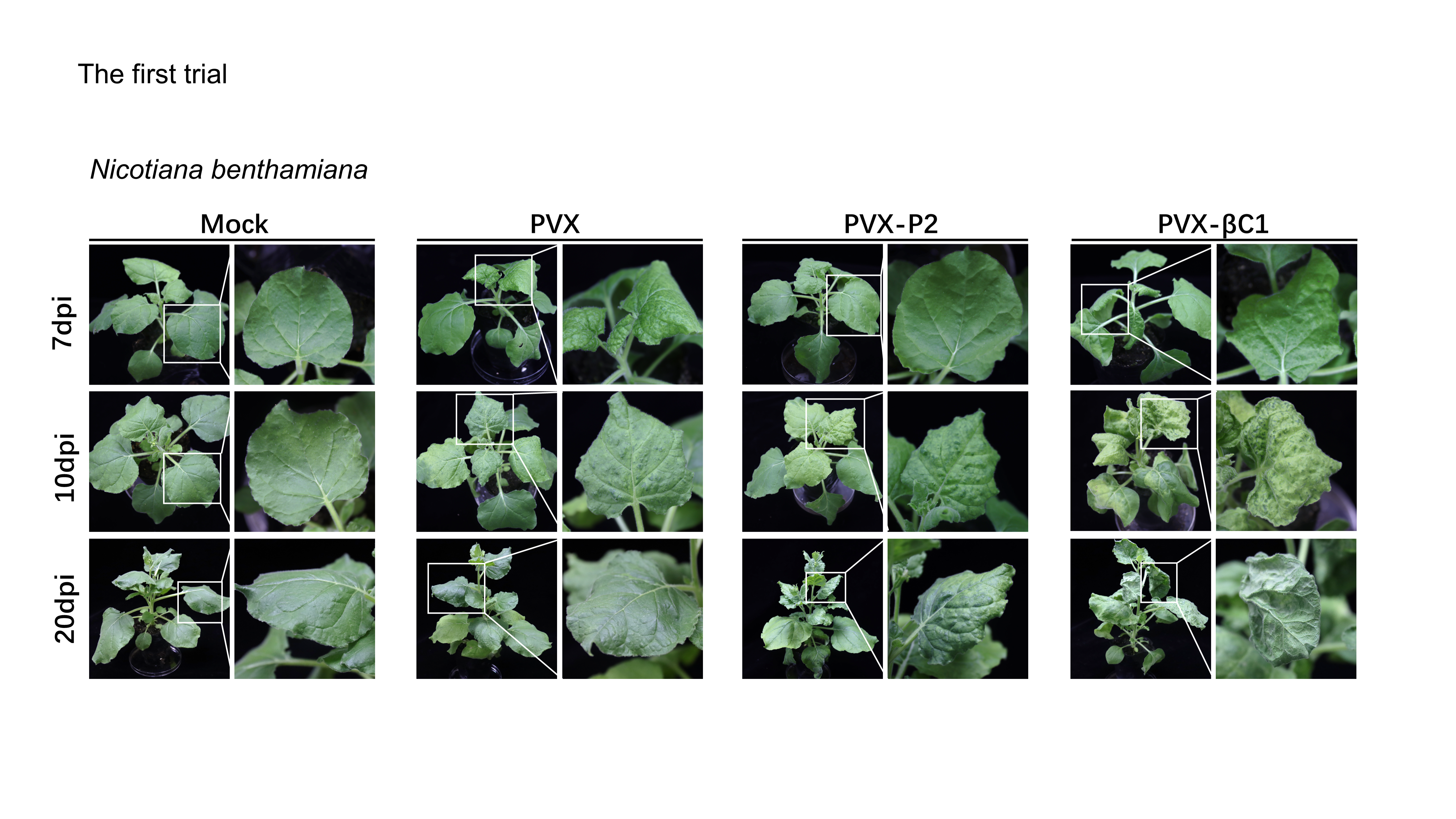

Supplement: Supplemental Information 10 [file peerj-12-16982-s010.png]

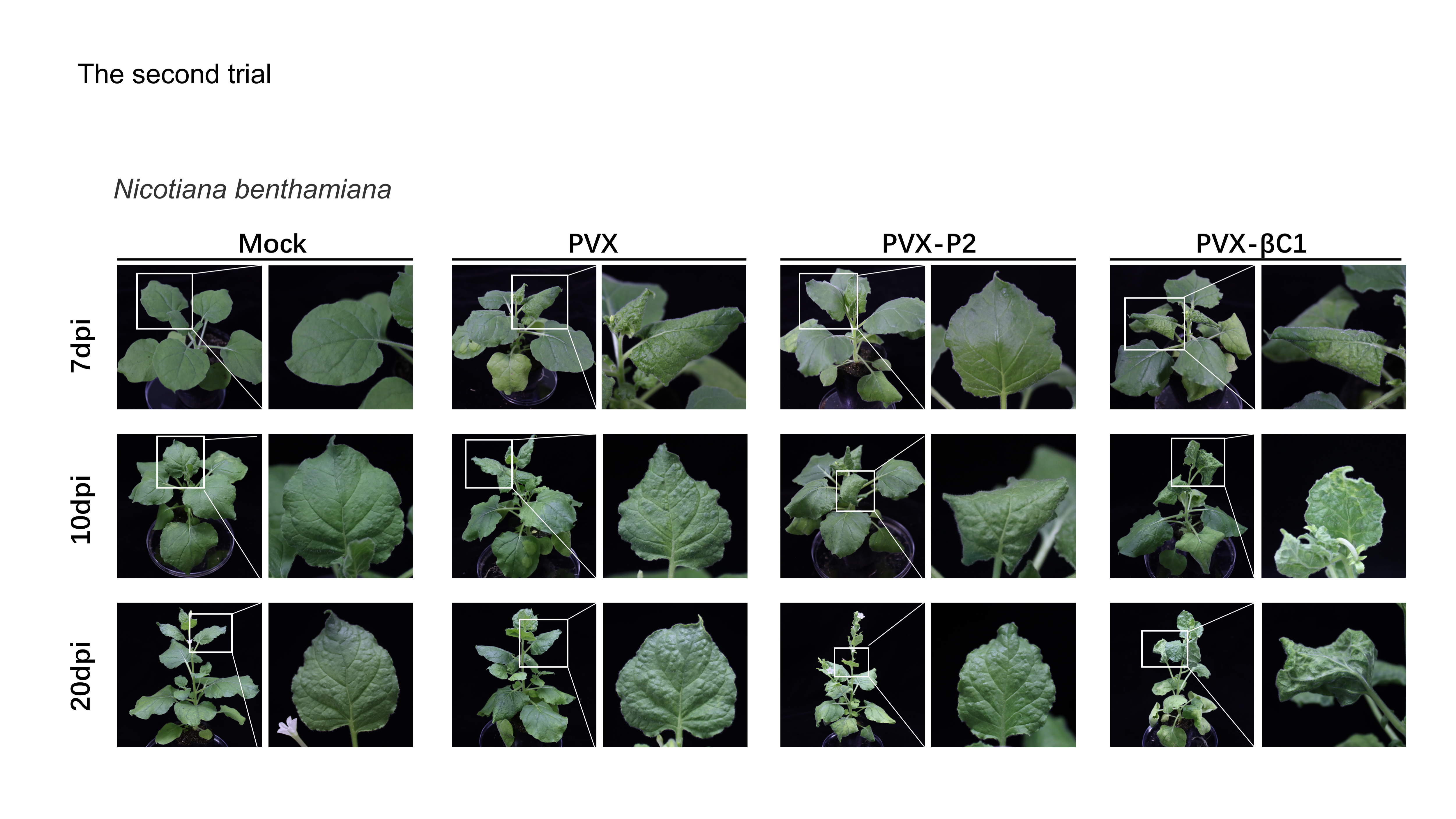

Supplement: Supplemental Information 11 [file peerj-12-16982-s011.png]

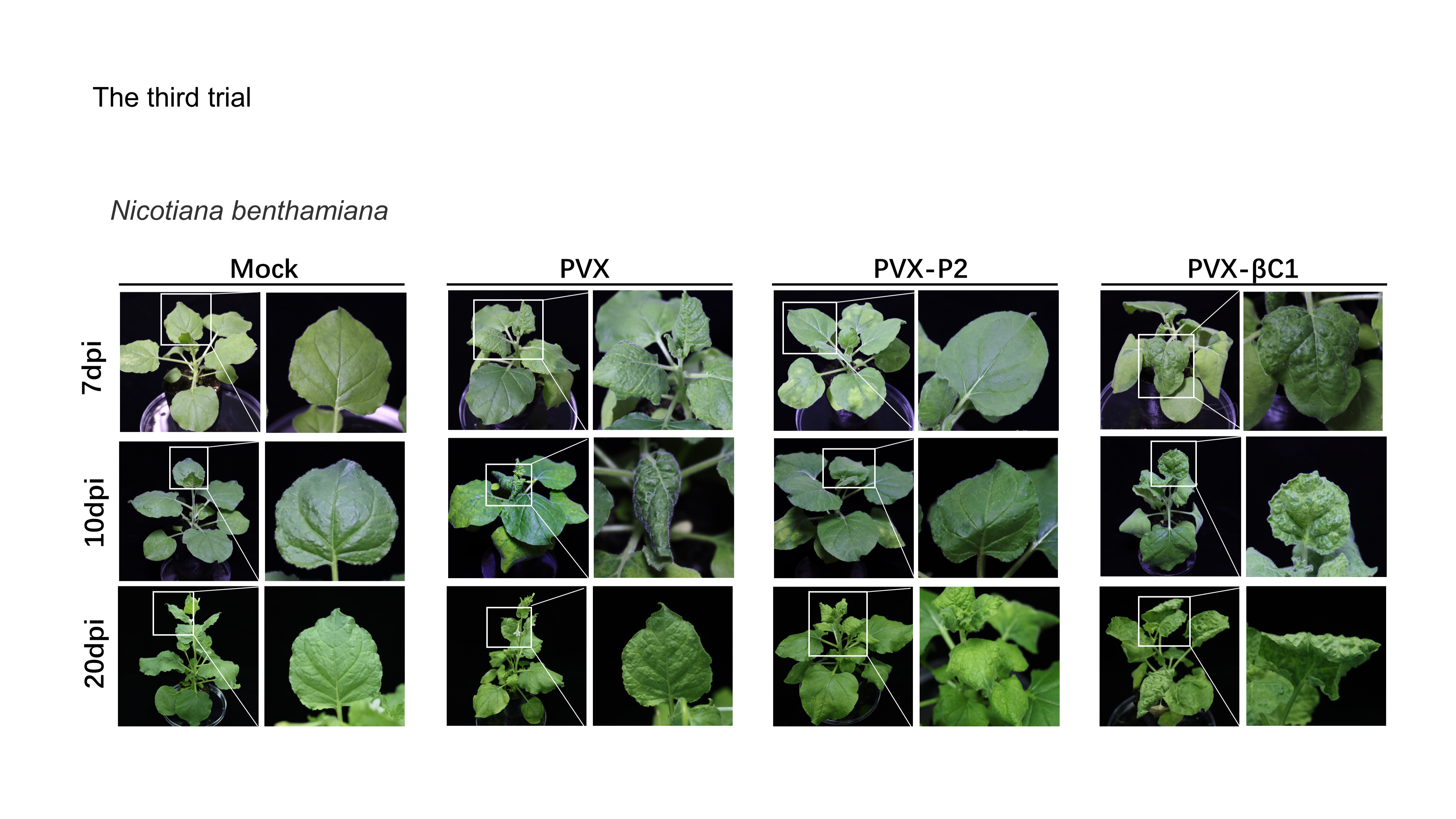

Supplement: Supplemental Information 12 [file peerj-12-16982-s012.png]

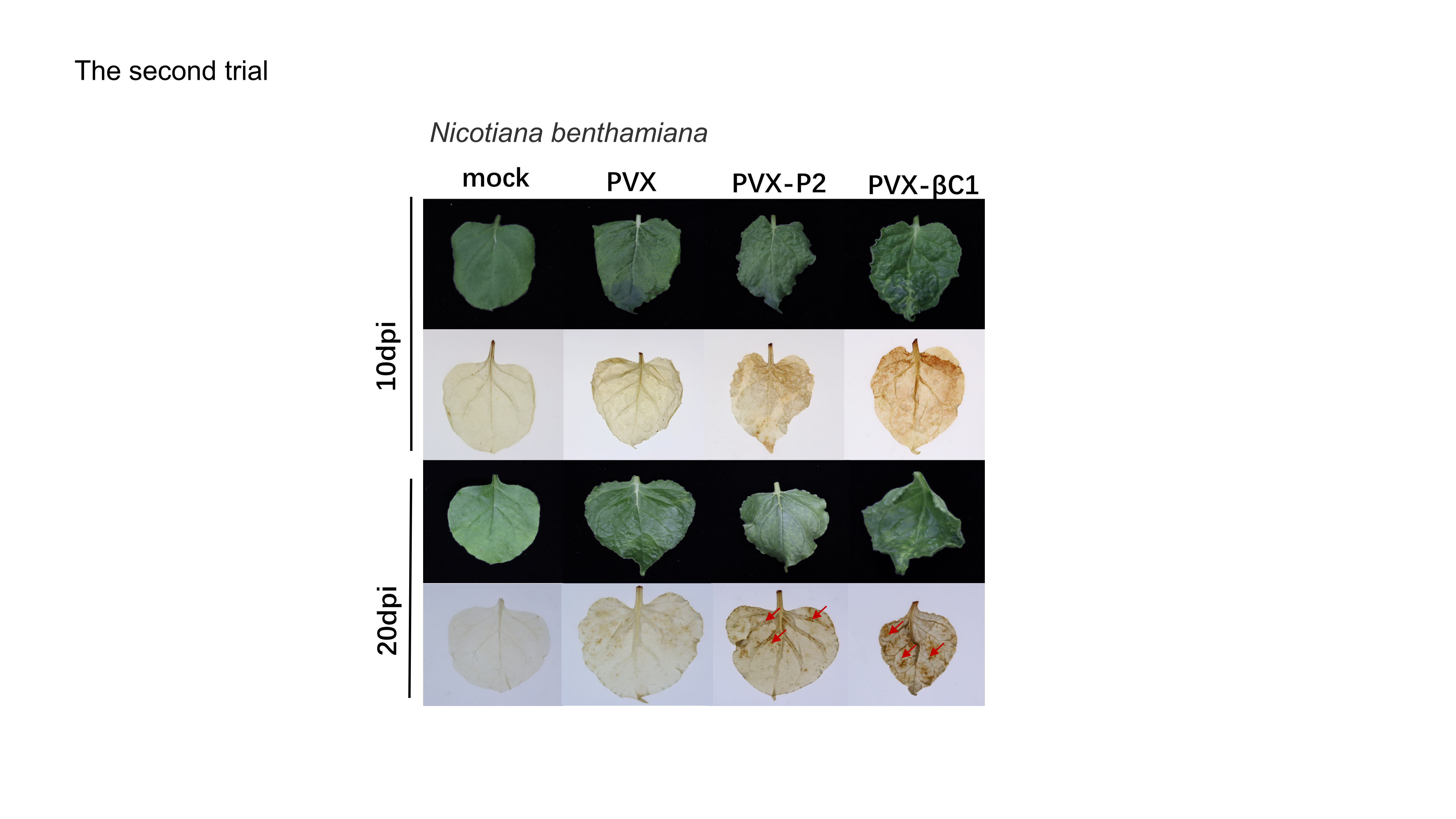

Supplement: Supplemental Information 13 [file peerj-12-16982-s013.zip › DAB staining/4.png]

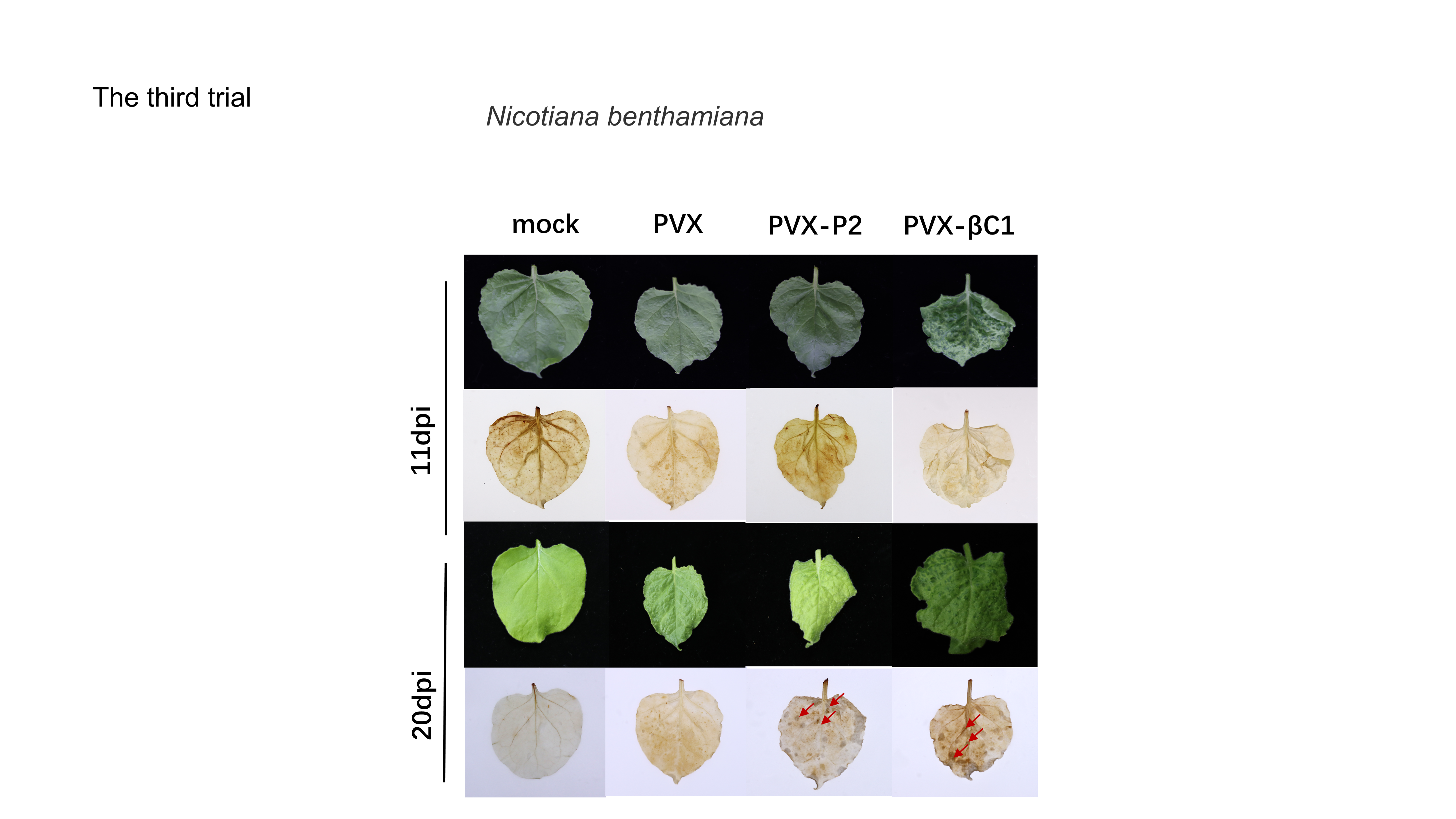

Supplement: Supplemental Information 13 [file peerj-12-16982-s013.zip › DAB staining/6.png]

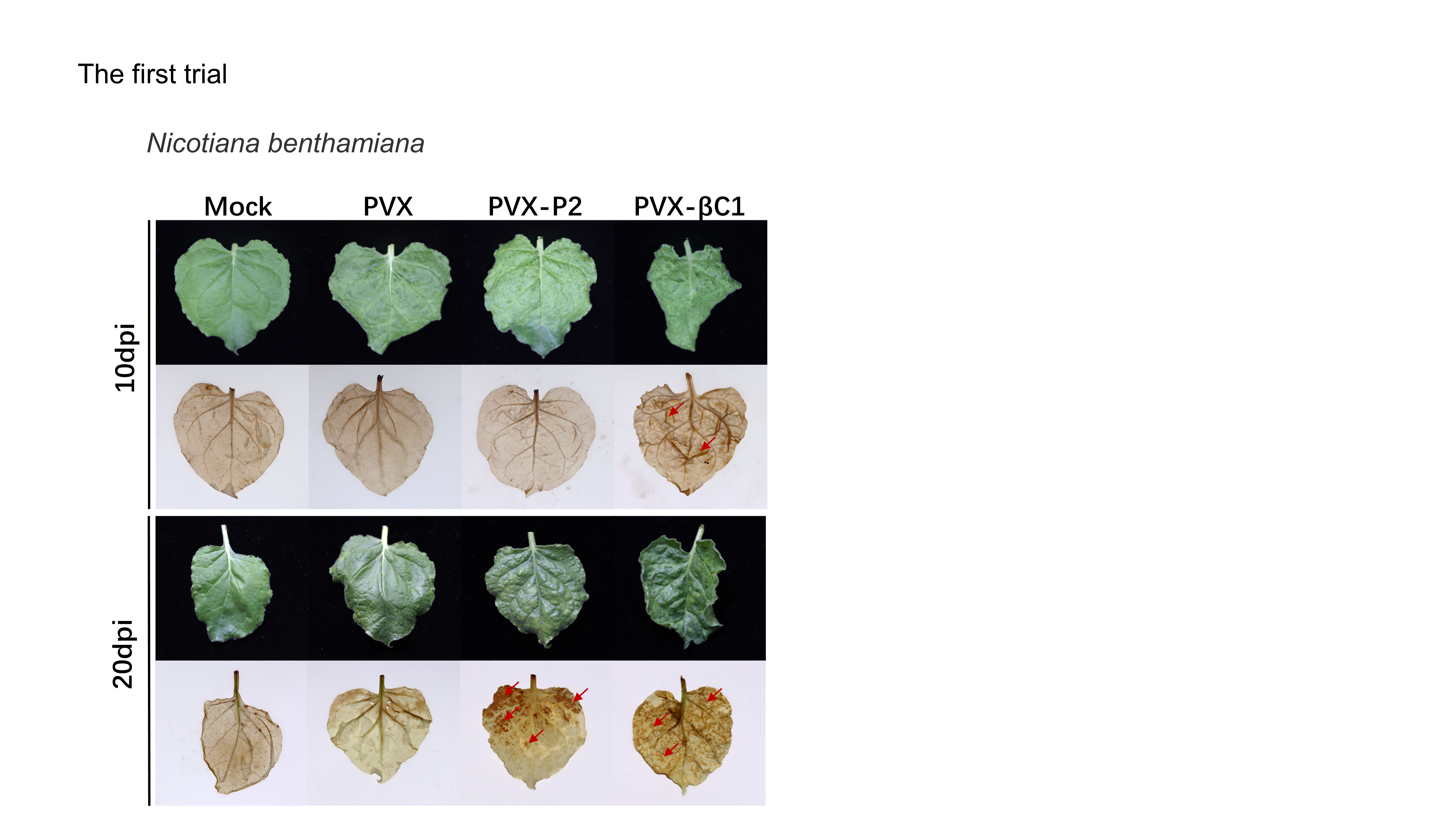

Supplement: Supplemental Information 13 [file peerj-12-16982-s013.zip › DAB staining/2.png]

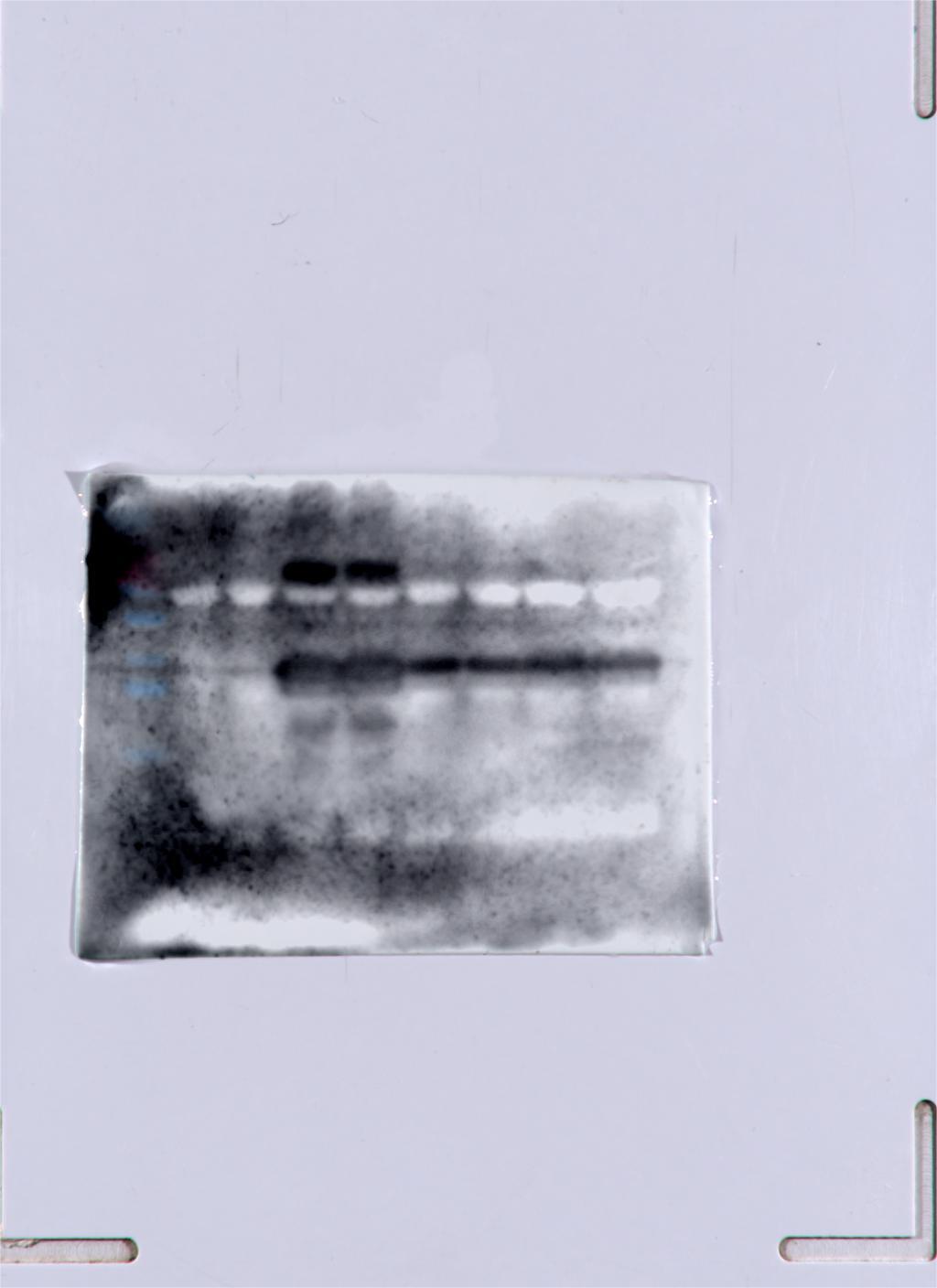

Supplement: Supplemental Information 14 [file peerj-12-16982-s014.zip › Western blot/PVX-P2 WB 1st trial/10dpi-a-CP.jpeg]

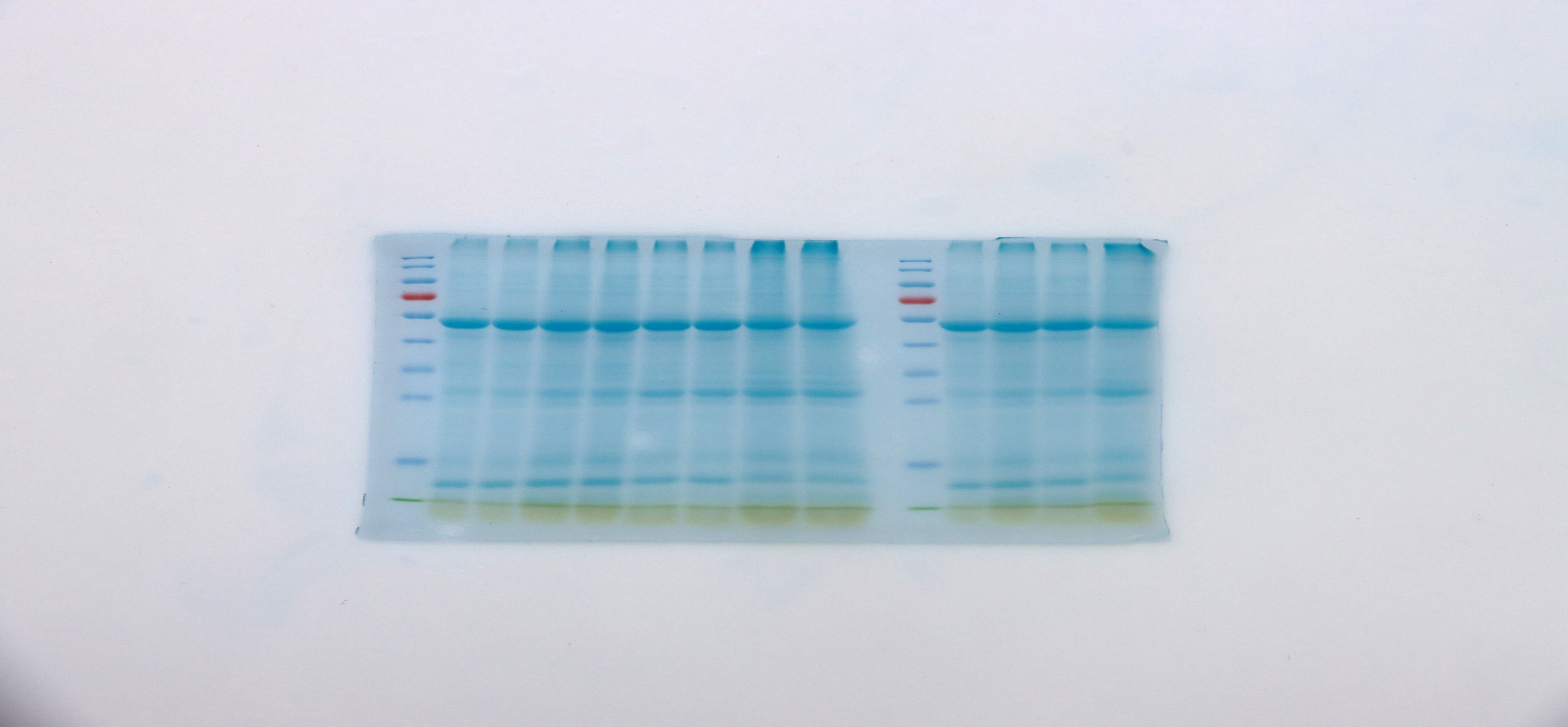

Supplement: Supplemental Information 14 [file peerj-12-16982-s014.zip › Western blot/PVX-P2 WB 1st trial/10dpi-Rubisco.png]

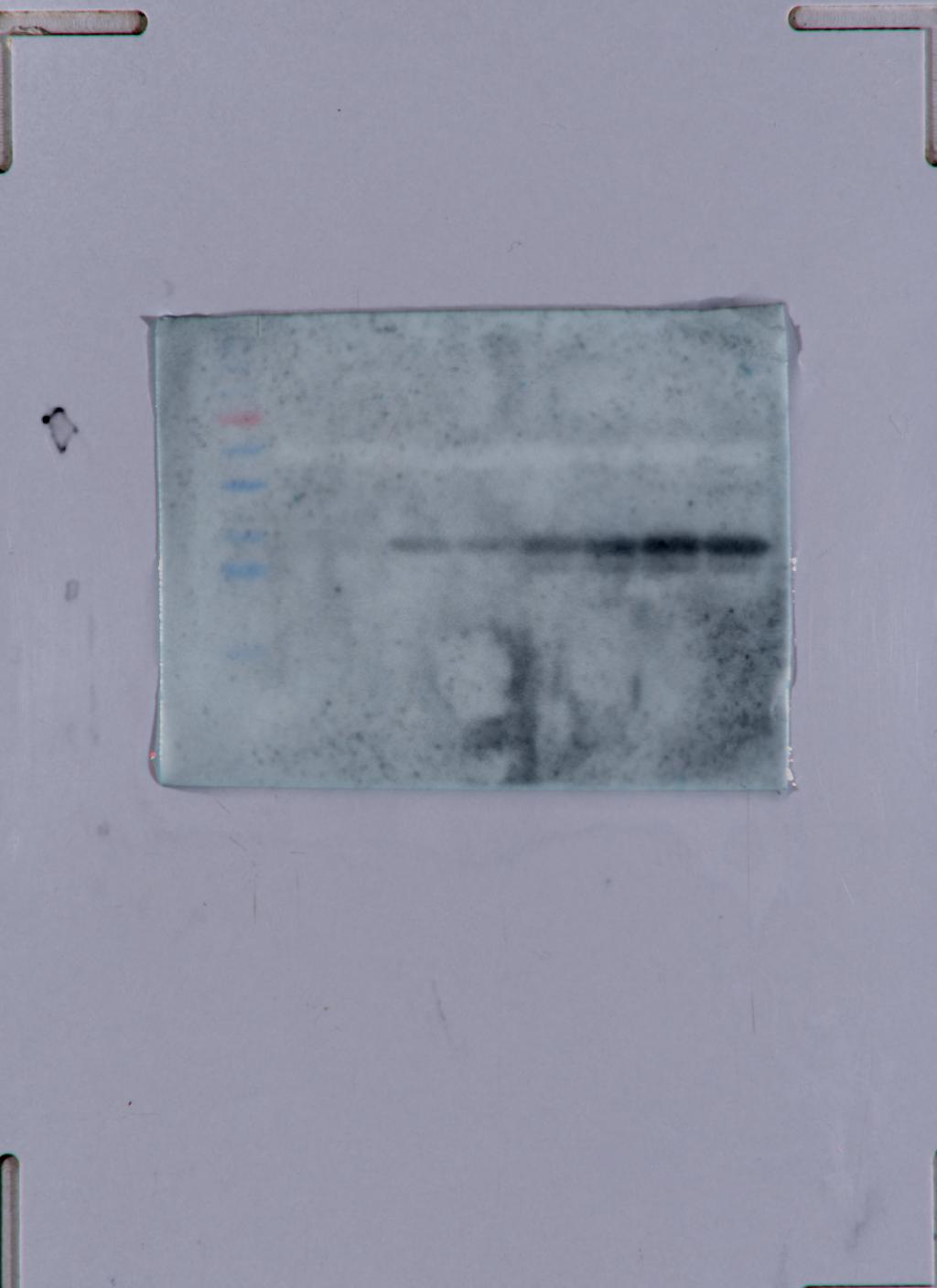

Supplement: Supplemental Information 14 [file peerj-12-16982-s014.zip › Western blot/PVX-P2 WB 1st trial/20dpi-a-CP.jpeg]

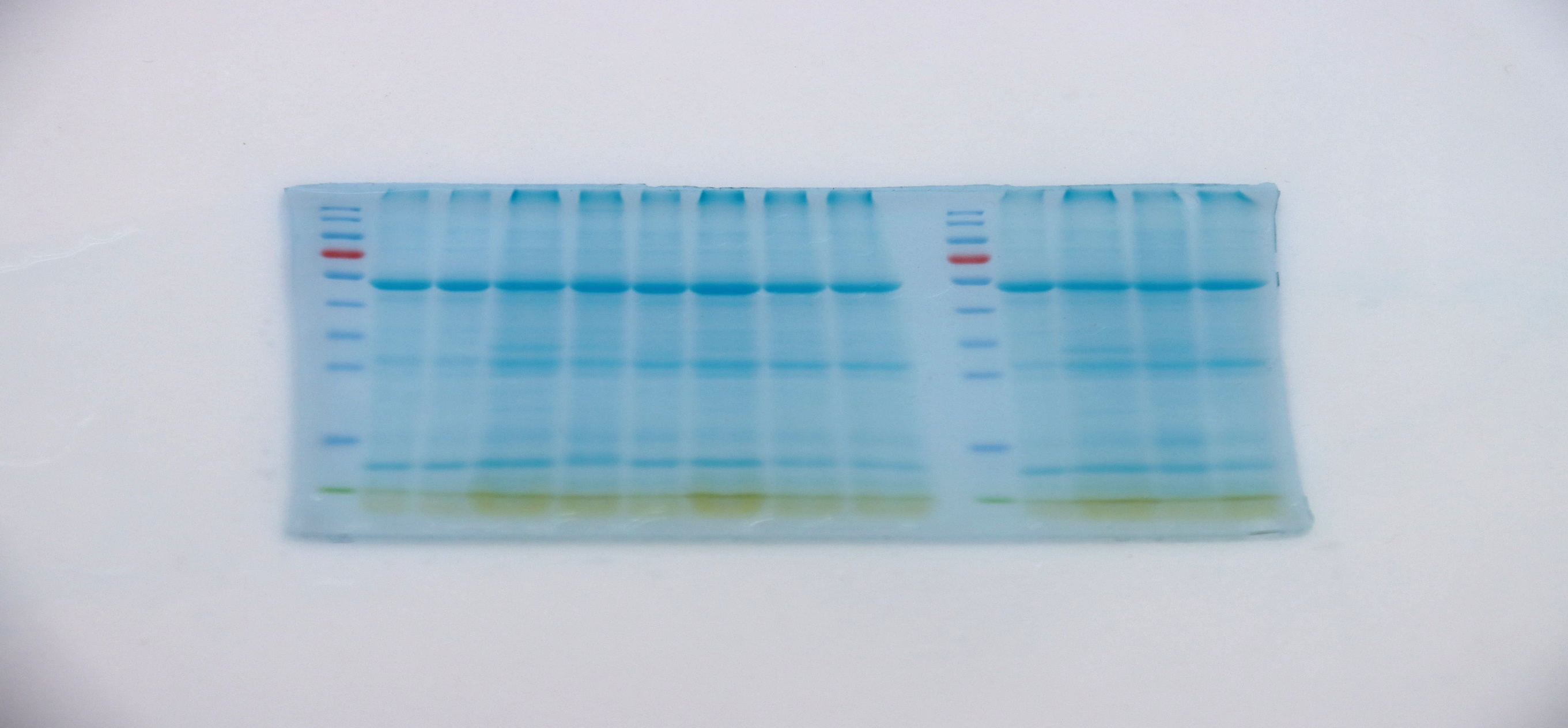

Supplement: Supplemental Information 14 [file peerj-12-16982-s014.zip › Western blot/PVX-P2 WB 1st trial/20dpi-Rubisco.png]

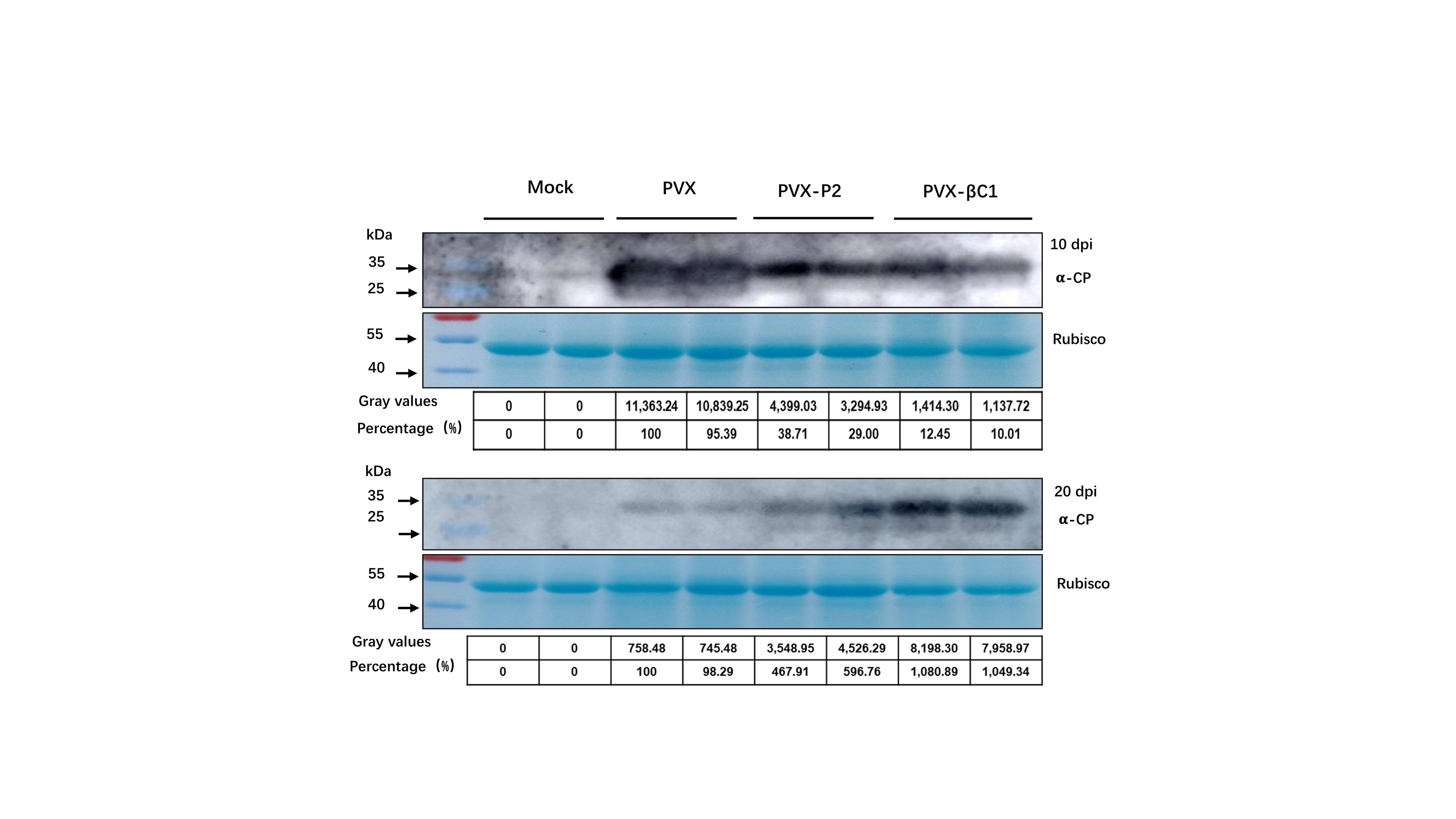

Supplement: Supplemental Information 14 [file peerj-12-16982-s014.zip › Western blot/PVX-P2 WB 1st trial/PVX-P2 WB.png]

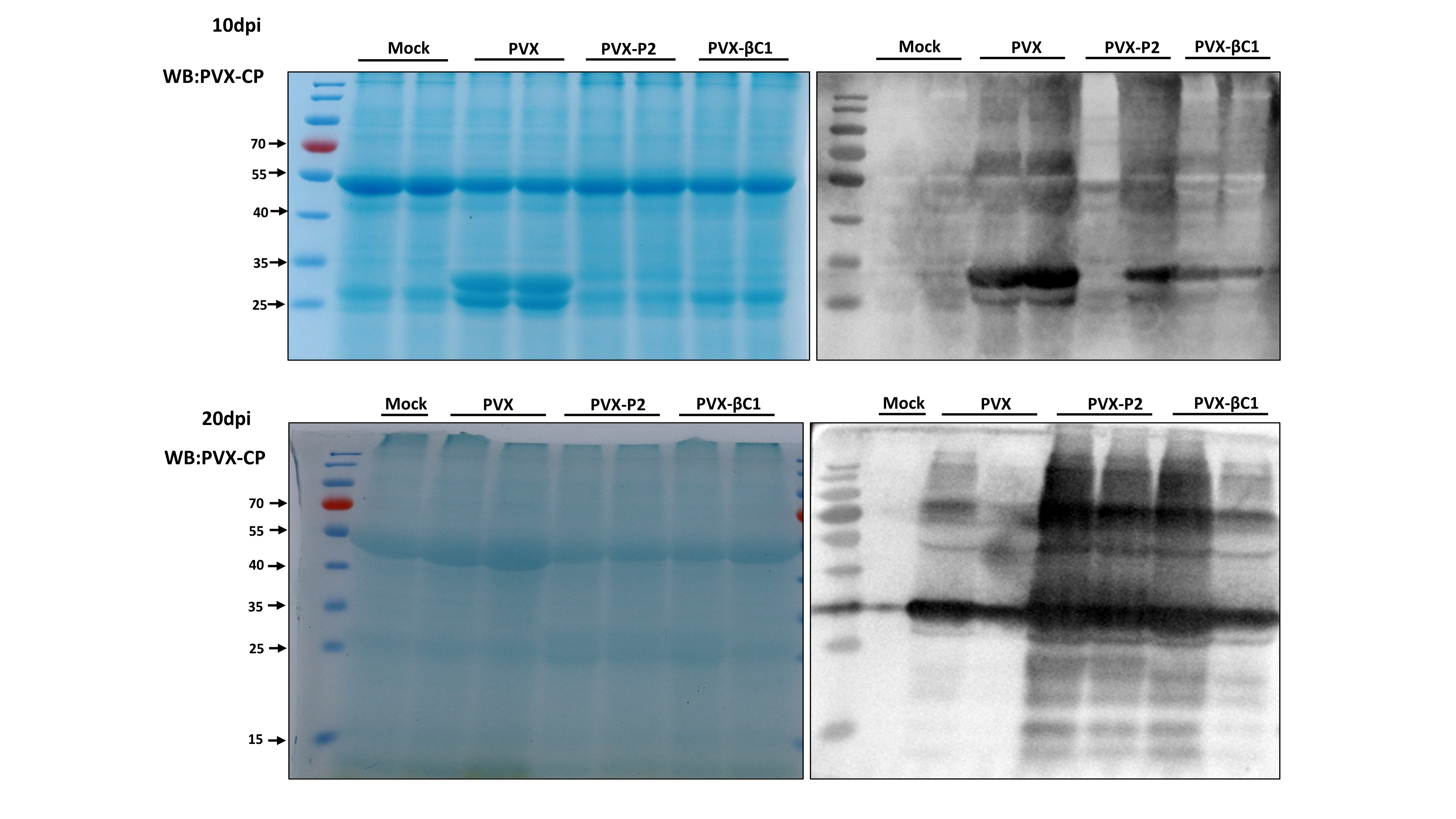

Supplement: Supplemental Information 14 [file peerj-12-16982-s014.zip › Western blot/PVX-P2 WB 2nd trial/PVX-P2 WB.png]

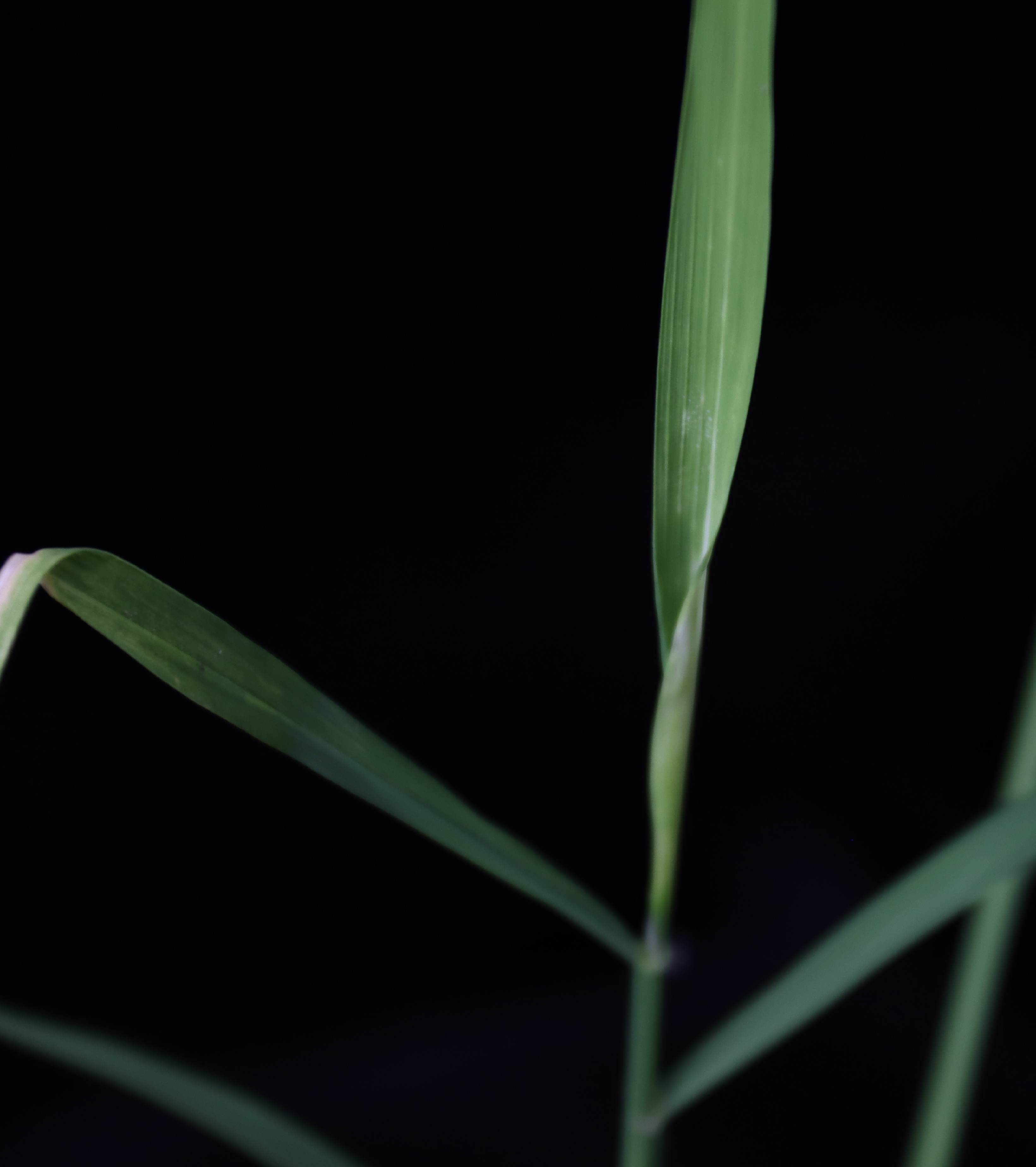

Supplement: Supplemental Information 15 [file peerj-12-16982-s015.zip › GV3101 pCB301-SCBV-enlarge.jpg]

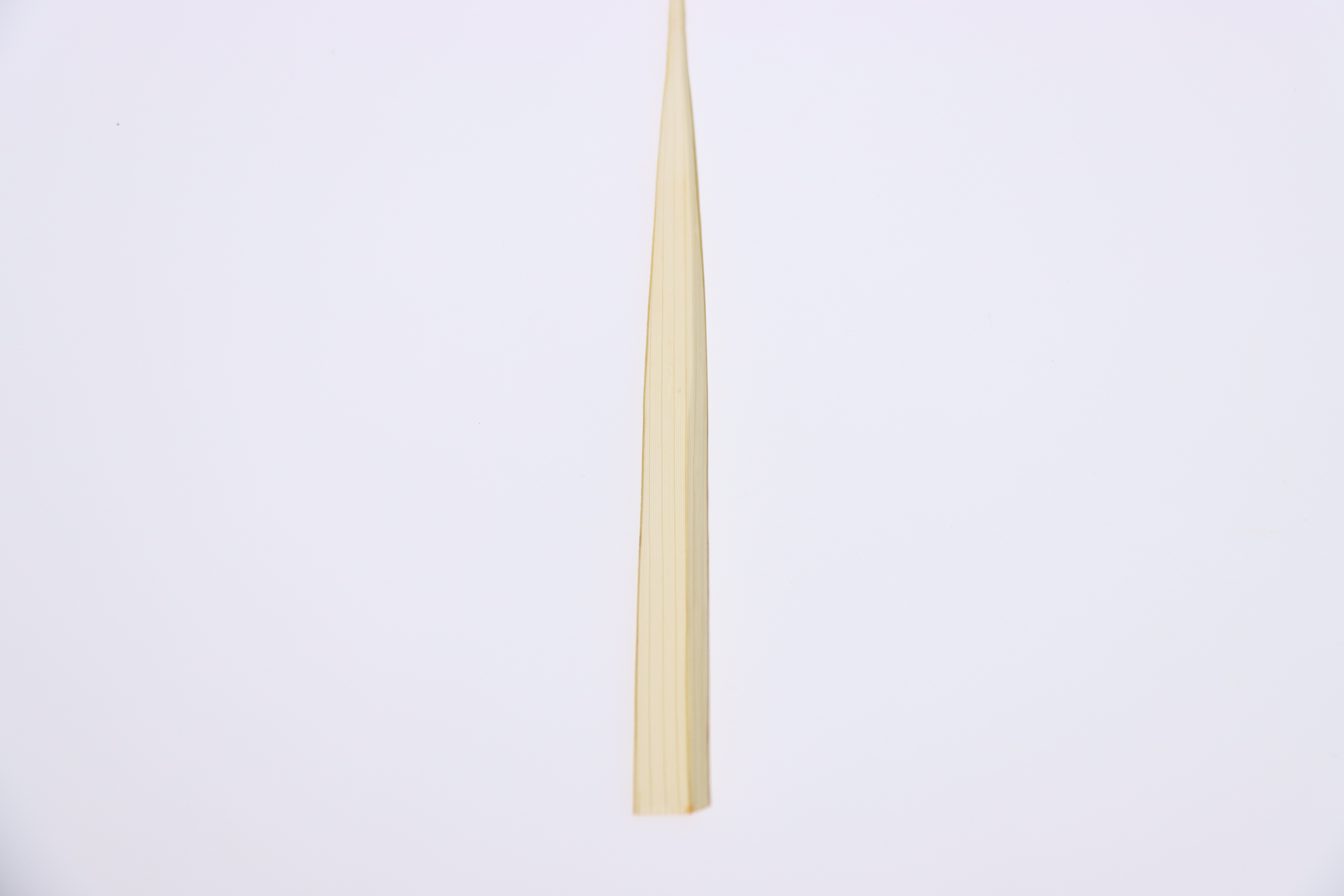

Supplement: Supplemental Information 15 [file peerj-12-16982-s015.zip › Mock DAB staining.JPG]

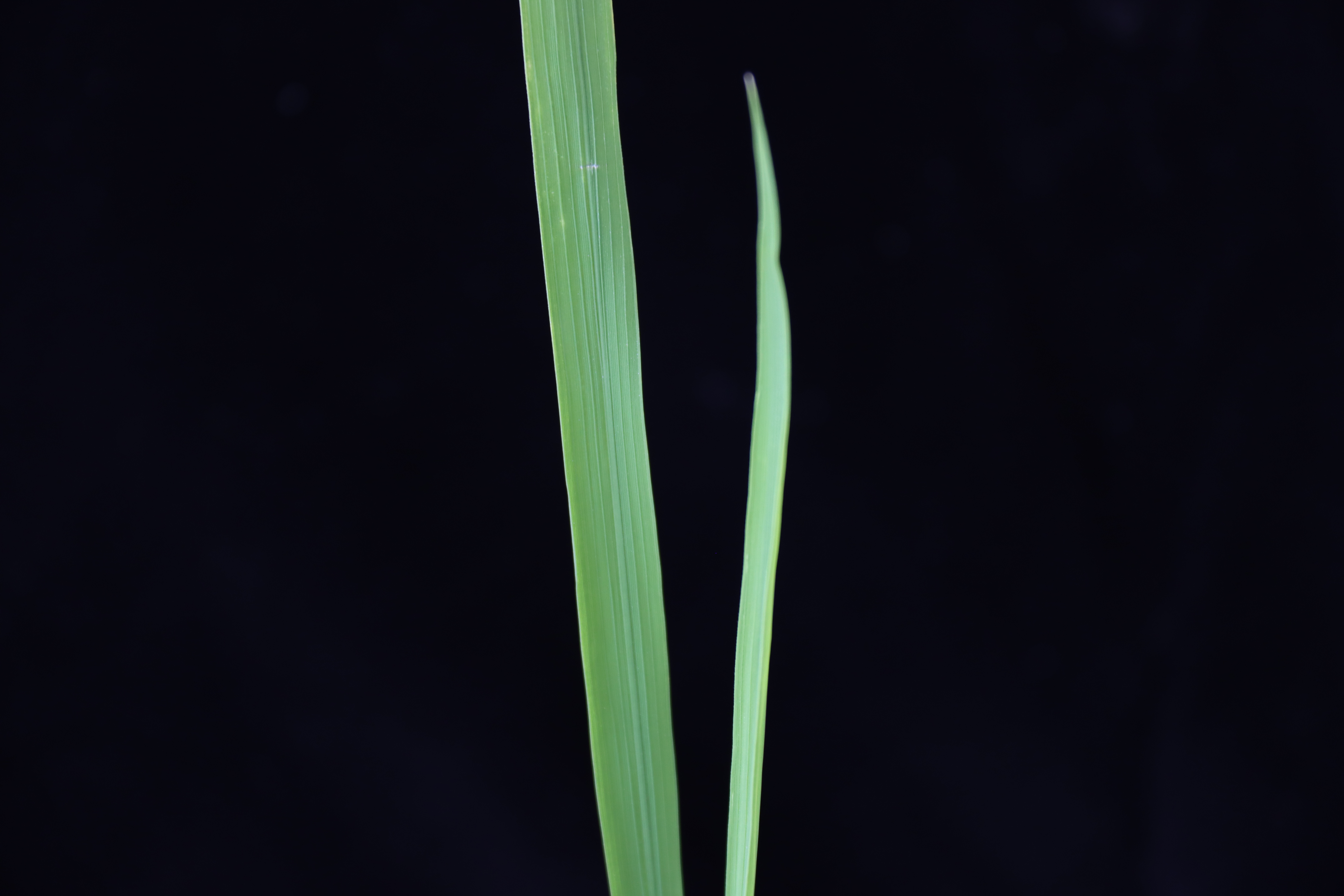

Supplement: Supplemental Information 15 [file peerj-12-16982-s015.zip › Mock enlarged.JPG]

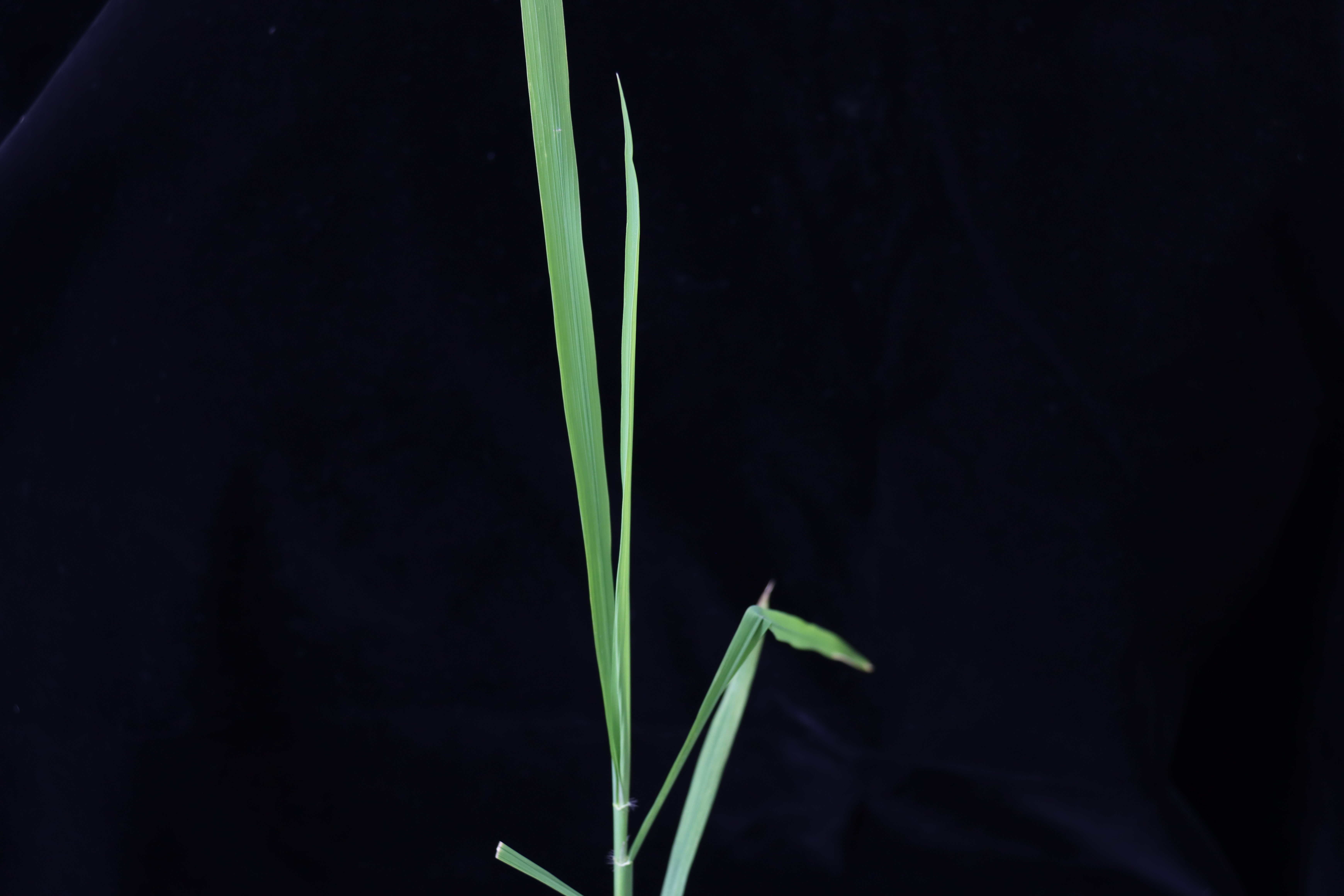

Supplement: Supplemental Information 15 [file peerj-12-16982-s015.zip › Mock.JPG]

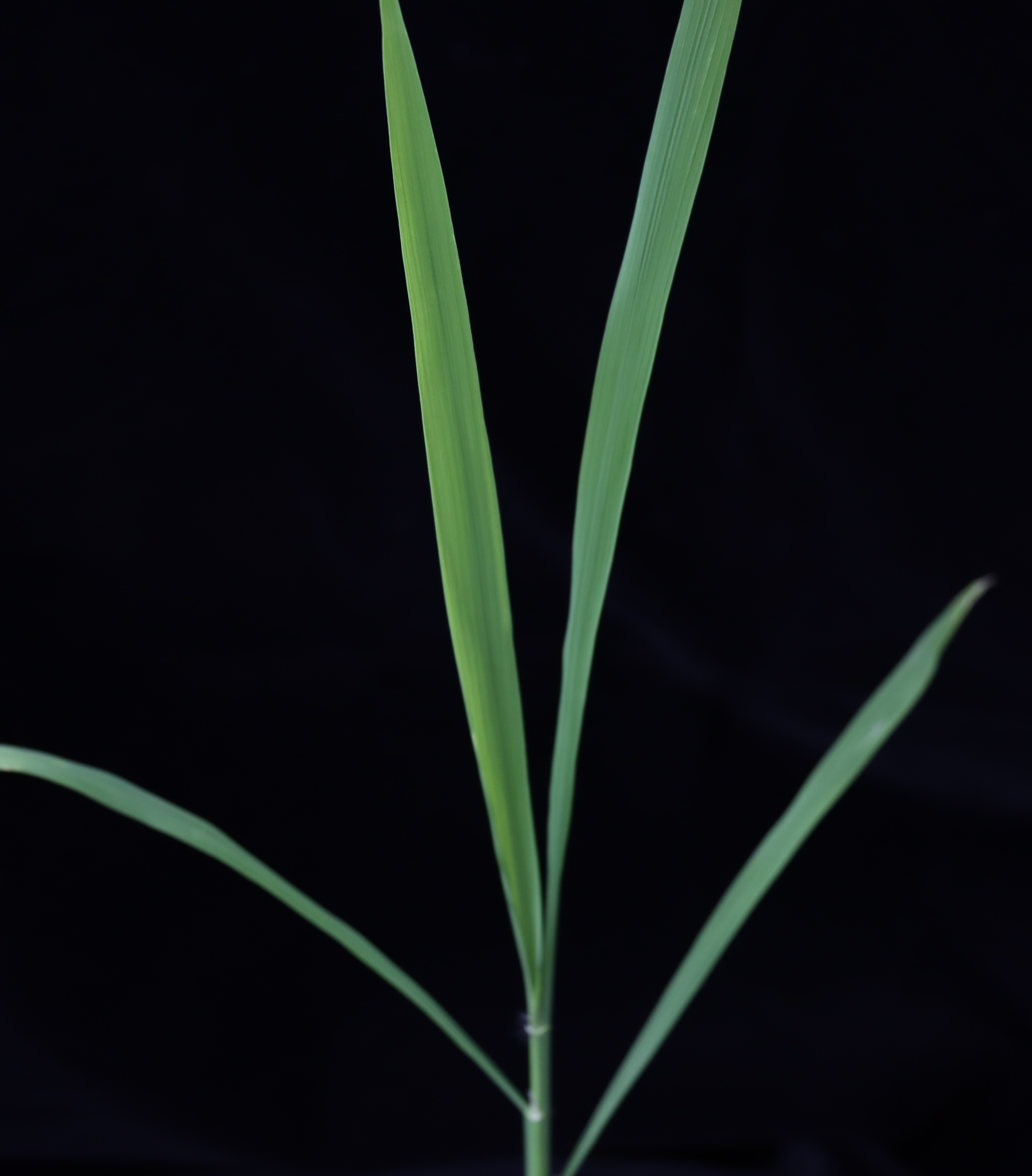

Supplement: Supplemental Information 15 [file peerj-12-16982-s015.zip › GV3101 pCB301.jpg]

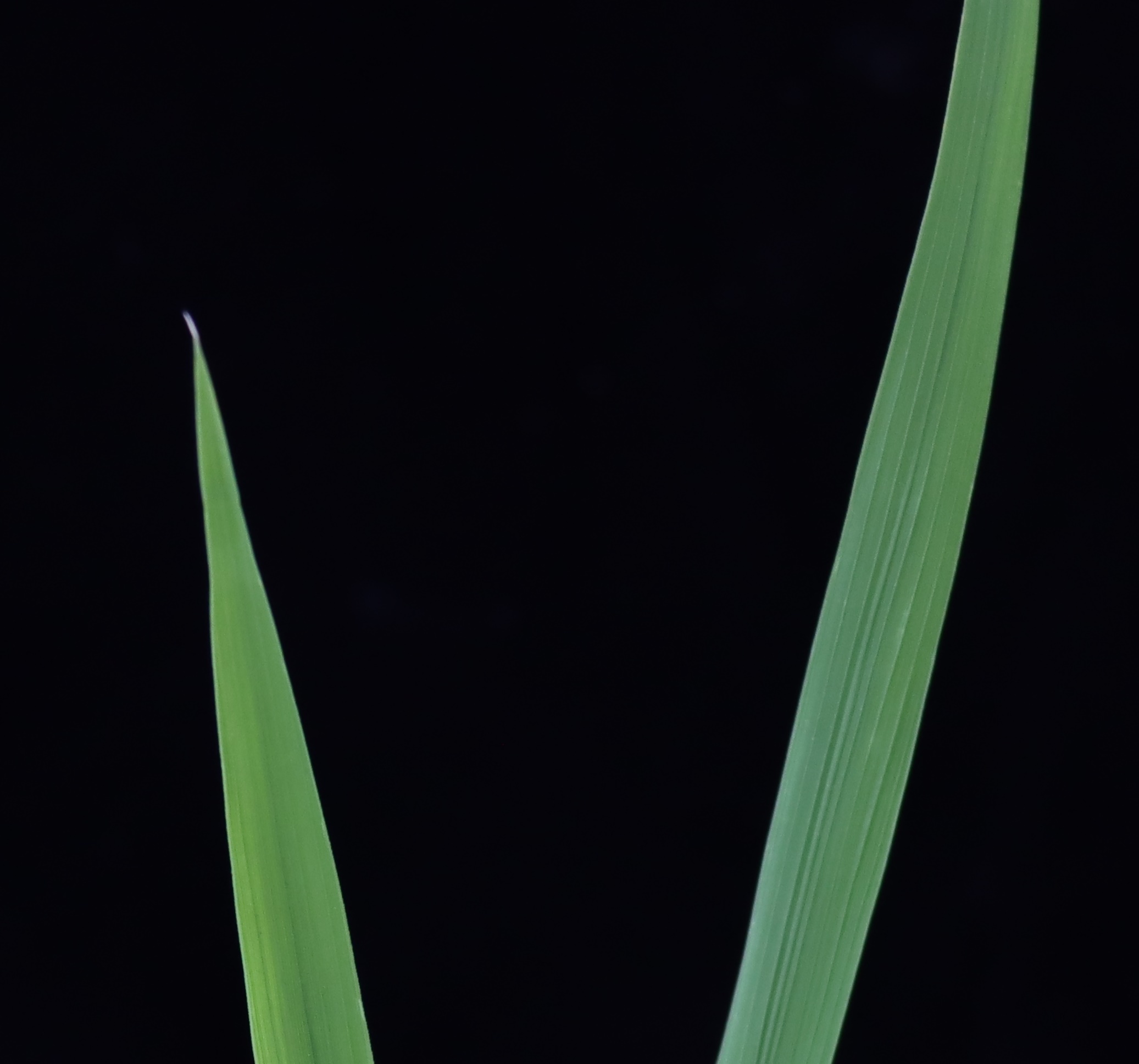

Supplement: Supplemental Information 15 [file peerj-12-16982-s015.zip › GV3101 pCB301-enlarge.jpg]

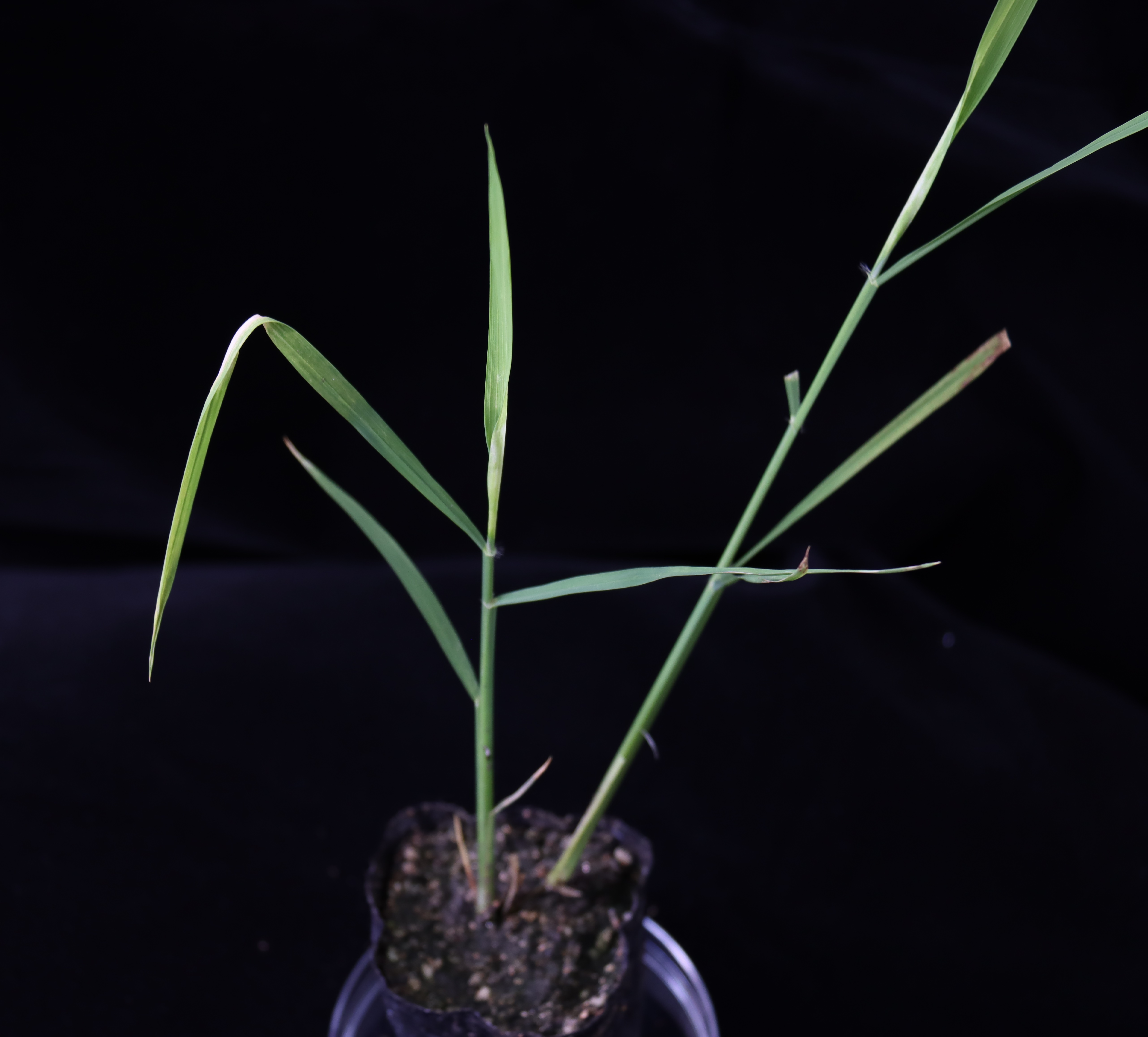

Supplement: Supplemental Information 15 [file peerj-12-16982-s015.zip › GV3101 pCB301-SCBV.jpg]

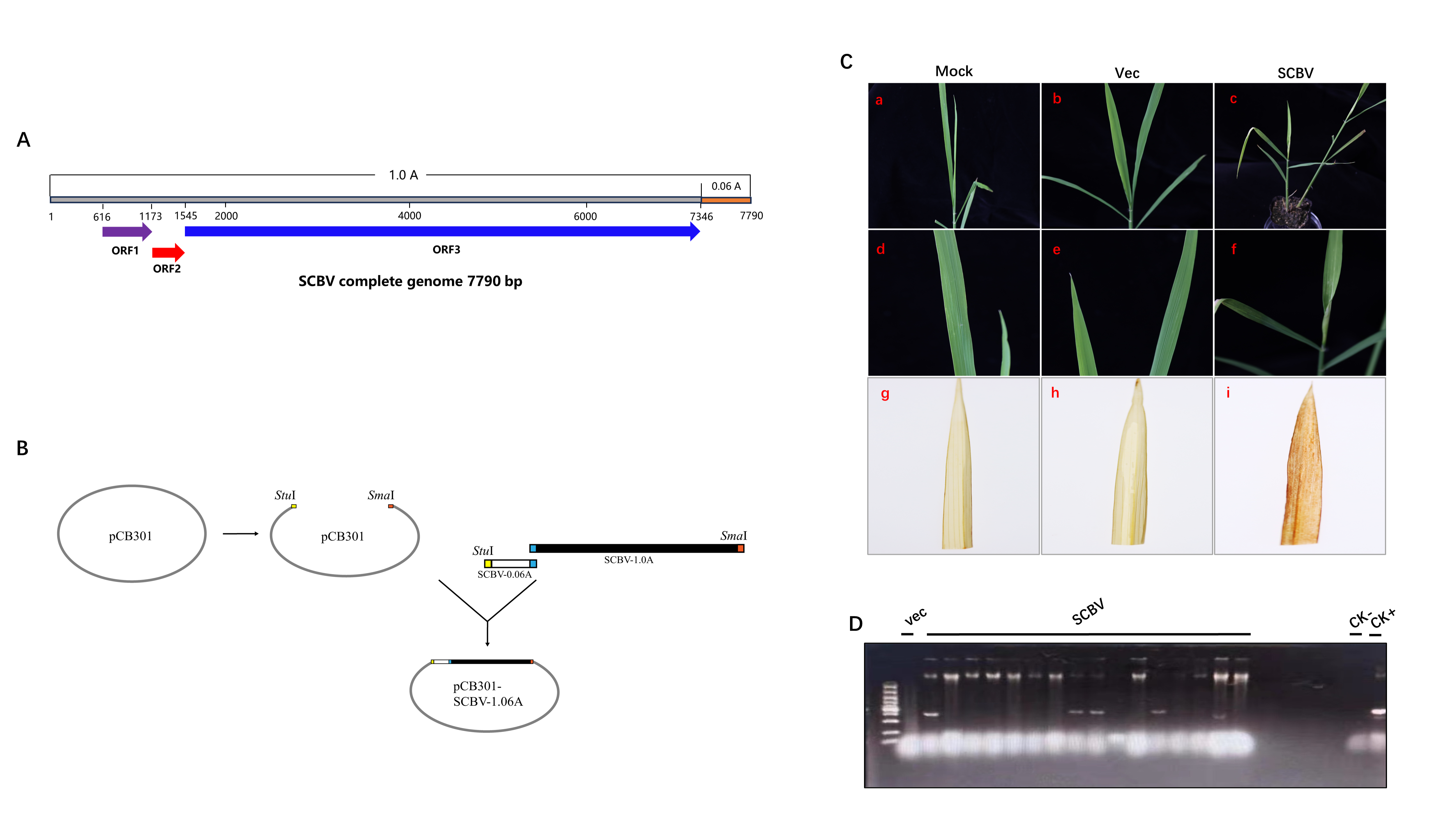

Supplement: Supplemental Information 15 [file peerj-12-16982-s015.zip › Figure S1.png]

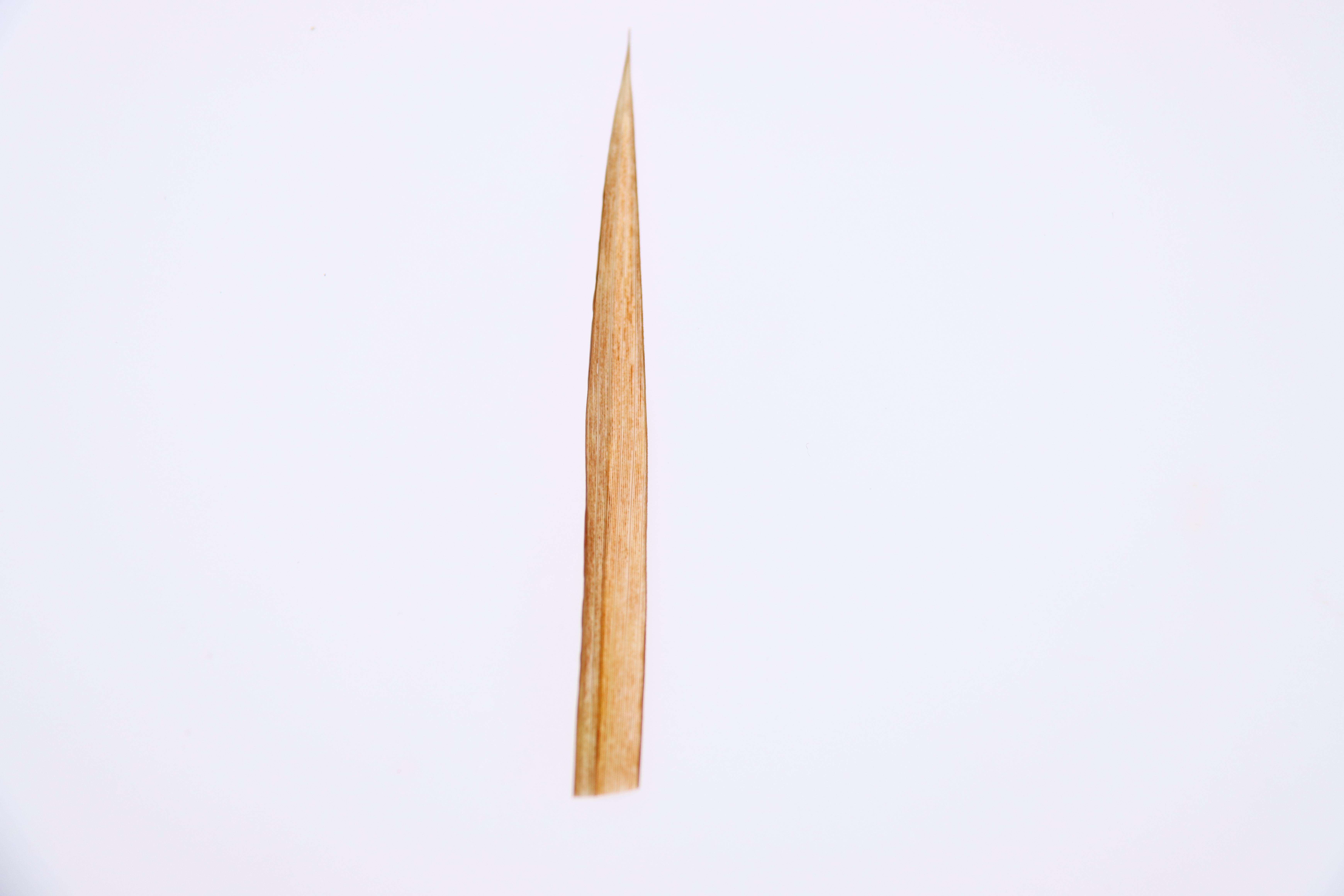

Supplement: Supplemental Information 16 [file peerj-12-16982-s016.zip › SCBV 2 DAB staining.JPG]

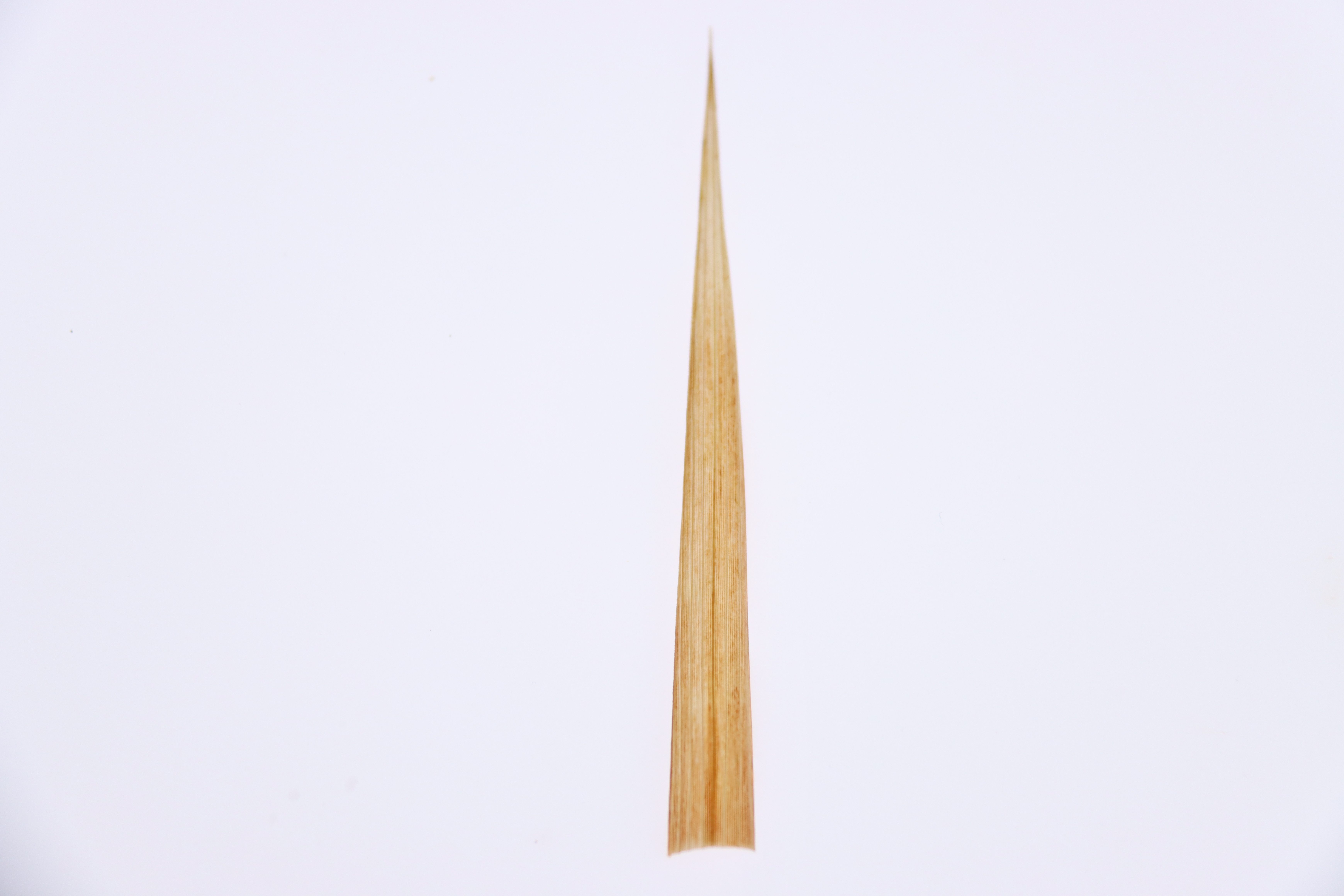

Supplement: Supplemental Information 16 [file peerj-12-16982-s016.zip › SCBV 3 DAB staining.JPG]

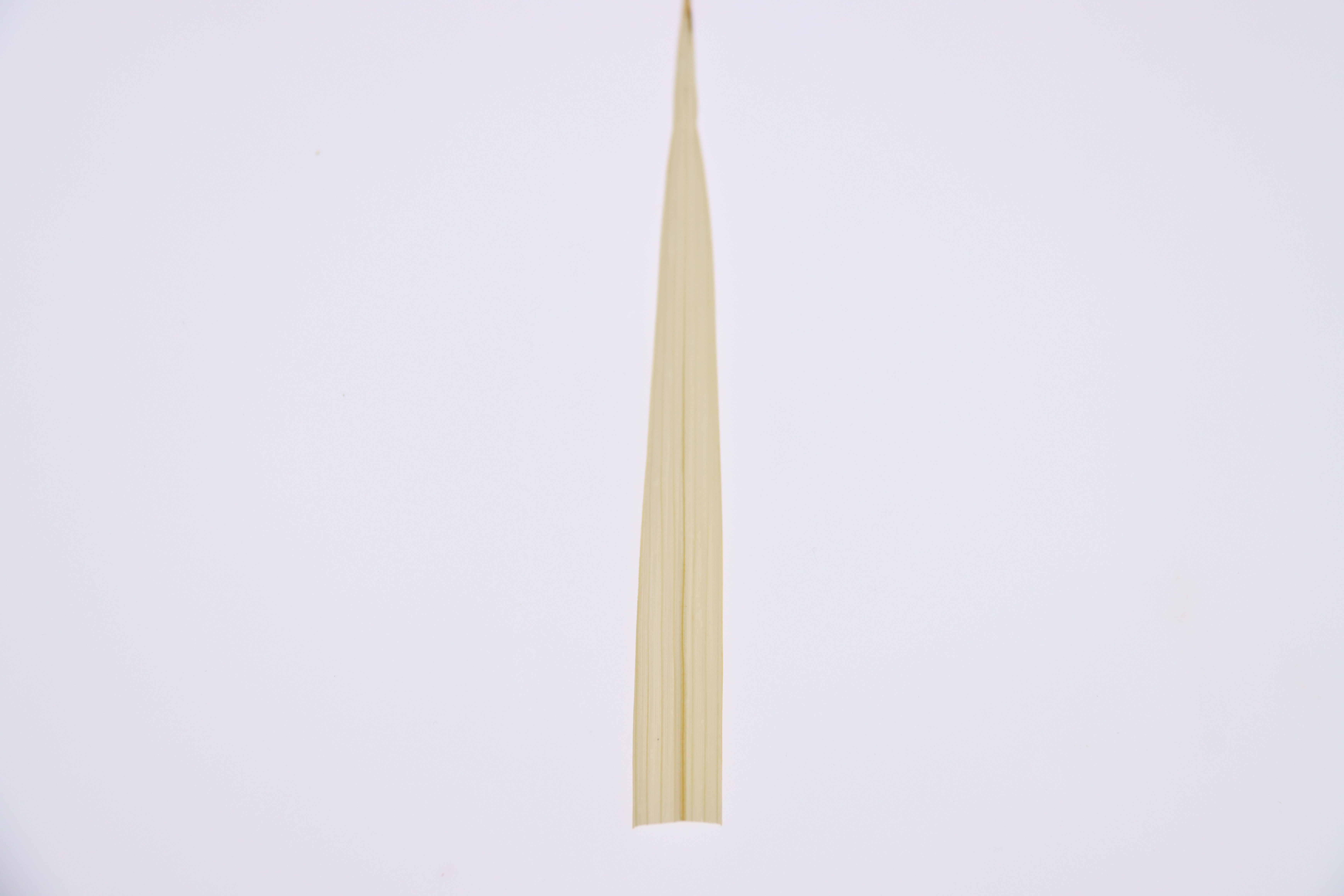

Supplement: Supplemental Information 16 [file peerj-12-16982-s016.zip › Vector DAB staining.JPG]

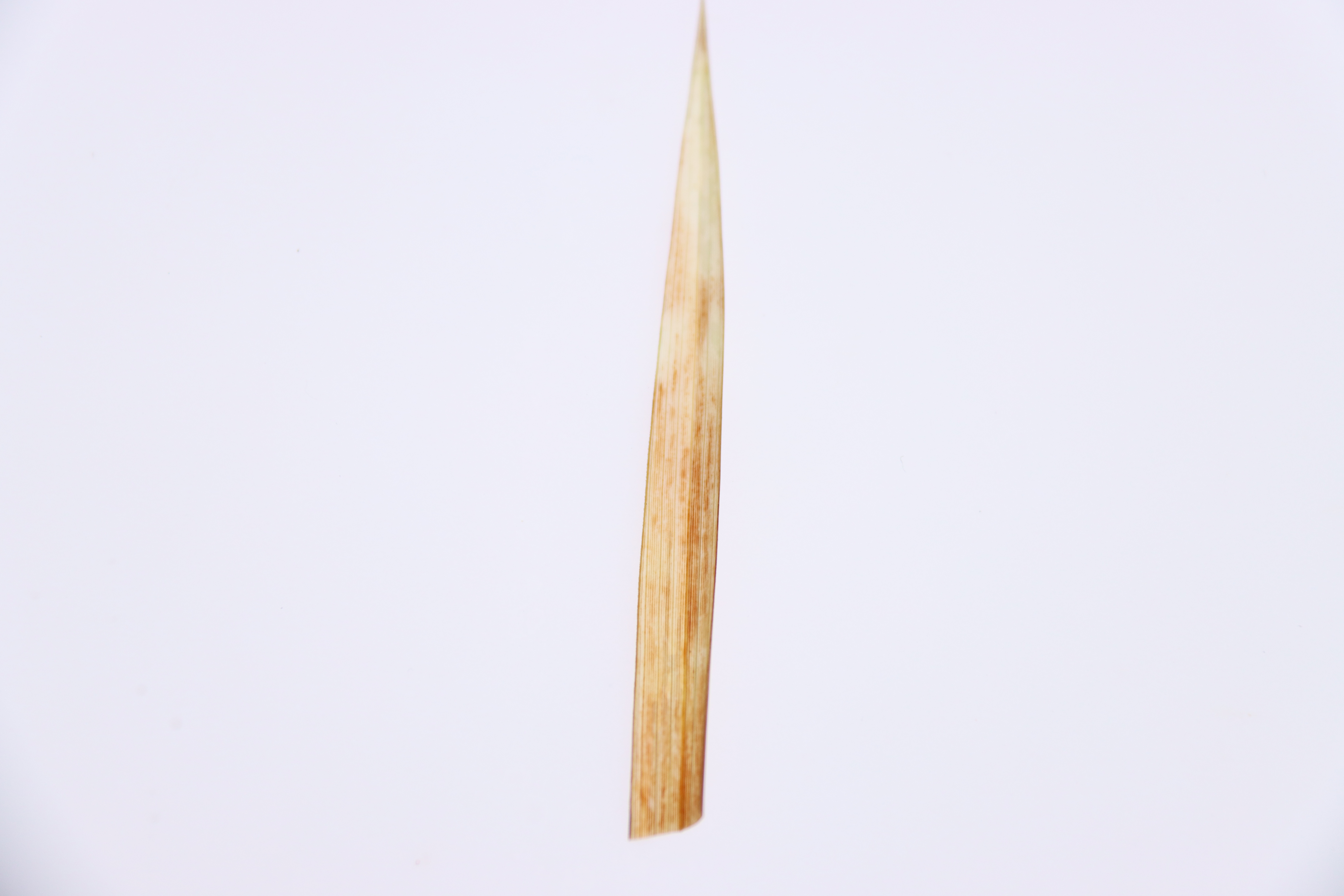

Supplement: Supplemental Information 16 [file peerj-12-16982-s016.zip › SCBV 1 DAB staining.JPG]
